# Titanocene / cyclodextrin supramolecular systems: a theoretical approach

**DOI:** 10.1186/1752-153X-6-129

**Published:** 2012-11-05

**Authors:** Adrian Riviş, Nicoleta G Hădărugă, Zeno Gârban, Daniel I Hădărugă

**Affiliations:** 1 Department of Food Science, Banat’s University of Agricultural Sciences and Veterinary Medicine, Faculty of Food Processing Technology, C. Aradului 119, Timişoara, 300645, Romania; 2 Department of Applied Chemistry and Organic-Natural Compounds Engineering, “Politehnica” University of Timişoara, Faculty of Industrial Chemistry and Environmental Engineering, Carol Telbisz 6, Timişoara 300001, Romania

**Keywords:** Metallocenes, Titanocenes, Cyclodextrins, Supramolecular systems, Molecular modelling, QSAR

## Abstract

**Background:**

Recently, various metallocenes were synthesized and analyzed by biological activity point of view (such as antiproliferative properties): ruthenocenes, cobaltoceniums, titanocenes, zirconocenes, vanadocenes, niobocenes, molibdocenes etc. Two main disadvantages of metallocenes are the poor hydrosolubility and the hydrolytic instability. These problems could be resolved in two ways: synthetically modifying the structure or finding new formulations with enhanced properties. The aqueous solubility of metallocenes with cytostatic activities could be enhanced by molecular encapsulation in cyclodextrins, as well as the hydrolytic instability of these compounds could be reduced.

**Results:**

This study presents a theoretical approach on the nanoencapsulation of a series of titanocenes with cytotoxic activity in α-, β-, and γ-cyclodextrin. The HyperChem 5.11 package was used for building and molecular modelling of titanocene and cyclodextrin structures, as well as for titanocene/cyclodextrin complex optimization. For titanocene/cyclodextrin complex optimization experiments, the titanocene and cyclodextrin structures in minimal energy conformations were set up at various distances and positions between molecules (molecular mechanics functionality, MM+). The best interaction between titanocene structures and cyclodextrins was obtained in the case of β- and γ-cyclodextrin, having the hydrophobic moieties oriented to the secondary face of cyclodextrin. The hydrophobicity of titanocenes (log*P*) correlate with the titanocene-cyclodextrin interaction parameters, especially with the titanocene-cyclodextrin interaction energy; the compatible geometry and the interaction energy denote that the titanocene/β- and γ-cyclodextrin complex can be achieved. Valuable quantitative structure-activity relationships (QSARs) were also obtained in the titanocene class by using the same log*P* as the main parameter for the *in vitro* cytotoxic activity against HeLa, K562, and Fem-x cell lines.

**Conclusions:**

According to our theoretical study, the titanocene/cyclodextrin inclusion compounds can be obtained (high interaction energy; the encapsulation is energetically favourable). Further, the most hydrophobic compounds are better encapsulated in β- and γ-cyclodextrin molecules and are more stable (from energetically point of view) in comparison with α-cyclodextrin case. This study suggests that the titanocene / β- and γ-cyclodextrin complexes (or synthetically modified cyclodextrins with higher water solubility) could be experimentally synthesized and could have enhanced cytotoxic activity and even lower toxicity.

## Background

Cancer is a generic name comprises a great number of medical affections, having various locations and symptoms [1-5]. Even this disease is studied more than fifty years, the cause and action mechanisms are not completely elucidated [3,6,7]. Chemotherapy is widely used in order to cure this disease, by using various cytostatic or cytotoxic compounds: alkylating agents, antimetabolites, hormones, immunostimulating agents, antibiotics, alkaloids, all of them with higher toxicity [1,8].

Organometallic compounds is an important class used in chemotherapy and the main groups studied are metallocenes (compounds which contains two cyclopentadienyl anions bound to a metal centre in the oxidation state II), ruthenium-, osmium-, iridium half-sandwich complexes, rhenium organometallics, metal *N*-heterocyclic carbene complexes, metal carbonyl complexes, or miscellaneous organometallic compounds [9]. The actual trend in cancer treatment is to replace some of the more toxic drugs such as cisplatin with less toxic compounds. Organometallic compounds are widely studied from cytotoxic point of view. Ferrocene was one of the first organometallic compounds from the first group evaluated for its antiproliferative properties [9,10]. Ferrocene derivatives were obtained as antimalarial or cytostatic drugs and drug candidates [9]. Recently, similar metallocenes were synthesized and analyzed by biological activity point of view: ruthenocenes, cobaltoceniums, titanocenes, zirconocenes, vanadocenes, niobocenes, molibdocenes etc. [9-23]. The titanocene compounds are promising such drugs, but the hydrolytic instability and slightly water solubility conduct to a lower cytotoxic activity (approximately ten fold lower than cisplatin). Further, in the titanocene series cytotoxic activity against HeLa, K562, and Fem-x cell lines increases with the overall hydrophobicity of compounds [16,17,22]. On the other hand, increasing the hydrophobicity of titanocenes conducts to a more lower water solubility and reducing the transport capacity in aqueous layers (even the transport capacity through lipid layers are increased) [16,17]. Despite of the resemblance of titanocene dichloride derivatives with cisplatin, seems that the mode of action as anti-cancerigene is different: binding to DNA and apoptosis of the cancer cell for the cisplatin and binding to DNA phosphate group, with additional interaction stabilizing the binding to DNA, for titanocenes. Two main problems of the titanocene dihalides are the poor hydrosolubility and the hydrolytic instability [22].

These problems could be resolved in two ways: synthetically modifying the titanocene structure (laborious, other physico-chemical and biological analyses needed) or finding new formulations with enhanced properties [11,13,15,19,24-27]. Natural or chemically modified cyclodextrins (cyclic oligosaccharides with hydrophobic inner cavities and hydrosolubilizing outer groups) are widely used for protection, enhancing water solubility, and controlled release properties of bioactive compounds [28-34]. The aqueous solubility of metallocenes (*i.e.* titanocenes) with cytostatic or cytotoxic activities could be enhanced by molecular encapsulation in cyclodextrins, as well as the hydrolytic instability of these compounds could be reduced (by reducing the access of water molecules to the metallocene halide reaction centre) [13,19,25,27].

This study presents a theoretical approach on the molecular encapsulation of a series of titanocenes with cytostatic activity in α-, β-, and γ-cyclodextrin, in order to obtain supramolecular systems with enhanced stability and bioavailability. Further, a quantitative structure-biological activity relationships (QSAR) studies were performed in order to evaluate the main parameters which influencing the *in vitro* cytostatic activity.

## Results and discussion

### Quantitative structure-activity relationships (QSARs)

**Scheme 1:**
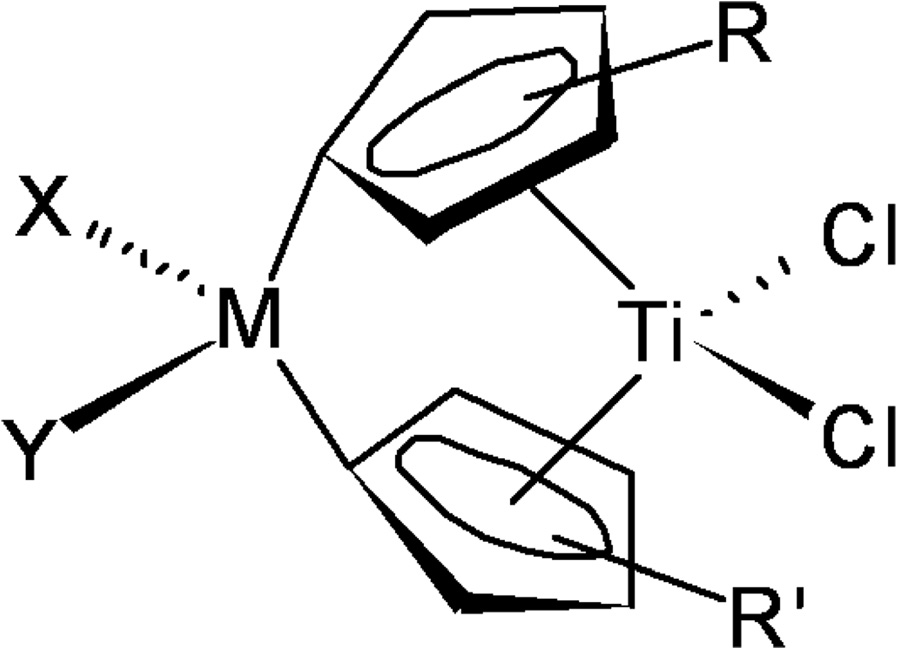
General structure of titanocene compounds.

**Figure 1:**
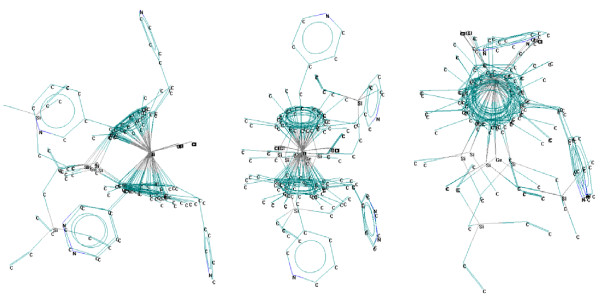
**Superimposed minimum energy conformations of titanocene structures **(**OX**, **OY**, **and OZ view**).

Molecular modelling and conformational analysis of titanocenes were performed by using the default parameters of MM+ functionality from the HyperChem 5.11 package [35]. This method is appropriate especially for organic molecules, but in our organometallic series the “inner” metal did not have a major influence on the overall geometry of compounds. Titanocenes with cytotoxic activity indicate a reduced number of stable conformations, especially for structures with a lower flexible bonds (codes 01TC, 02TC, 08TC to 10TC); structures having dimethyl-vinyl-silyl-ethyl or trivinyl-silyl-ethyl moieties at the Si, or pyridinium-methyl moieties at the cyclopentadienyl rings have a higher number of stable conformers; the most stable conformations are partially superimposed, especially at the titanocene skeleton. The most stable conformations have all flexible substituents (*Y*, *R*, and *R’* from the general structure, Table 1 and Scheme 1) oriented close to a pseudoplan formed by Ti and the gravity centres of the two cyclopentadiene rings (Figure 1) [see Additional file 1.

**Table 1:** **Titanocene structures (see Scheme 1) and *in vitro *cytotoxic activities**^**(a)**^

**No**	**Code**	**Structure**	**p*A***_***1***_	**p*A***_***2***_	**p*A***_***3***_
1	01TC	M: Si; X: CH_3_; Y: CH=CH_2_; R: all H; R’: all CH_3_	4.10	4.20	3.87
2	02TC	M: Si; X: CH_3_; Y: H; R: all CH_3_; R’: all CH_3_	3.96	4.23	3.93
3	03TC	M: Si; X: CH_3_; Y: (CH_2_)_2_Si(CH_3_)_2_(CH=CH_2_); R: all H; R’: all CH_3_	3.72	3.81	3.70
4	08TC	M: Si; X: CH_3_; Y: CH_3_; R: all H; R’: all CH_3_	3.87	4.18	4.02
5	09TC	M: Ge; X: CH_3_; Y: CH_3_; R: all H; R’: all CH_3_	3.81	4.14	3.97
6	10TC	M: Si; X: CH_3_; Y: H; R: all CH_3_; R’: all CH_3_	3.96	4.23	3.94
7	11TC	M: Si; X: CH_3_; Y: CH_3_; R: 3-CH_3_, 2,4,5-H; R’: all CH_3_	3.93	4.06	4.00
8	18TC	M: Si; X: CH_3_; Y: (CH_2_)_2_Si(CH=CH_2_)_3_; R: all H; R’: all CH_3_	3.70	3.70	
9	23TC	M,X,Y: none; R: 3-CH_2_(3-pyridinium); R’: all H	3.94		
10	24TC	M,X,Y: none; R: 3-CH_2_(3-pyridinium); R’: 3-CH_2_(3-pyridinium)	4.25		
11	26TC	M,X,Y: none; R: 3-CH_2_(4-pyridinium); R: 3-CH_2_(4-pyridinium);	4.97		

^(a) ^cytotoxic activity was expressed as the logarithm of the inverse inhibitory concentration 50%, p*IC*_*50*_ = log(1/*IC*_*50*_); p*A*_*1*_, p*A*_*2*_, p*A*_*3*_ were used for *in vitro *cytotoxic activity against HeLa, K562, and Fem-x cell lines, respectively.

In order to evaluate the importance of titanocene structure/conformation to the overall cytotoxic activity, the most important steric, electronic, and hydrophobic descriptors were evaluated by using *QSAR Properties* functionality. Molecular van der Waals surface and volume, refractivity, and polarizability for titanocenes in minimum energy conformations were evaluated from sterical descriptor class (Table 2), but some intercorrelation exists, especially in the case of *S*^*vdW*^ – *V*^*vdW*^ and *Rf* – *Pol* pairs (intercorrelation coefficient of 0.87 and 1.0, respectively, Table 3). This intercorrelation is poor for *S*^*vdW*^*/V*^*vdW*^ and *Rf/Pol*. Hydration energy has no correlation with descriptors from the other classes, but the transport parameter, log*P*, correlates with *Rf* and *Pol* parameters with higher intercorrelation coefficients (inverse correlation of −0.85 and −0.87, respectively, Table 3).

**Table 2:** **Values of the structural descriptors**^**(a)**^**for minimum energy conformations of titanocenes**

**No**	**Code**	***S***^***vdW***^	***V***^***vdW***^	***E***_***hydr***_	**log*P***	***Rf***	***Pol***
		(**Å**^**2**^)	(**Å**^**3**^)	(**kcal**/**mole**)		(**Å**^**3**^)	(**Å**^**3**^)
1	01TC	340	317	1.68	0.86	77.7	33.4
2	02TC	359	351	3.57	0.80	87.7	37.3
3	03TC	446	414	3.10	0.36	98.5	43.1
4	08TC	327	302	2.97	0.73	74.0	31.8
5	09TC	310	297	2.72	1.06	75.5	32.5
6	10TC	506	958	3.55	0.80	87.7	37.3
7	11TC	492	897	3.32	0.81	78.6	33.6
8	18TC	470	438	0.70	0.62	106.0	46.3
9	23TC	305	273	−3.02	0.51	74.6	30.4
10	24TC	401	361	−7.32	1.63	103.0	41.7
11	26TC	401	361	−7.85	1.63	103.0	41.7

^(a) ^van der Waals molecular surface (*S*^*vdW*^, Å^2^), van der Waals molecular volume (*V*^*vdW*^, Å^3^), hydration energy (*E*_*hydr*_, kcal/mole), logarithm of the octanol/water partition coefficient (log*P*), refractivity (*Rf*, Å^3^), and polarizability (*Pol*, Å^3^).

**Table 3:** Intercorrelational matrix for titanocene structural descriptors

	***S***^***vdW***^	***V***^***vdW***^	***E***_***hydr***_	**log*P***	***Rf***	***Pol***
***S***^***vdW***^	1.00	**0**.**91**	0.54	−0.40	0.52	0.49
***V***^***vdW***^		1.00	0.52	−0.02	0.17	0.13
***E***_***hydr***_			1.00	−0.23	0.43	0.38
**log*P***				1.00	−**0**.**77**	−**0**.**80**
***Rf***					1.00	**1**.**00**
***Pol***						1.00

Taking into account these intercorrelations, only log*P*, *Rf*, and *Pol* reveals statistically significant quantitative structure – cytotoxic activity relationships (QSARs) [36,37], which could drive molecular changes in this titanocene class in order to obtain new compounds with higher biological activity. In the case of HeLa cell line, *in vitro* cytotoxic activity of titanocenes correlates with the log*P* parameter (Eq. 1), having a correlation coefficient of 0.80, which is higher than in the other cases; the correlation coefficient for the case of K562 is little bit lower (Eq. 2), but statistically significant, as well as in the case of Fem-x cell line (Eq. 3). A significant correlation was also obtained if the polarizability, *Pol*, was used as independent parameter (Eq. 4); for example, an inverse correlation exists between cytotoxic activity against K562 cells (expressed as p*IC*_*50*_) and polarizability (*r* = 0.80), but this parameter is intercorrelated with the hydrophobic one. As a result, if the polarizability is lower, the hydrophobicity is increased and the transport, the interaction of titanocene with the hydrophobic cell membrane is favoured, and further the cytotoxic activity is increased. The predictive accuracy was evaluated by cross-validation method (leave-half-out method), all models having cross-validation correlation coefficients *q*^*2*^_*cv*_ > 0.75 [see Additional file 1. The predicted p*A* activities, calculated with equations Eq. 1 to Eq. 4, as well as the differences between experimental and predicted activities, are presented in Table 4 [see Additional file 1.

**Table 4:** **QSAR results for cytotoxic activity of titanocenes against HeLa**, **K562**, **and Fem**-**x cell lines **(**experimental activities** – ***A*and p*A***, **predicted activities **– **p*A***_*(****pred****.)*_, **and the differences between experimental and predicted activities**, **Δp*A***)

**No**^(**a**)^	**Code**	***A***_***1***_	***A***_***2***_	***A***_***3***_	**p*A***_***1***_^(**b**)^	**p*A***_***2***_^(**b**)^	**p*A***_***3***_^(**b**)^	**p*A***_***1***__*(****pred****.)*_^(**b**)^	**p*A***_***2***__*(****pred****.)*_^(**b**)^	**p*A***_***3***__*(****pred****.)*_^(**b**)^	**p*A***_***2****,****Rf***__*(****pred****.)*_^(**b**)^	**Δp*A***_***1***_^(**b**)^	**Δp*A***_***2***_^(**b**)^	**Δp*A***_***3***_^(**b**)^	**Δp*A***_***2****,****Rf***_^(**b**)^
		*(****IC***_***50***_*,*_***HeLa***_, μ**M**) ^(**b**)^	*(****IC***_***50***_*,*_***K562***_, μ**M**) ^(**b**)^	*(****IC***_***50***_*,*_***Fem****-****x***_, μ**M**) ^(**b**)^											
1	01TC	79.2±6.9	63.7±9.5	134.3±18.1	4.10	4.20	3.87	3.99	4.13	3.97	4.18	0.11	0.07	−0.10	0.02
2	02TC	108.6±8.6	59.4±8	116.3±8.7	3.96	4.23	3.93	3.95	4.09	3.94	4.04	0.01	0.14	−0.01	0.19
3	03TC	189±13.1	155.2±8.7	200	3.72	3.81	3.70	3.65	3.79	3.77	3.89	0.07	0.02	−0.07	−0.08
4	08TC	135±6	66±6	96±4	3.87	4.18	4.02	3.90	4.04	3.92	4.23	−0.03	0.14	0.11	−0.05
5	09TC	154±4	73±1	106±5	3.81	4.14	3.97	4.13	4.27	4.04	4.21	−0.32	−0.13	−0.07	−0.07
6	10TC	109±9	59±8	116±9	3.96	4.23	3.94	3.95	4.09	3.94	4.04	0.01	0.14	−0.00	0.19
7	11TC	117±3	88±4	101±9	3.93	4.06	4.00	3.96	4.10	3.95	4.17	−0.03	−0.04	0.05	−0.11
8	18TC	200	200		3.70	3.70		3.83	3.97		3.79	−0.13	−0.27		−0.09
9	23TC	114.2±57			3.94			3.75				0.19			
10	24TC	55.9±16.2			4.25			4.53				−0.28			
11	26TC	10.8±0.6			4.97			4.53				0.45			

^(a) ^Compounds No 1–3 are selected from reference [16], compounds No 4–8 from [17], and compounds No 9–11 from [22].

^(b)^*A* (*IC*_*50*_, μM) – the *in vitro* cytotoxic activity (±SD) of titanocenes selected from references [16,17,22]; p*A* – the logarithm of the inverse inhibitory concentration 50%, p*IC*_*50*_ = log(1/*IC*_*50*_); p*A*_*pred.*_ – the predicted cytotoxic activity (as the logarithm of the predicted inverse inhibitory concentration 50%, p*A*_*pred.*_ = p*IC*_*50, pred.*_ = log(1/*IC*_*50,pred.*_); Δp*A* – the difference between experimental and predicted activities (as the logarithm of the inverse inhibitory concentration 50%), Δp*A* = p*A* – p*A*_*pred.*_

QSAR results (Table 4 and Eqs. 1–4) indicate very good predictions of cytotoxic activity against HeLa and Fem-x cell lines, especially in the case of compounds 01TC-11TC (|Δp*A*| < 0.11, except compound 09TC for HeLa cytotoxic activity; this compound contains Ge as the second metal atom, in comparison with the other titanocene compounds, which contain only Si instead). Higher differences were observed in the case of pyridinium-methyl derivatives which have no Si as the second metal atom; the presence of Si atom in the structure stabilizes the overall titanocene structure (compounds 23TC, 24TC, and 26TC). Good results were obtained for prediction of the cytotoxic activity against K562 cell lines for models obtained with log*P* and *Rf* as structural parameters (Eqs. 2 and 4). The difference between experimental and predicted activity was lower than 0.2 in almost all titanocene cases (except compound 18TC, having trivinyl-silyl moiety and the highest *Rf* value) (Table 4). However, a correlation between predicted activities against K562 cell lines obtained with Eqs. 2 and 4 exists (*r* = 0.67); this result is sustained by the log*P* – *Rf* intercorrelation coefficient which are involved in Eqs. 2 and 4 (Table 3).

(1) $$\;pA_{1i}=3.40\left(\pm 0.17\right)+0.69\left(\pm 0.17\right)\cdot \log P_{i}$$

*n* = 11; *r* = 0.80; *F* = 16; *q*^*2*^_*cv*_ = 0.75

(2) $$\;pA_{2i}=3.54\left(\pm 0.23\right)+0.69\left(\pm 0.30\right)\cdot log\;P_{i}$$

*n* = 8; *r* = 0.70; *F* = 5.5; *q*^*2*^_*cv*_ = 0.88

(3) $$\;pA_{3i}=3.63\left(\pm 0.12\right)+0.39\left(\pm 0.16\right)\cdot log\;P_{i}$$

*n* = 7; *r* = 0.74; *F* = 5.9; *q*^*2*^_*cv*_ = 0.80

(4) $$\;pA_{2i}=5.27\left(\pm 0.37\right)-0.014\left(\pm 0.004\right)\cdot Rf_{i}$$

*n* = 11; *r* = 0.80; *F* = 10.5; *q*^*2*^_*cv*_ = 0.75

### Geometry optimization of titanocene / cyclodextrin supramolecular systems

It is known that the cytotoxic activity of titanocene dichloride is different from cisplatin action (the last being more toxic, but with higher cytotoxic activity): titanocenes conduct to adduct with DNA and prevent the replication and/or transcription, resulting in cell death [22]. Thus, the transport of titanocene to the DNA is very important, but the lower water solubility and instability (hydrolysis of chloride ligands) reduces the access to the target - biomacromolecules; on the other hand, if the hydrophobicity is increased (the QSAR study indicates a higher *in vitro* biological activity for more hydrophobic titanocenes), the titanocene transfer in aqueous layer is decreased (especially in the case of the *in vivo* experiments). Thus, the increase of the hydrophobicity in order to enhance the *in vitro* cytotoxic activity conducts to a less water solubility and a harder transport of the titanocenes to the target. The water solubility of hydrophobic molecules could be realized by molecular encapsulation in matrices such as cyclodextrins. Among the increasing of water solubility, the advantage of this procedure is to protect of easier hydrolysable titanocenes against degradation and controlled release to the target (*i.e.* DNA); further, the higher hydrophobicity of modified titanocenes conduct to a better interaction with the hydrophobic inner cavity of cyclodextrins.

Fully geometry optimization of titanocene / cyclodextrin supramolecular systems or docking of organometallic compounds in cyclodextrins [38,39] could provide information on the stability of complexes and suggest chemical modifications for new titanocenes with higher cytotoxic activity. In our theoretical experiments of fully geometry optimization of titanocene / cyclodextrin supramolecular systems in vacuum (the default HyperChem molecular mechanics MM+ force field was used), only the interaction of the hydrophobic moiety of titanocene with the inner cavity of cyclodextrins from the secondary face was efficient (higher stability of the complex). The main interactions which stabilize the titanocene / cyclodextrin complex in vacuum were bond stretching energy, the angle bending energy, the torsional energy, and the energy arising from van der Waals interactions of non-bonded pairs of atoms. Due to the fact that all titanocene compounds from the studied series are chemically similar molecules, we have an internal consistency from the force field point of view [15,40-49].

**Figure 2:**
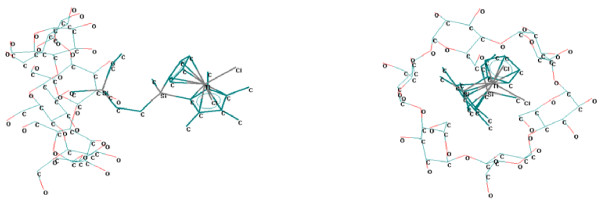
**Titanocene **(**code 03TC**) / **α**-**cyclodextrin supramolecular system **(**theoretically modeled by MM**+).

In the case of α-cyclodextrin, the interaction of titanocenes with the secondary face is poor, even with structures having thin hydrophobic chains (*e.g.* 03TC, 18TC, 23TC, 24TC, and 26TC; Figure 2) [see Additional file 1]; the worst interaction was observed for the case of titanocene oriented to the primary face of α-cyclodextrin or with the titanocene moiety in the front of both cyclodextrin rings. Theoretical interaction energy between titanocenes and α-cyclodextrin was in the range of 11.9 kcal/mole to 19.7 kcal/mole (Table 5). The best interactions of titanocenes with α-cyclodextrin were obtained in the case of structures having compounds monosubstituted at the Si (02TC and 10TC, *E*_*int.*_ 19.7 kcal/mole and 17.0 kcal/mole, respectively) or having flexible chain connected to this atom (03TC and 18TC, *E*_*int.*_ 18.0 kcal/mole and 17.0 kcal/mole, respectively). Lower interaction energies between titanocenes and α-cyclodextrin were obtained for compounds di-substituted at the Si atom with short chain moieties (methyl or vinyl) and for those substituted at the cyclopentadienyl rings with pyridinium-methyl moieties (Table 5). These low interaction energies are especially due to the geometric compatibilities, which are close to the limits and the interactions with the hydrophobic inner cavity of α-cyclodextrin are reduced. This is the reason that the hydrophobicity (expressed as log*P*) is not important (*r* < 0.5) in relation with the titanocene / α-cyclodextrin interaction energy, while the hydration energy (*E*_*hydr.*_) weak correlates with this interaction energy (Eq. 5).

(5) $$\begin{array}{ccc} \;E_{int.,aCD,i} & \;= & 15.54\left(\pm 0.57\right)\\ & & +0.356\left(\pm 0.137\right)\cdot E_{hydr.,aCD,i} \end{array}$$

**Table 5:** **Energies **(**resulted from the MM**+ **molecular modeling and titanocene**/**cyclodextrin optimization experiments**) **for cyclodextrins **(***E***_***CD***_, **α**-, **β**-, **and γ**-**cyclodextrin**, **codes aCD**, **bCD**, **and gCD**), **titanocenes** (***E***_***TC****.*_, **codes xTC**, **where x** = **01**–**03**, **08**–**11**, **18**, **23**, **24**, **and 26**), **the sum of titanocene and cyclodextrin energies**, **with no interaction** (***E***_***TC****.+****CD***_), **the energies of the TC**-**CD complex** (***E***_***TC****.-****CD complex***_), **and the TC**-**CD interaction energies** (***E***_***int****.*_), **determined as the difference between the TC**+**CD energy**, **with no interaction**, **and the energy of the TC**-**CD complex**

**No**	**Code**	***E***_***CD***_	***E***_***TC.***_	***E***_***TC.+CD***_	***E***_***TC.-CD complex***_	***E***_***int.***_
		**(kcal/mole)**	**(kcal/mole)**	**(kcal/mole)**	**(kcal/mole)**	**(kcal/mole)**
1	01TC_aCD		572.70	642.05	626.0	16.08
2	02TC_aCD		601.41	670.76	651.1	19.67
3	03TC_aCD		567.76	637.11	619.1	17.97
4	08TC_aCD		571.92	641.27	624.8	16.43
5	09TC_aCD		686.73	756.08	740.3	15.79
6	10TC_aCD	69.35	601.53	670.88	653.8	17.04
7	11TC_aCD		575.11	644.46	631.6	12.88
8	18TC_aCD		571.08	640.43	623.4	16.98
9	23TC_aCD		557.15	626.50	613.3	13.19
10	24TC_aCD		566.55	635.90	624.0	11.90
11	26TC_aCD		566.44	635.79	621.5	14.28
12	01TC_bCD		572.70	652.50	637.9	14.64
13	02TC_bCD		601.41	681.21	660.1	21.11
14	03TC_bCD		567.76	647.56	630.8	16.73
15	08TC_bCD		571.92	651.72	632.2	19.51
16	09TC_bCD		686.73	766.53	746.0	20.50
17	10TC_bCD	79.80	601.53	681.33	660.8	20.49
18	11TC_bCD		575.11	654.91	634.9	19.97
19	18TC_bCD		571.08	650.88	629.1	21.76
20	23TC_bCD		557.15	636.95	617.3	19.68
21	24TC_bCD		566.55	646.35	622.0	24.32
22	26TC_bCD		566.44	646.24	622.6	23.65
23	01TC_gCD		572.70	663.99	640.2	23.78
24	02TC_gCD		601.41	692.70	664.3	28.36
25	03TC_gCD		567.76	659.05	635.6	23.44
26	08TC_gCD		571.92	663.21	637.1	26.09
27	09TC_gCD		686.73	778.02	752.4	25.61
28	10TC_gCD	91.29	601.53	692.82	664.2	28.59
29	11TC_gCD		575.11	666.40	639.6	26.83
30	18TC_gCD		571.08	662.37	640.9	21.43
31	23TC_gCD		557.15	648.44	629.3	19.10
32	24TC_gCD		566.55	657.84	629.7	28.12
33	26TC_gCD		566.44	657.73	630.4	27.34

*n* = 11; *r* = 0.656; *F* = 6.8

**Figure 3:**
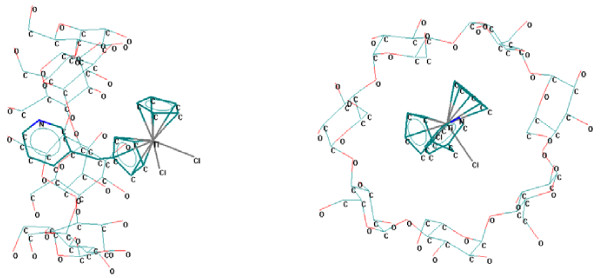
**Titanocene **(**code 23TC**) / **β**-**cyclodextrin supramolecular system **(**theoretically modeled by MM**+).

**Figure 4:**
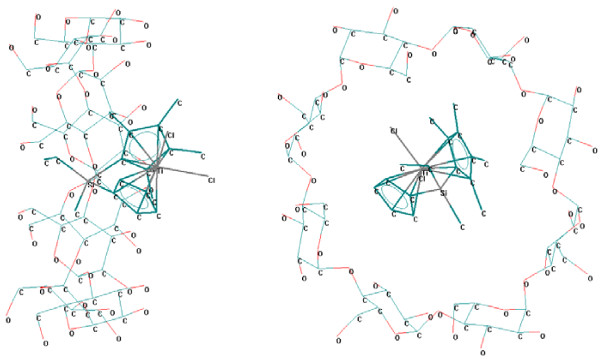
**Titanocene **(**code 01TC**) / **γ**-**cyclodextrin supramolecular system **(**theoretically modeled by MM**+).

The superior homologues, β- and γ-cyclodextrins, are more proper for molecular encapsulation of the studied titanocenes (Figures 3 and 4). In all β- and γ-cyclodextrin complexes the mono- or disubstituted silyl moiety, the bulky moiety (dimethyl-vinyl-silyl or trivinyl-silyl substituents) from the edge of the corresponding substituent, or the pyridinium-methyl substituent from the cyclopentadienyl ring are almost completely encapsulated in the cyclodextrin cavities [see Additional file 1]. In these cases the hydrophobicity of titanocenes (quantified by the logarithm of the 1-octanol/water partition coefficient) was important for the molecular encapsulation process and the interaction energy correlates with log*P* in a statistically significant way (Eq. 6 and Eq. 7). Thus, better interactions were obtained with the titanocenes having pyridinium-methyl substituents on the cyclopentadienyl rings (codes 24TC and 26TC), the interaction energy being 23.7-24.3 kcal/mole and 27.3-28.1 kcal/mole for β- and γ-cyclodextrin, respectively (Table 5). Higher interaction energies were obtained also for titanocenes having monosubstituted silyl moieties, 02TC and 10TC, especially in the case of γ-cyclodextrin complexes (28.4 kcal/mole and 28.6 kcal/mole, respectively; Table 5). Furthermore, a statistically significant correlation between titanocene / β- and γ-cyclodextrin interaction energies was observed (*r* = 0.82) [see Additional file 1].

(6) $$\begin{array}{ccc} \;E_{\mathrm{int}.,bCD,i} & \;= & 16.24\left(\pm 1.64\right)\\ & & +4.459\left(\pm 1.687\right)\cdot log\;P_{bCD,i} \end{array}$$

*n* = 11; *r* = 0.661; *F* = 7.0

(7) $$\begin{array}{ccc} \;E_{int.,gCD,i} & \;= & 21.46\left(\pm 1.99\right)\\ & & +4.347\left(\pm 2.047\right)\cdot log\;P_{gCD,i} \end{array}$$

*n* = 11; *r* = 0.578; *F* = 4.1

**Figure 5:**
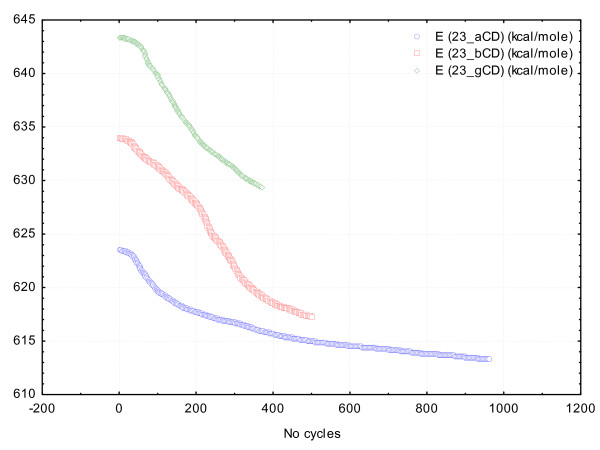
**Interaction energy *vs****.***Number of cycles from MM**+ **titanocene**/**cyclodextrin geometry optimization experiments for 23TC.**

The stability of the titanocene / cyclodextrin supramolecular system is observed from Figure 5 for β- and γ-cyclodextrin cases: the better stability is obtained in the case of γ-cyclodextrin, followed by β-cyclodextrin case, and finally for α-cyclodextrin complex after more than double number of iterations. This variation of the energy interaction reveals the possibility of forming the titanocene/cyclodextrin complexes and the stability of these supramolecular systems [see Additional files 1 and 2].

## Conclusions

In our study the importance of the hydrophobic parameter (log*P* – the logarithm of the octanol/water partition coefficient) in both of *in vitro* cytotoxic activity against HeLa, K562, and Fem-x cell lines, as well as in the cyclodextrin nanoencapsulation of the titanocene compounds were demonstrated.

Our theoretical studies demonstrates that the molecular encapsulation of titanocenes in natural cyclodextrins could resolves some of cytotoxic titanocene disadvantages: a more hydrophobic titanocene, which have higher cytotoxic activity (the *in vitro* cytotoxic activity against HeLa, K562, and Fem-x cell lines is increased with the log*P* of the titanocene compound; *r* > 0.7), is better encapsulated in cyclodextrins and could be transported through the aqueous layers (cyclodextrin inclusion compounds are water soluble) and protected against hydrolysis. Our theoretical study on the titanocene / cyclodextrin complexes indicate that the hydrophobic biologically active compounds (with higher cytotoxic activity) are better encapsulated in β- and γ-cyclodextrin; the highest titanocene / β- and γ-cyclodextrin complex interaction energies of ~24 kcal/mole and ~28 kcal/mole was obtained in vacuum for bis(pyridinium-methyl)-silyl derivatives, respectively. This study indicate that the titanocene compound can be controlled released to the target from the cyclodextrin complex; this complex allow to transfer the more hydrophobic titanocene through lipid layers, to increase the concentration of bioactive compound to the DNA phosphoester groups and further to form the titanocene-DNA adducts. Due to this process the inhibition of DNA transcription and/or replication appears (cytotoxicity).

According to our theoretical study, these titanocene/cyclodextrin inclusion compounds can be obtained (the encapsulation process is energetically favourable for β- and γ-cyclodextrin complexes). Further, the most hydrophobic compounds are better encapsulated in β- and γ-cyclodextrin molecules and are more stable (from energetically point of view) in comparison with α-cyclodextrin case. This study suggests that the titanocene/β- and γ-cyclodextrin complexes (or synthetically modified cyclodextrins with higher water solubility) could be experimentally synthesized and could have enhanced cytotoxic activity and even lower toxicity.

## Methods

### Titanocene structure selection and cytotoxic activity

Titanocenes with potential anticarcinogenic properties were recently synthesized by Gómez-Ruiz *et al.*[16,17] and Potter *et al.*[22] and have structural variability at the cyclopentadienyl moieties. All these new titanocene structures (eleven organometallic compounds) with cytotoxic activity against cervical carcinoma cell line HeLa, human myelogenous leukemia cell line K562, and human malignant carcinoma cell line Fem-x were considered in this theoretical study. Cytotoxic activity was expressed as the logarithm of the inverse inhibitory concentration 50%, p*IC*_*50*_ = log(1/*IC*_*50*_); p*A*_*1*_, p*A*_*2*_, p*A*_*3*_ were used for cytotoxic activity against HeLa, K562, and Fem-x, respectively (Table 1).

### Molecular modelling

Molecular modelling of titanocene molecules as well as α-, β-, and γ-cyclodextrins was performed by using the molecular mechanics MM+ functionality from the HyperChem 5.11. The MM+ molecular mechanics force field with a RMS of 0.005 kcal/mole, a number of maximum cycles to limit the search directions of fifteen times the number of atoms, and a Polak-Ribiere algorithm (a gradient method using one-dimensional searches) were used in the molecular modelling process. Bond dipole was used to calculate all nonbonded electrostatic interactions. In the MM+ calculations potential energy depends on bond lengths, bond angles, torsion angles, and nonbonded interactions (van der Waals forces, electrostatic interactions, and hydrogen bonds) [50,51].

### Conformational analysis

In order to find the most stable conformation even for titanocenes or cyclodextrins, a conformational analysis by using *Conformational Search* functionality (HyperChem 5.11) was performed. In titanocene structures only some side chains have flexible bonds. On the other hand, the flexible bonds in cyclodextrins were only those corresponding to the hydroxymethyl from C^5^ position of glucopyranose unit; the flexible rings were all glucopyranose rings and the corresponding macrocyclic ring. The following conditions were set up for conformational search: variation of the flexible torsion angles ±60º Ã· ±180º, energy criterion for acceptance of the conformation 4 kcal/mole above minimum, all conformations with atomic distances lower than 0.5 Å, and differences between torsion angles lower than 15º were not considered as well as conformations with energy differences lower than 0.05 kcal/mole (duplicates); the maximum number of optimization and iterative calculations was 1000 and maximum 20 conformations were retained. The hydrogen atoms were neglected.

### Geometry optimization of titanocene / cyclodextrin supramolecular systems

The geometry optimization of titanocene (the most stable conformations) / cyclodextrin (α-, β-, and γ-cyclodextrin) complexes was realized by using the molecular mechanics interactions of the host-guest molecules in vacuum. The titanocene and cyclodextrin structures in minimal energy conformations were set up at distances of ~8Å between the gravity centres of the host-guest molecules, and the titanocene structure was oriented with the hydrophobic side chain in front of the primary (A) or secondary (B) face of cyclodextrin (the principal axis corresponding to the biocompound or side chain moiety was perpendicular to the A or B plan of cyclodextrin). The complex was modeled in absence of water molecules by using the same MM+ functionality and the interaction was stopped when the RMS gradient was lower than 0.005 kcal/mole. The titanocene-cyclodextrin interaction energy was evaluated as the difference between the overall energies of these two molecules and the energy of the complex.

### Structural parameters, correlations, and QSARs

The main molecular descriptors of titanocenes were evaluated by using *QSAR Properties* functionality from the HyperChem 5.11 package. The following descriptors were calculated and were used as structural parameters for obtaining correlations with titanocene-cyclodextrin interaction energy or quantitative structure-activity relationships (QSARs): van der Waals molecular surface (*S*^*vdW*^, Å^2^; van der Waals surface area was carried out by an approximate developed by Still and co-workers [52,53]), van der Waals molecular volume (*V*^*vdW*^, Å^3^; the grid method described by Bodor et al. [54] was used for van der Waals volume calculation. The *QSAR Properties* functionality uses the atomic radii of Gavezotti [55] for this method), hydration energy (*E*_*hydr*_, kcal/mole; the method of calculation is developed by Ooi et al. [56]. The calculation is based on exposed surface area, and employs the surface area as computed by the approximate method, weighted by atom type), logarithm of the octanol/water partition coefficient (log*P*; it was calculated by means of atomic contributions [57,58]. Metal atoms were excepted from calculation; this approximation did not significantly affect the final results due to the “hidden” positions of these metal atoms), refractivity (*Rf*, Å^3^; the refractivity is estimated by the same method as log*P*, presented by Ghose and Crippen [59]), and polarizability (*Pol*, Å^3^; this parameter was estimated according to an additive method of Miller [60], where the different increments are associated with different atom types).

Monolinear correlations (Eqs. 8a and 8b) in theoretical titanocene-cyclodextrin interaction experiments and QSARs were evaluated by using interaction energy between titanocene and cyclodextrin molecules (*E*_*int.*_) or the above mentioned p*A* cytotoxic activity and structural parameters (*P*).

(8a) $$\begin{array}{ccc} \;E_{int.,i} & \;= & a_{0}+b\cdot P_{i}\cdot \end{array}$$

(8b) $$\begin{array}{ccc} \;pA_{i} & \;= & a_{0}+b\cdot P_{i}\cdot \end{array}$$

## Competing interests

The authors declare that they have no competing interests.

## Authors’ contributions

NGH and DIH carried out all theoretical experiments and prepared the final manuscript. AR and ZG help to discuss the theoretical results. All authors read and approved the final manuscript.

## Supplementary Material

Additional file 1 **Molecular modelling and complex optimization of titanocene **/ **cyclodextrin systems.** Description: In this additional file the molecular modelling of titanocenes (A1), the quantitative structure-activity relationships (QSARs), which contain correlations between experimental and predicted activities, as well as the cross-validation data (leave-half-out method) for QSARs (A2), and complex optimization of titanocene/cyclodextrin complexes (starting positions and the most stable titanocene / cyclodextrin supramolecular systems; variation of the titanocene / cyclodextrin interaction energy in the complexation process; titanocene / cyclodextrin interaction energies correlations) (A3) are presented. These data supports all considerations presented in the manuscript. Click here for file

Additional file 2 **Titanocene **/ **cyclodextrin interaction energies. **Description: In this additional file the interaction energy *versus *the number of cycles data from MM+ optimization experiments for titanocene / cyclodextrin complexation process is presented; this supports all considerations presented in the manuscript. Click here for file
