# Supplementary material for: Titanocene / cyclodextrin supramolecular systems: a theoretical approach
Source: Chem Cent J. 2012 Nov 5;6:129. doi: 10.1186/1752-153X-6-129 (PMC3537657; doi:10.1186/1752-153X-6-129)
Supplement: Additional file 1 — Molecular modelling and complex optimization of titanocene / cyclodextrin systems. Description: In this additional file the molecular modelling of titanocenes (A1), the quantitative structure-activity relationships (QSARs), which contain correlations between experimental and predicted activities, as well as the cross-validation data (leave-half-out method) for QSARs (A2), and complex optimization of titanocene/cyclodextrin complexes (starting positions and the most stable titanocene / cyclodextrin supramolecular systems; variation of the titanocene / cyclodextrin interaction energy in the complexation process; titanocene / cyclodextrin interaction energies correlations) (A3) are presented. These data supports all considerations presented in the manuscript. [file 1752-153X-6-129-S1.doc]

# Additional file 1

# *TITANOCENE / CYCLODEXTRIN SUPRAMOLECULAR SYSTEMS: A THEORETICAL APPROACH*

Adrian Riviş^a^, Nicoleta G. Hădărugă^a^, Zeno Gârban^a^, Daniel I. Hădărugă^b,*^

^a^ Banat’s University of Agricultural Sciences and Veterinary Medicine, Faculty of Food Processing Technology, Chemical Engineering Department, 300645-Timişoara, C. Aradului 119, Romania

^b^ “Politehnica” University of Timişoara, Faculty of Industrial Chemistry and Environmental Engineering, Applied Chemistry and Organic-Natural Compounds Engineering Department, 300006-Timişoara, Victory Sq. 2, Romania

* Corresponding author: Phone: +40-256-404224; Fax: +40-256-403060;

E-mail address: [daniel.hadaruga@chim.upt.ro](mailto:daniel.hadaruga@chim.upt.ro)

**A1. Molecular modeling of titanocenes**


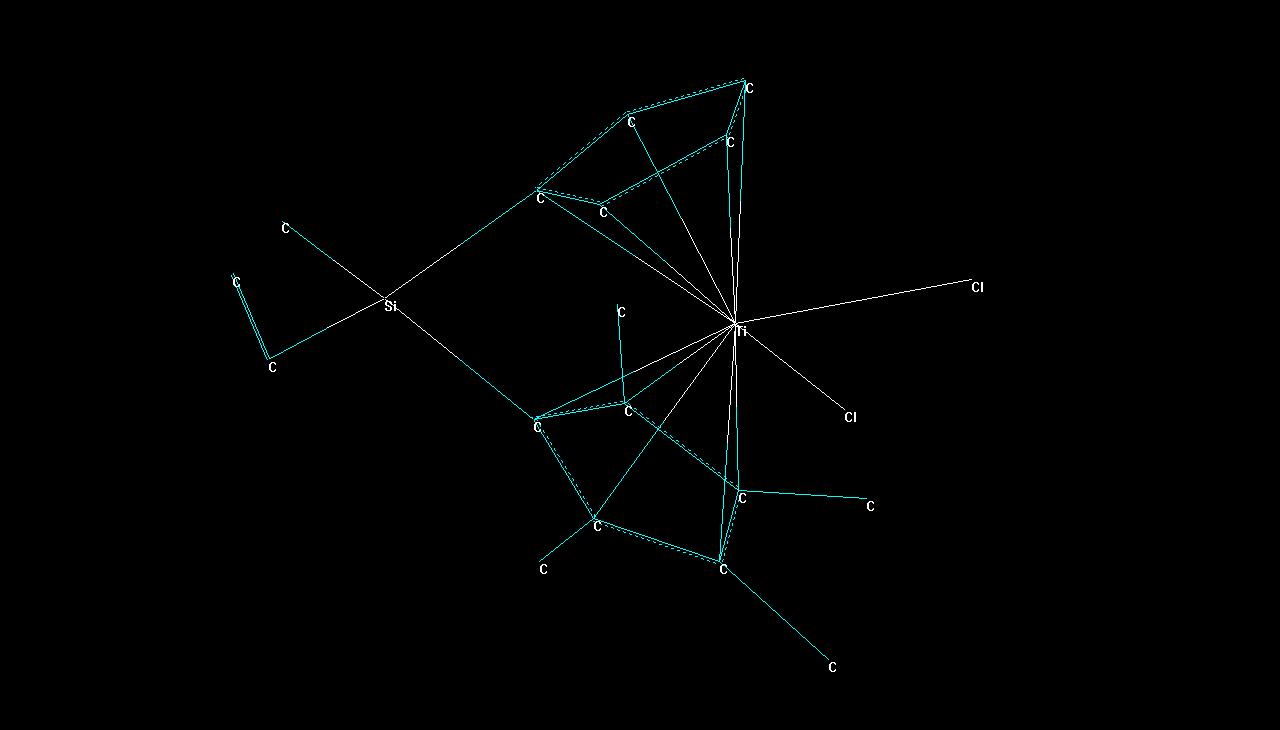


**Figure A1.1.** Minimal energy conformation of titanocene 01TC (MM+; H atoms were neglected)


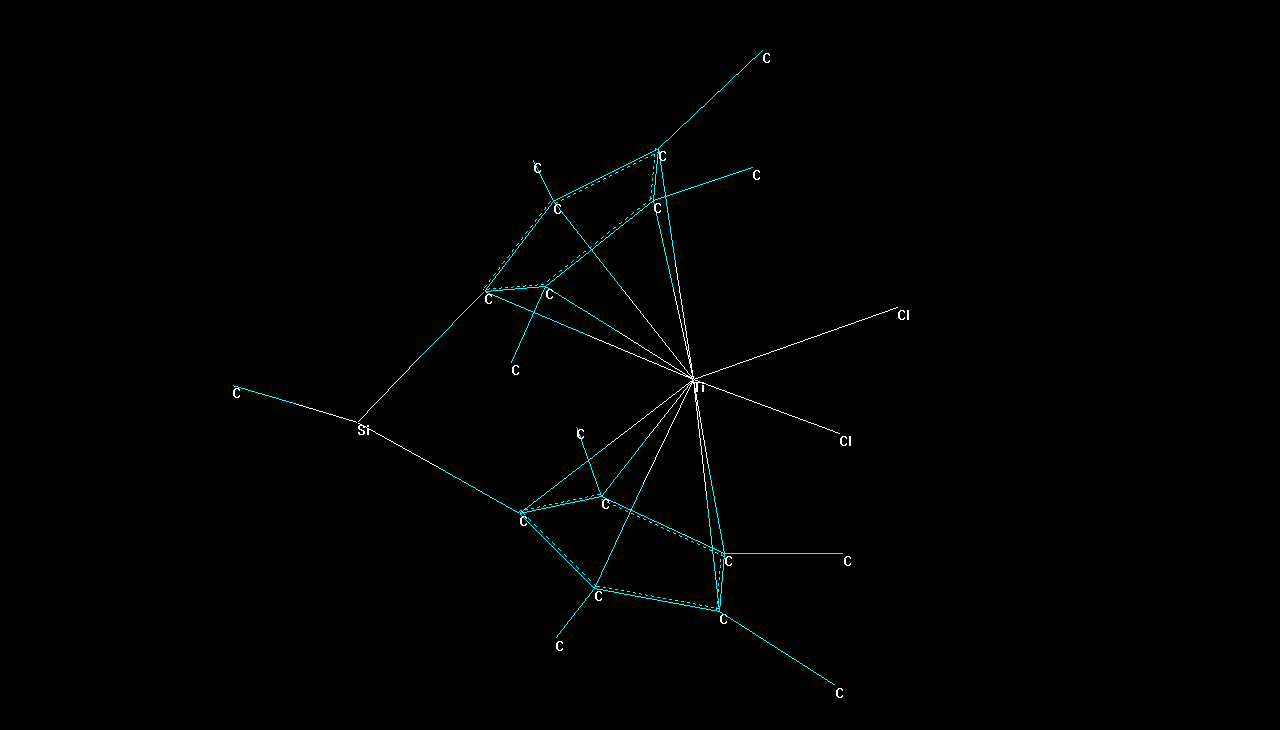


**Figure A1.2.** Minimal energy conformation of titanocene 02TC (MM+; H atoms were neglected)


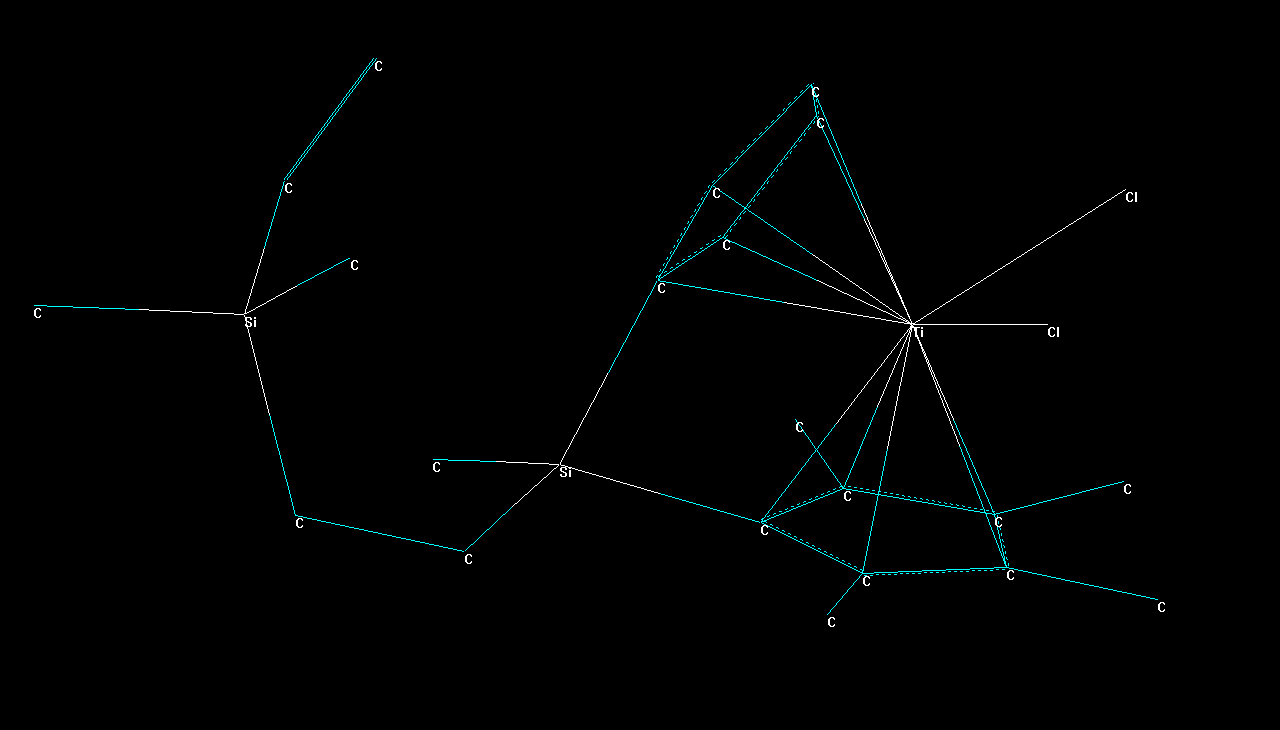


**Figure A1.3.** Minimal energy conformation of titanocene 03TC (MM+; H atoms were neglected)


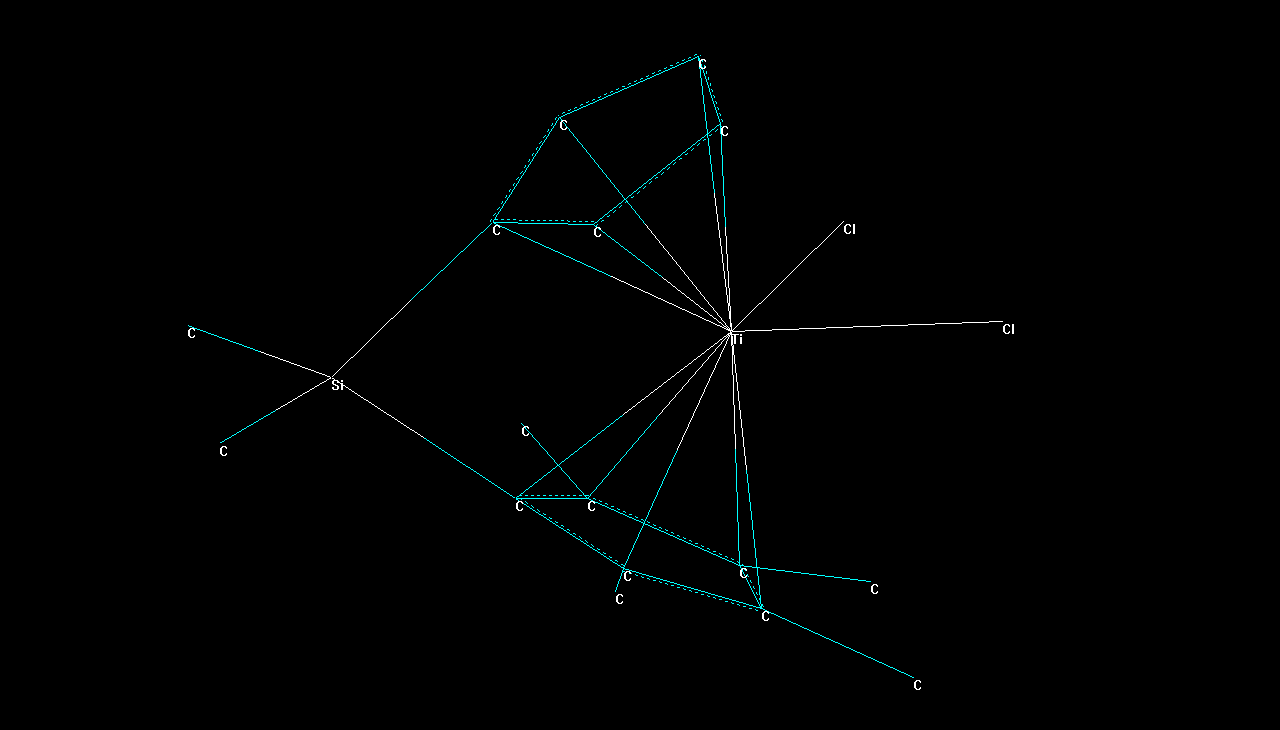


**Figure A1.4.** Minimal energy conformation of titanocene 08TC (MM+; H atoms were neglected)


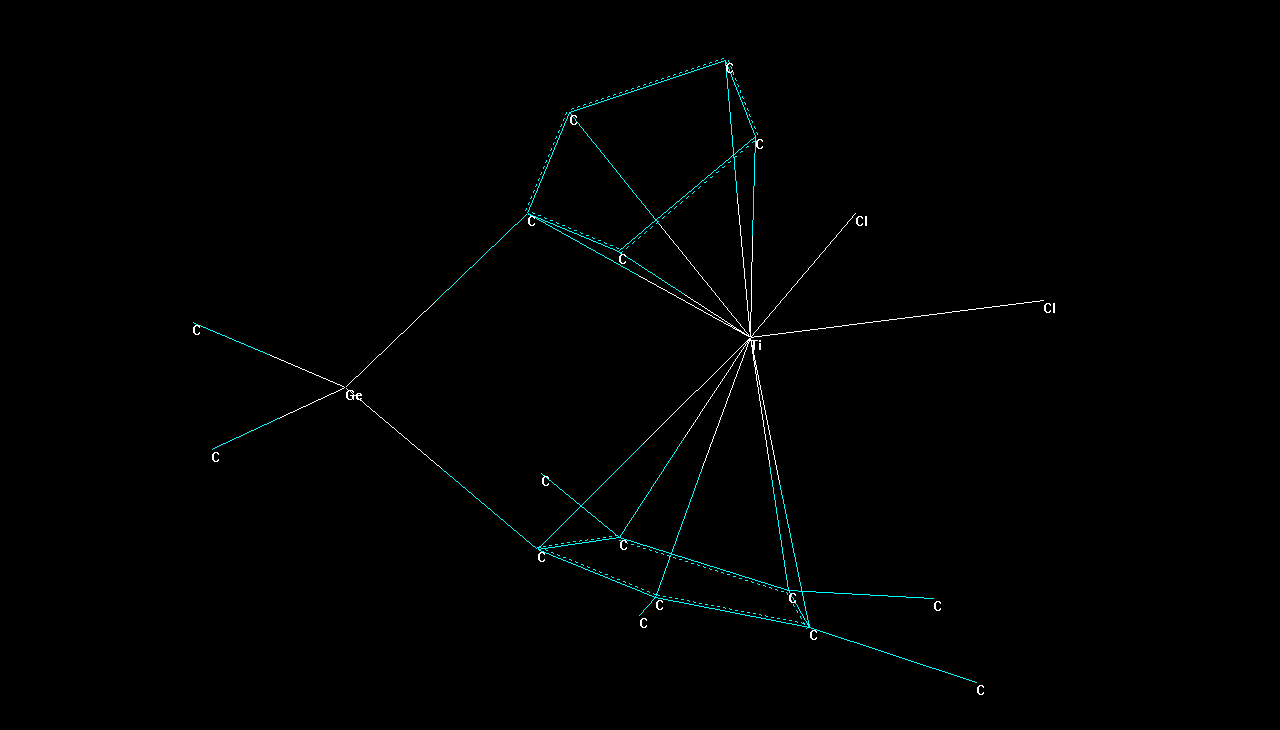


**Figure A1.5.** Minimal energy conformation of titanocene 09TC (MM+; H atoms were neglected)


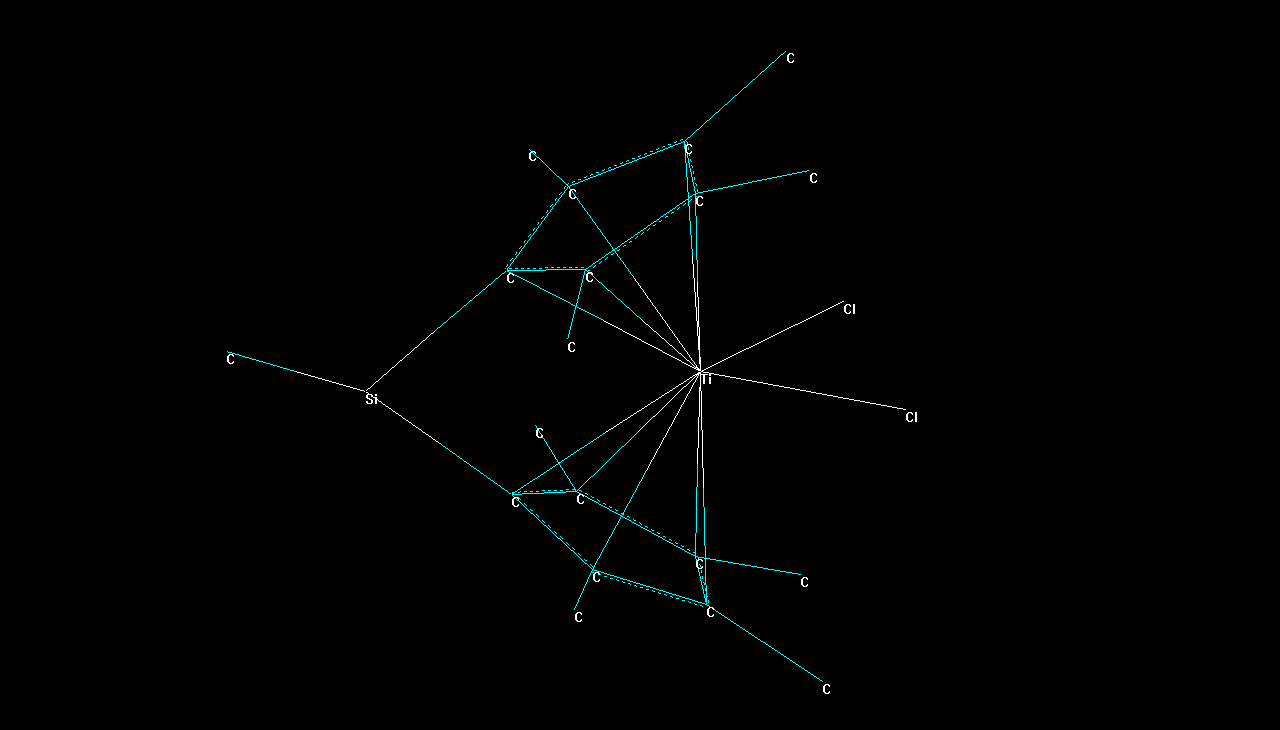


**Figure A1.6.** Minimal energy conformation of titanocene 10TC (MM+; H atoms were neglected)


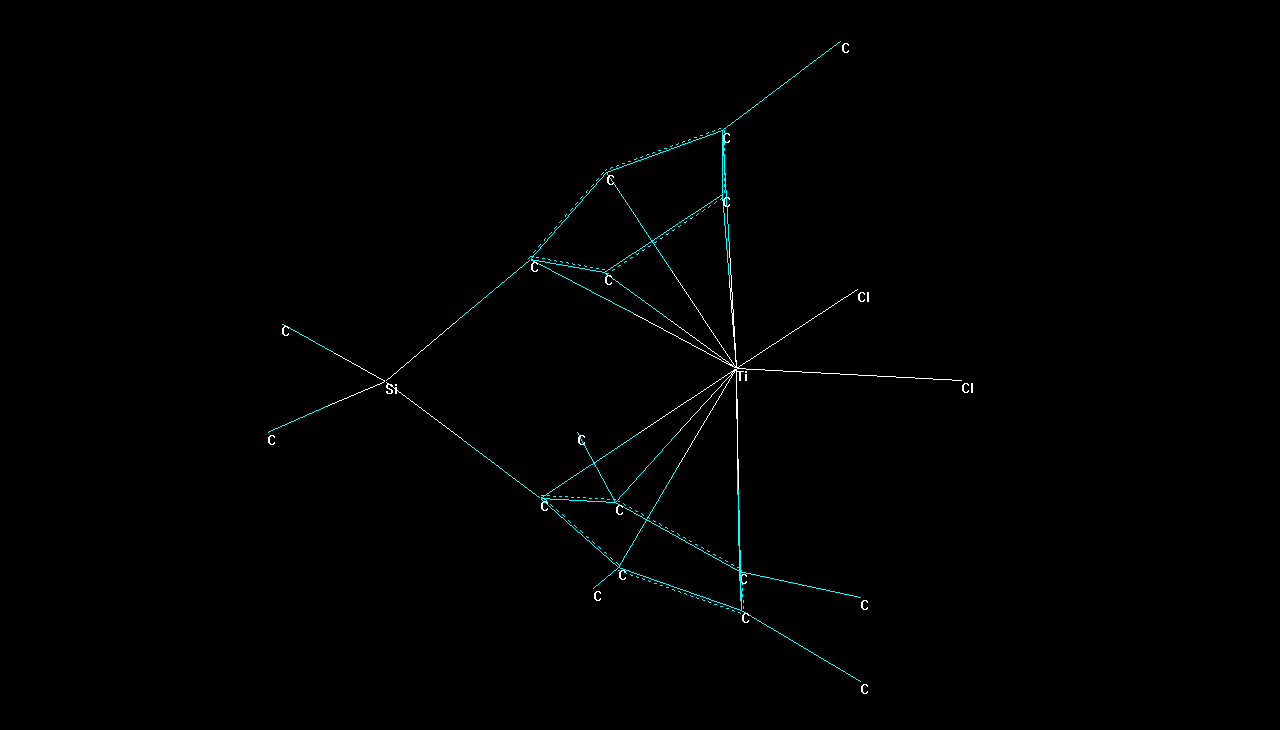


**Figure A1.7.** Minimal energy conformation of titanocene 11TC (MM+; H atoms were neglected)


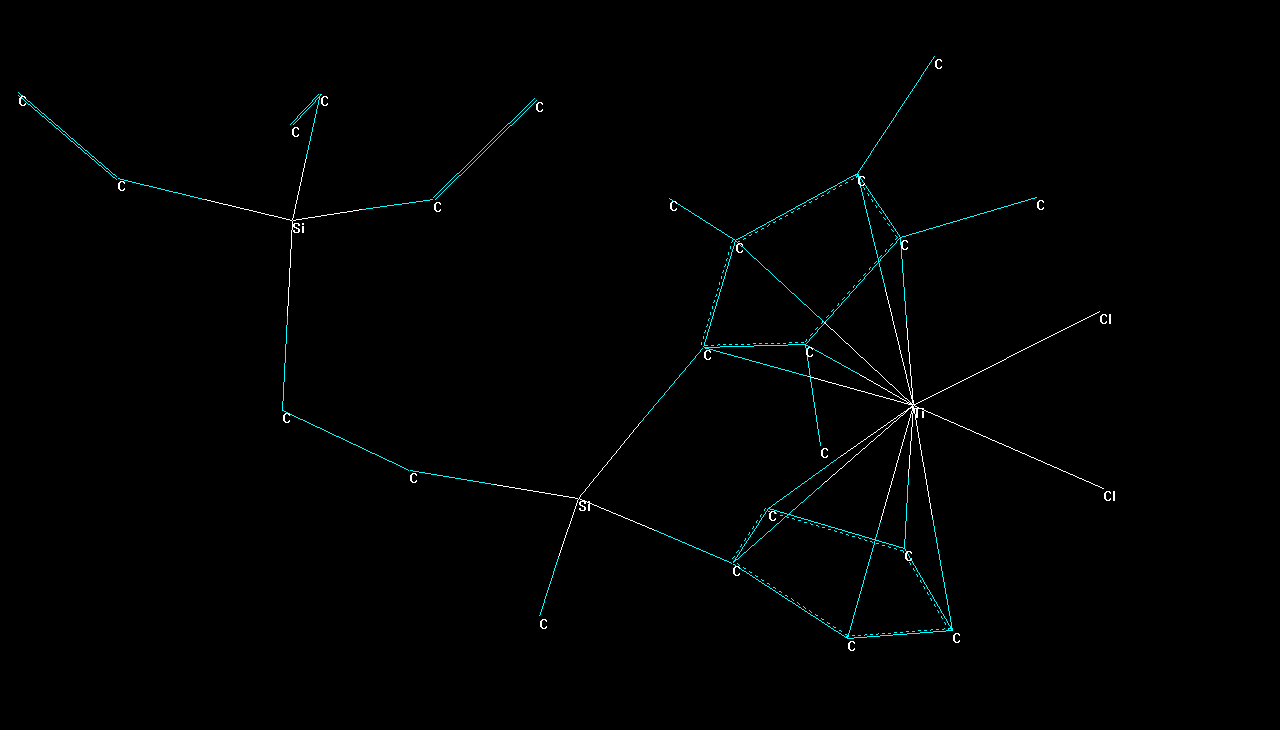


**Figure A1.8.** Minimal energy conformation of titanocene 18TC (MM+; H atoms were neglected)


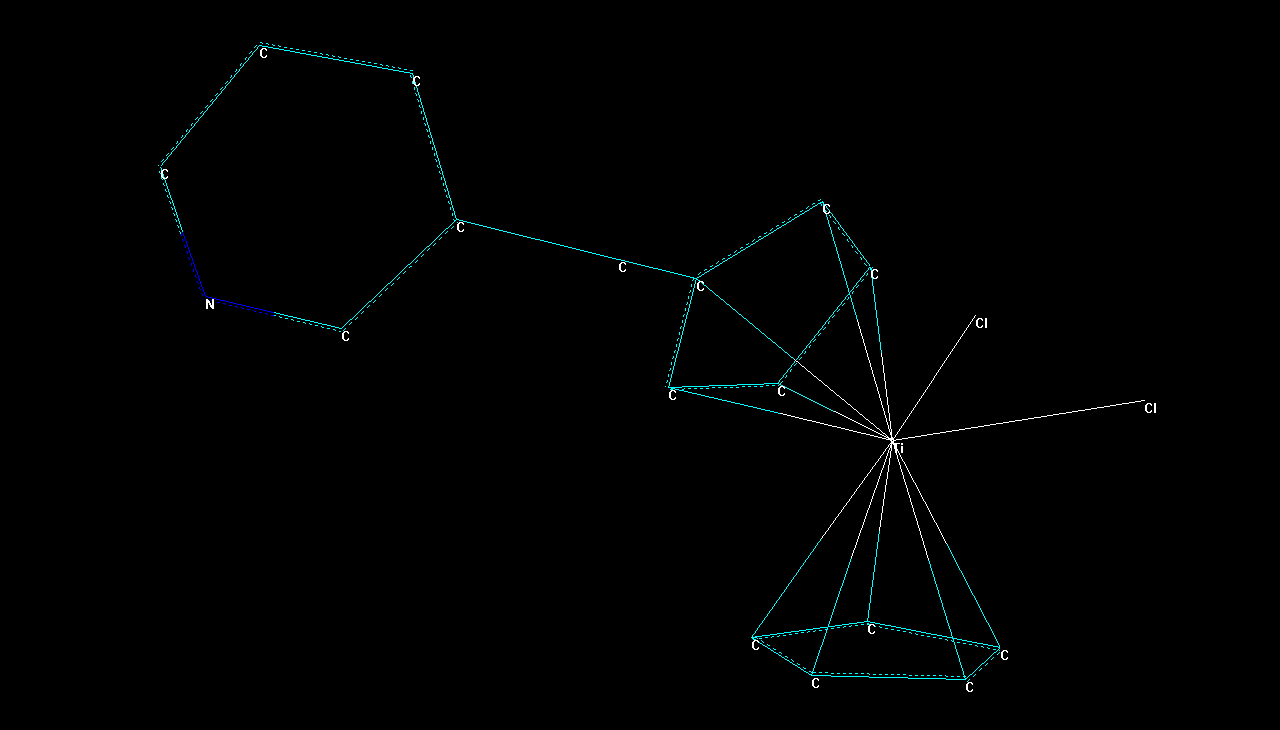


**Figure A1.9.** Minimal energy conformation of titanocene 23TC (MM+; H atoms were neglected)


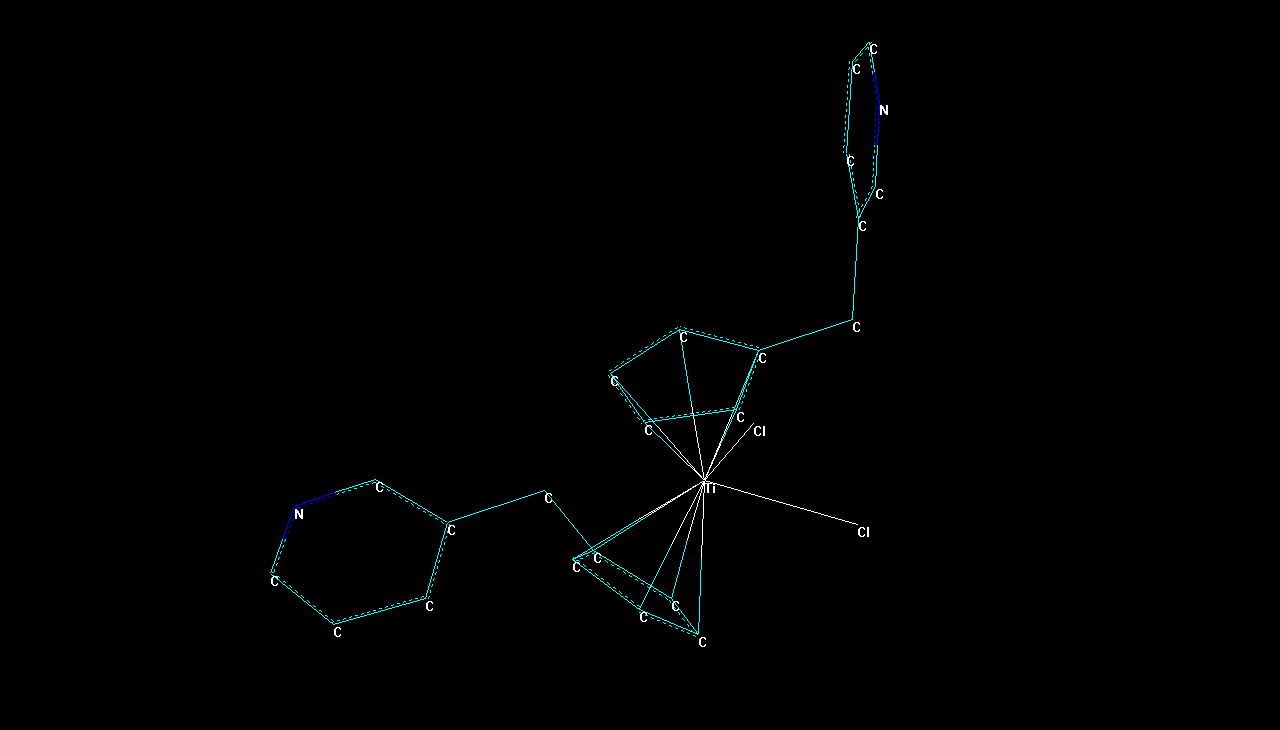


**Figure A1.10.** Minimal energy conformation of titanocene 24TC (MM+; H atoms were neglected)


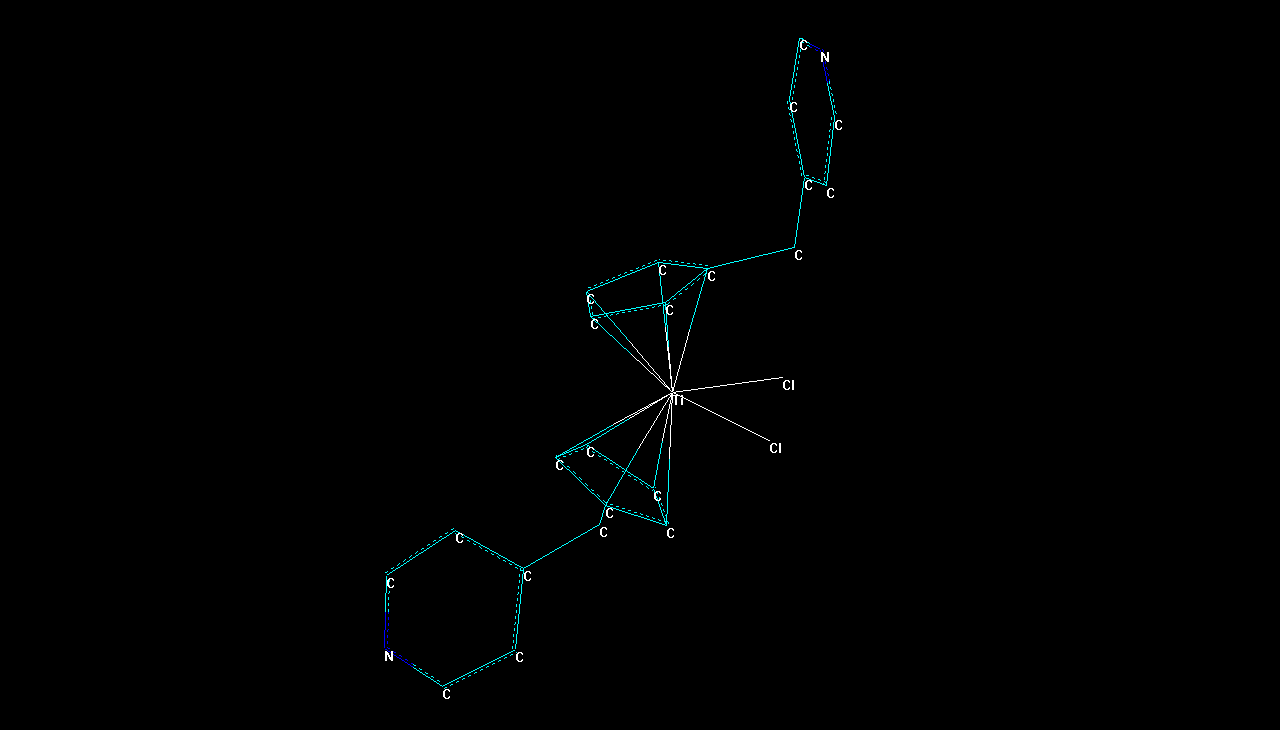


**Figure A1.11.** Minimal energy conformation of titanocene 26TC (MM+; H atoms were neglected)

**A2. Quantitative structure-activity relationships (QSARs)**

***A2.1. Correlations between experimental and predicted activities:***

$pA_{1i(\exp.)}=0.005+0.999\cdot pA_{1i(pred.)}$ [Eq. A2.1a]

*n* = 11; *r* = 0.800; *F* = 16

$pA_{1i}=3.40+0.69\cdot\log P_{i}$ [Eq. A2.1b]

*n* = 11; *r* = 0.80; *F* = 16; *q^2^_cv_* = 0.75

$pA_{2i(\exp.)}=-0.021+1.007\cdot pA_{2i(pred.)}$ [Eq. A2.2a]

*n* = 11; *r* = 0.700; *F* = 5.5

$pA_{2i}=3.54+0.69\cdot\log P_{i}$ [Eq. A2.2b]

*n* = 8; *r* = 0.70; *F* = 5.5; *q^2^_cv_* = 0.88

$pA_{3i(\exp.)}=0.103+0.970\cdot pA_{3i(pred.)}$ [Eq. A2.3a]

*n* = 11; *r* = 0.735; *F* = 5.9

$pA_{3i}=3.63+0.39\cdot\log P_{i}$ [Eq. A2.3b]

*n* = 7; *r* = 0.74; *F* = 5.9; *q^2^_cv_* = 0.80

$pA_{2i,Rf(\exp.)}=-0.007+1.001\cdot pA_{2i,Rf(pred.)}$ [Eq. A2.4a]

*n* = 11; *r* = 0.798; *F* = 10.5

$pA_{2i}=5.27-0.014\cdot Rf_{i}$ [Eq. A2.4b]

*n* = 11; *r* = 0.80; *F* = 10.5; *q^2^_cv_* = 0.75

***A.2.2. Cross-validation data (Leave-half-out ODD and EVEN method) for QSARs:***

**Table A2.1.** Cross-validation data (ODD and EVEN) for cytotoxic activity of titanocenes against HeLa cell line (cross-validation predicted p*A_1,cv_* – Eqs. A2.5, and Δp*A_1_* = p*A_1_* *_(exp.)_* – p*A_1,cv_* *_(pred.)_* values; Hydrophobicity – log*P* as parameter)

$pA_{1i,ODD}=3.33\left(\pm0.26\right)+0.86\left(\pm0.27\right)\cdot\log P_{i,ODD}$ [Eq. A2.5a]

*n* = 6; *r* = 0.849; *F* = 10.3

$pA_{1i,EVEN}=3.53\left(\pm0.10\right)+0.46\left(\pm0.11\right)\cdot\log P_{i,EVEN}$ [Eq. A2.5b]

*n* = 5; *r* = 0.929; *F* = 18.9

| **No** | **Code** | **log*P*** | **p*A_1_* *_(exp.)_*** | **p*A_1,cv_* *_(pred.)_*** | **Δp*A_1_*** |
| --- | --- | --- | --- | --- | --- |
| 1 | 01TC | 0.86 | 4.10 | 4.068 | 0.032 |
| 2 | 02TC | 0.8 | 3.96 | 3.895 | 0.065 |
| 3 | 03TC | 0.36 | 3.72 | 3.637 | 0.083 |
| 4 | 08TC | 0.73 | 3.87 | 3.863 | 0.007 |
| 5 | 09TC | 1.06 | 3.81 | 4.241 | -0.431 |
| 6 | 10TC | 0.8 | 3.96 | 3.895 | 0.065 |
| 7 | 11TC | 0.81 | 3.93 | 4.025 | -0.095 |
| 8 | 18TC | 0.62 | 3.70 | 3.813 | -0.113 |
| 9 | 23TC | 0.51 | 3.94 | 3.767 | 0.173 |
| 10 | 24TC | 1.63 | 4.25 | 4.274 | -0.024 |
| 11 | 26TC | 1.63 | 4.97 | 4.732 | 0.238 |

**Table A2.2.** Cross-validation data (ODD and EVEN) for cytotoxic activity of titanocenes against K562 cell line (cross-validation predicted p*A_2,cv_* – Eqs. A2.6, and Δp*A_2_* = p*A_2_* *_(exp.)_* – p*A_2,cv_* *_(pred.)_* values; Hydrophobicity – log*P* as parameter)

$pA_{2i,ODD}=3.64\left(\pm0.14\right)+0.53\left(\pm0.17\right)\cdot\log P_{i,ODD}$ [Eq. A2.6a]

*n* = 4; *r* = 0.910; *F* = 9.6

$pA_{2i,EVEN}=1.95\left(\pm0.48\right)+2.89\left(\pm0.65\right)\cdot\log P_{i,EVEN}$ [Eq. A2.6b]

*n* = 4; *r* = 0.953; *F* = 19.9

| **No** | **Code** | **log*P*** | **p*A_2_* *_(exp.)_*** | **p*A_2,cv_* *_(pred.)_*** | **Δp*A_2_*** |
| --- | --- | --- | --- | --- | --- |
| 1 | 01TC | 0.86 | 4.20 | 4.099 | 0.101 |
| 2 | 02TC | 0.8 | 4.23 | 4.266 | -0.036 |
| 3 | 03TC | 0.36 | 3.81 | 3.835 | -0.025 |
| 4 | 08TC | 0.73 | 4.18 | 4.063 | 0.117 |
| 5 | 09TC | 1.06 | 4.14 | 4.204 | -0.064 |
| 6 | 10TC | 0.8 | 4.23 | 4.266 | -0.036 |
| 7 | 11TC | 0.81 | 4.06 | 4.072 | -0.012 |
| 8 | 18TC | 0.62 | 3.70 | 3.745 | -0.045 |

**Table A2.3.** Cross-validation data (ODD and EVEN) for cytotoxic activity of titanocenes against Fem-x cell line (cross-validation predicted p*A_3,cv_* – Eqs. A2.7, and Δp*A_3_* = p*A_3_* *_(exp.)_* – p*A_3,cv_* *_(pred.)_* values; Hydrophobicity – log*P* as parameter)

$pA_{3i,ODD}=3.58\left(\pm0.13\right)+0.40\left(\pm0.16\right)\cdot\log P_{i,ODD}$ [Eq. A2.7a]

*n* = 4; *r* = 0.865; *F* = 6.0

$pA_{3i,EVEN}=4.91\left(\pm0.10\right)-1.21\left(\pm0.12\right)\cdot\log P_{i,EVEN}$ [Eq. A2.7b]

*n* = 3; *r* = 0.995; *F* = 96.3

| **No** | **Code** | **log*P*** | **p*A_3_* *_(exp.)_*** | **p*A_3,cv_* *_(pred.)_*** | **Δp*A_3_*** |
| --- | --- | --- | --- | --- | --- |
| 1 | 01TC | 0.86 | 3.87 | 3.920 | -0.050 |
| 2 | 02TC | 0.8 | 3.93 | 3.935 | -0.005 |
| 3 | 03TC | 0.36 | 3.7 | 3.722 | -0.022 |
| 4 | 08TC | 0.73 | 4.02 | 4.020 | 0.000 |
| 5 | 09TC | 1.06 | 3.97 | 3.999 | -0.029 |
| 6 | 10TC | 0.8 | 3.94 | 3.935 | 0.005 |
| 7 | 11TC | 0.81 | 4 | 3.900 | 0.100 |

**Table A2.4.** Cross-validation data (ODD and EVEN) for cytotoxic activity of titanocenes against K562 cell line (cross-validation predicted p*A_2,Rf,cv_* – Eqs. A2.8, and Δp*A_2_* = p*A_2_* *_(exp.)_* – p*A_2,Rf,cv_* *_(pred.)_* values; Refractivity – *Rf* (Å^3^) as parameter)

$pA_{2i,ODD}=5.31\left(\pm0.29\right)-0.015\left(\pm0.004\right)\cdot Rf_{i,ODD}$ [Eq. A2.8a]

*n* = 4; *r* = 0.950; *F* = 18.6

$pA_{2i,EVEN}=5.52\left(\pm0.71\right)-0.016\left(\pm0.008\right)\cdot Rf_{i,EVEN}$ [Eq. A2.8b]

*n* = 4; *r* = 0.882; *F* = 4.2

| **No** | **Code** | ***Rf* (Å^3^)** | **p*A_2_* *_(exp.)_*** | **p*A_2,Rf,cv_* *_(pred.)_*** | **Δp*A_2_*** |
| --- | --- | --- | --- | --- | --- |
| 1 | 01TC | 77.7 | 4.20 | 4.130 | 0.070 |
| 2 | 02TC | 87.7 | 4.23 | 4.107 | 0.123 |
| 3 | 03TC | 98.5 | 3.81 | 3.814 | -0.003 |
| 4 | 08TC | 74 | 4.18 | 4.327 | -0.147 |
| 5 | 09TC | 75.5 | 4.14 | 4.163 | -0.023 |
| 6 | 10TC | 87.7 | 4.23 | 4.107 | 0.123 |
| 7 | 11TC | 78.6 | 4.06 | 4.116 | -0.056 |
| 8 | 18TC | 106 | 3.70 | 3.812 | -0.112 |

**A3. Docking of titanocenes in cyclodextrins**

***A3.1. Starting positions and the most stable (minimum energy of the complex) titanocene (bold) / cyclodextrin supramolecular systems:***


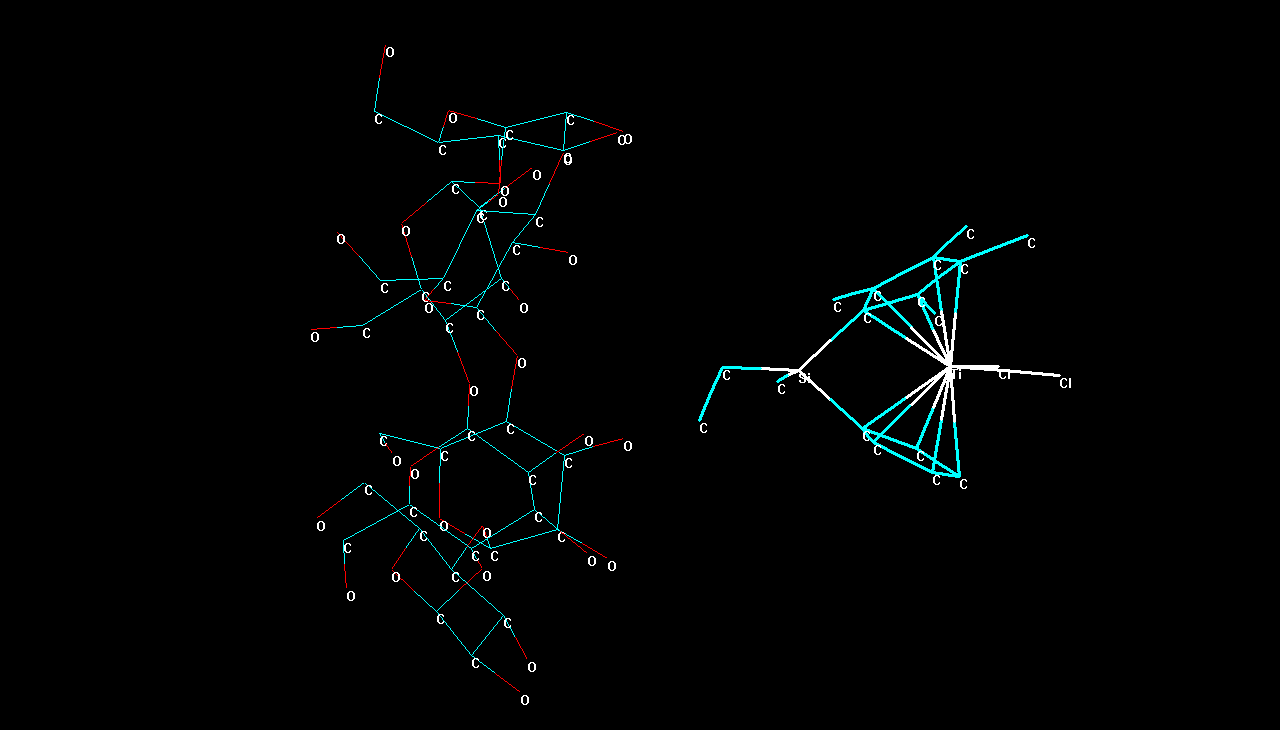


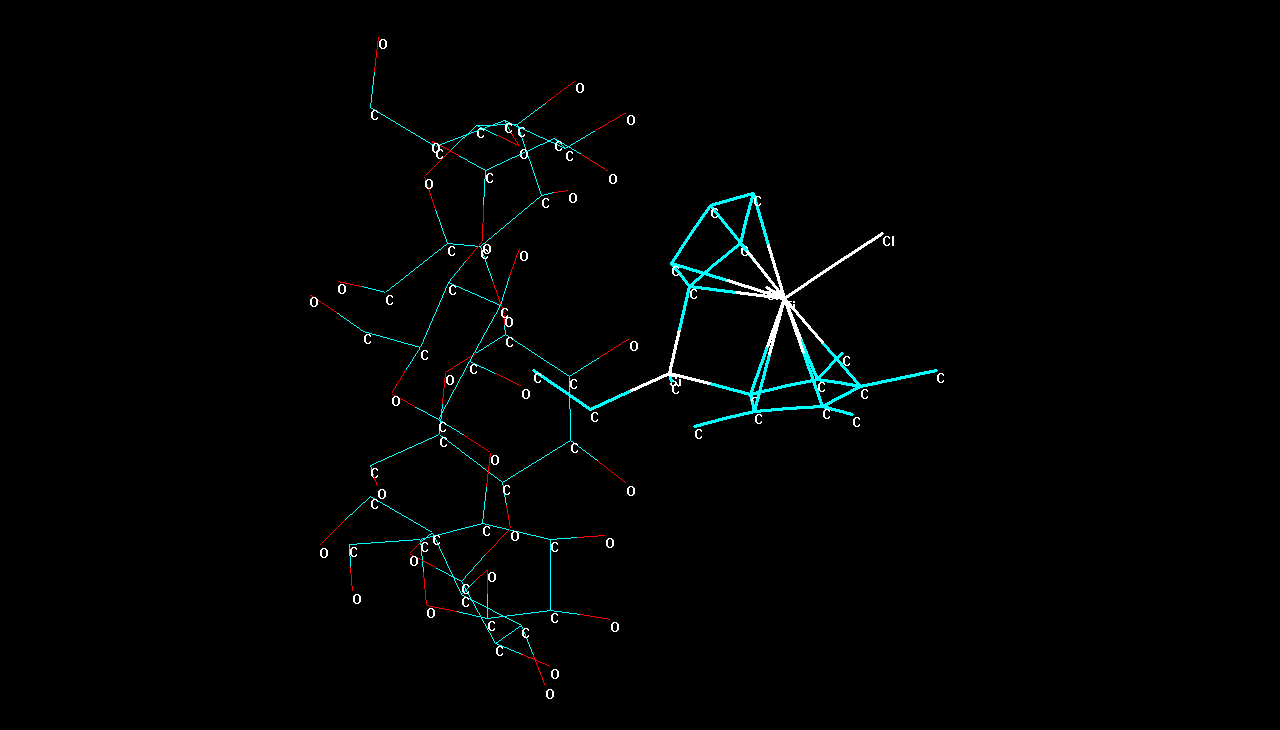


**Figure A3.1.** Starting position (up) and the minimum energy supramolecular system (down) for titanocene (code 01TC) / α-cyclodextrin complex (theoretically modeled by MM+ docking experiments)


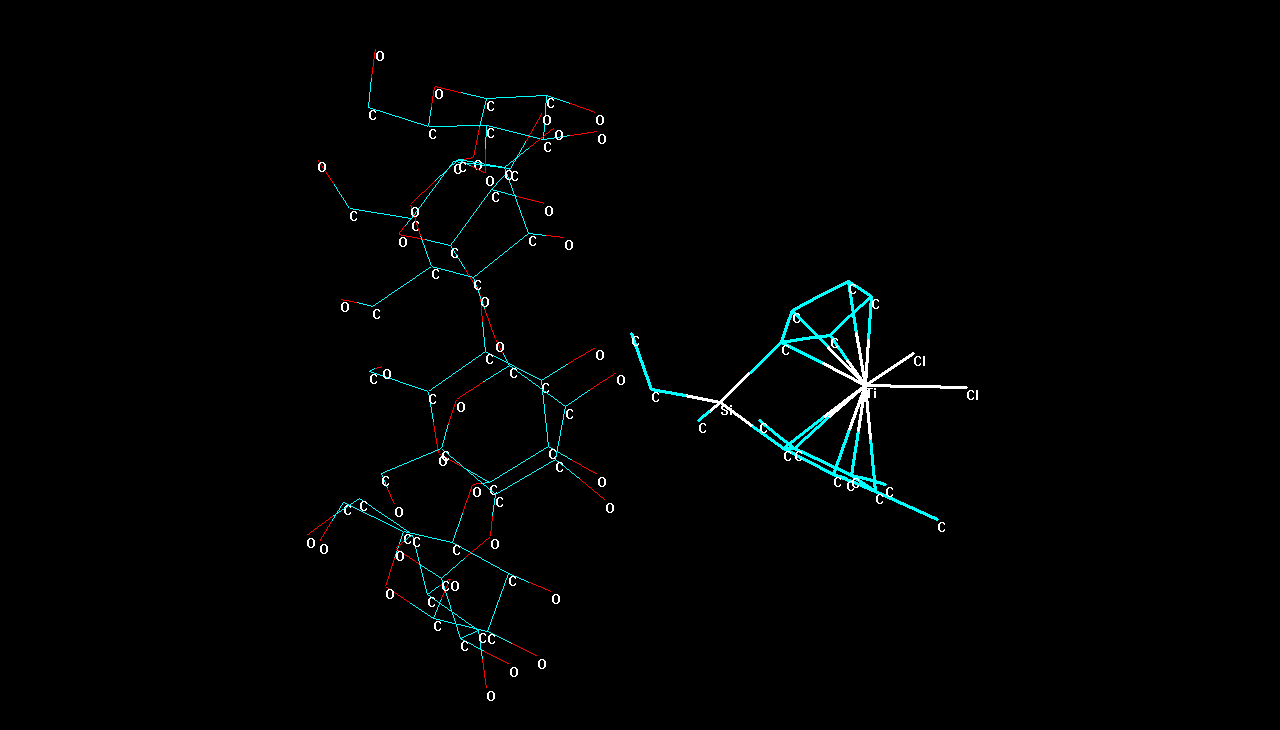


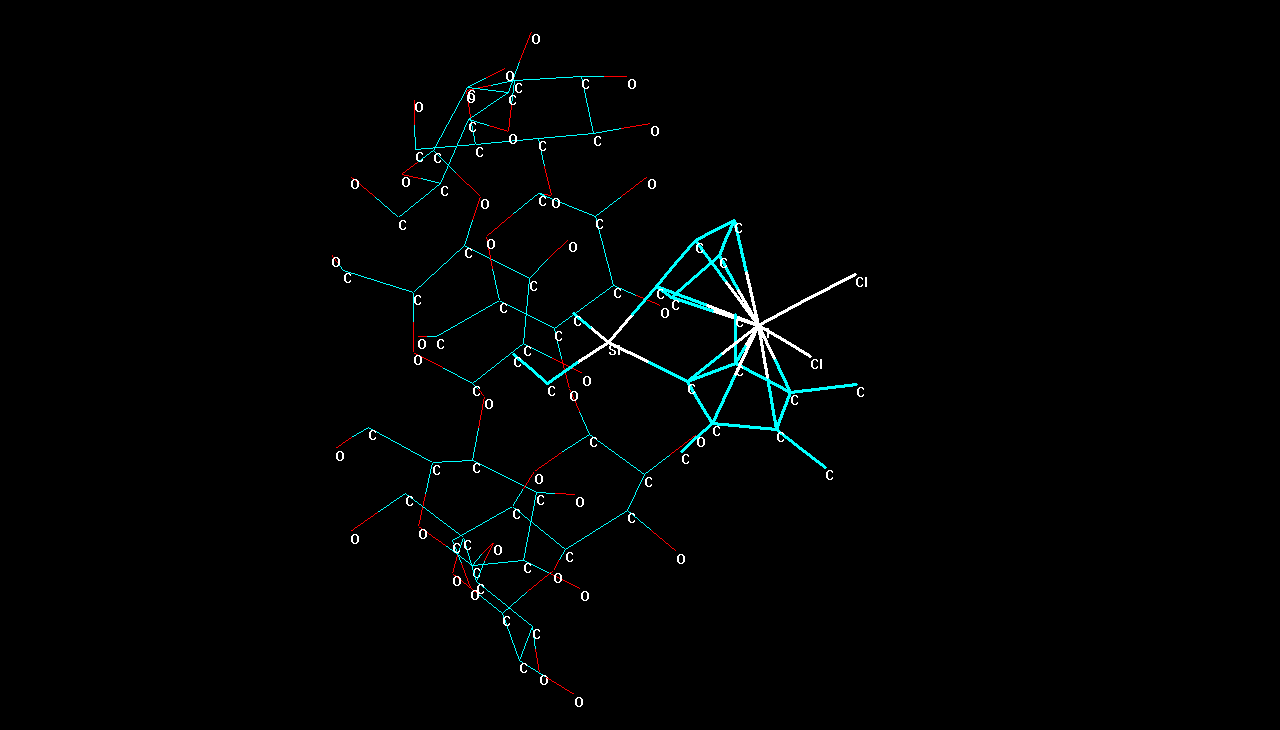


**Figure A3.2.** Starting position (up) and the minimum energy supramolecular system (down) for titanocene (code 01TC) / β-cyclodextrin complex (theoretically modeled by MM+ docking experiments)


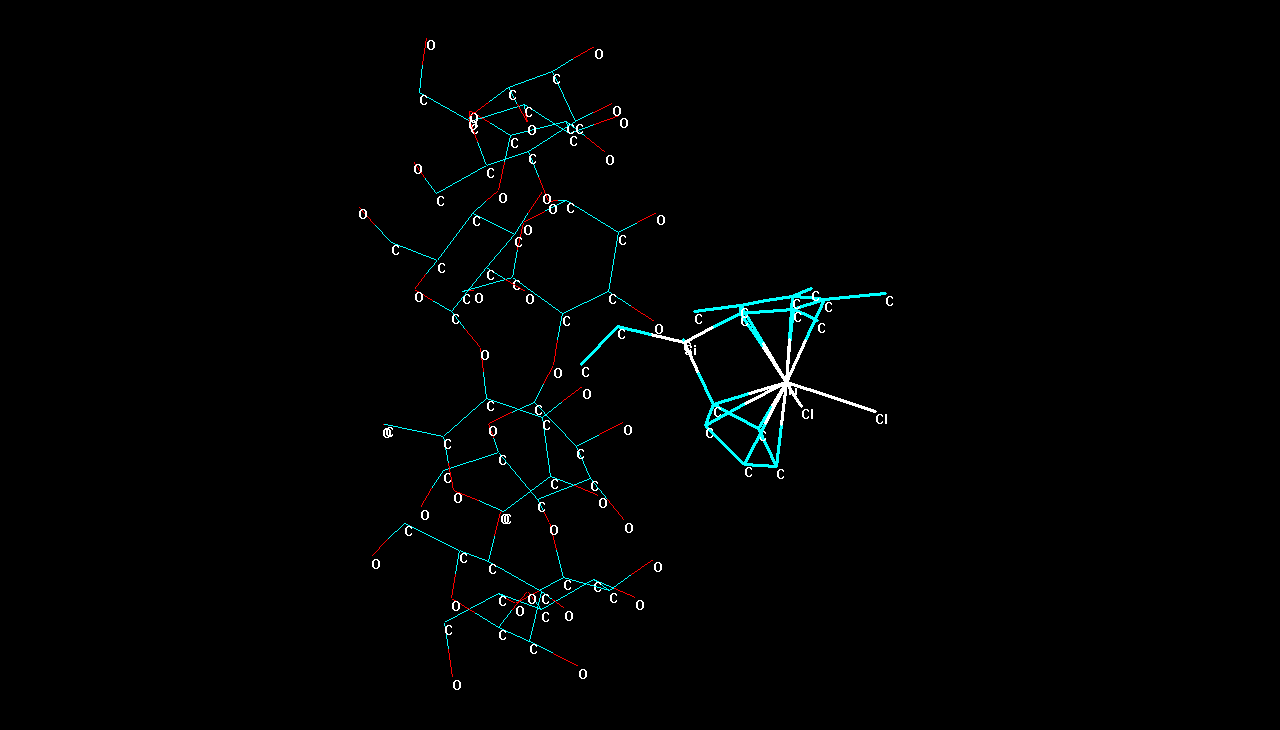


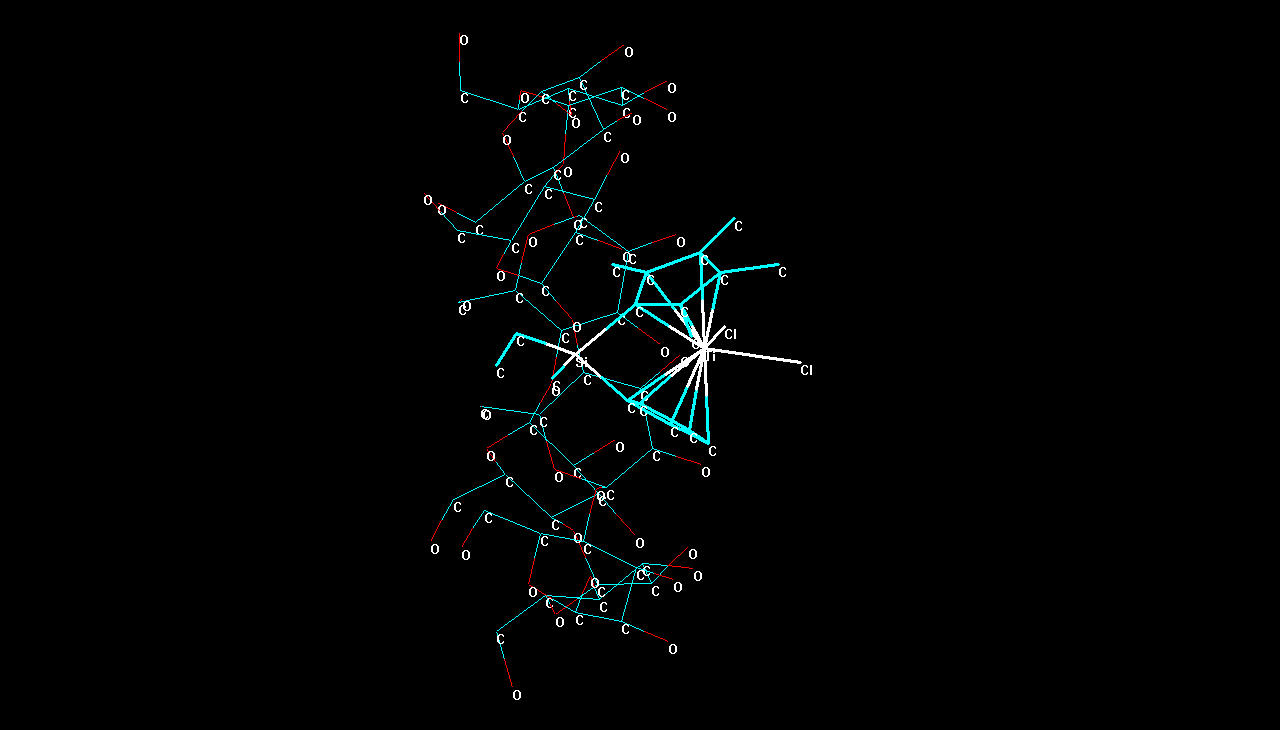


**Figure A3.3.** Starting position (up) and the minimum energy supramolecular system (down) for titanocene (code 01TC) / γ-cyclodextrin complex (theoretically modeled by MM+ docking experiments)


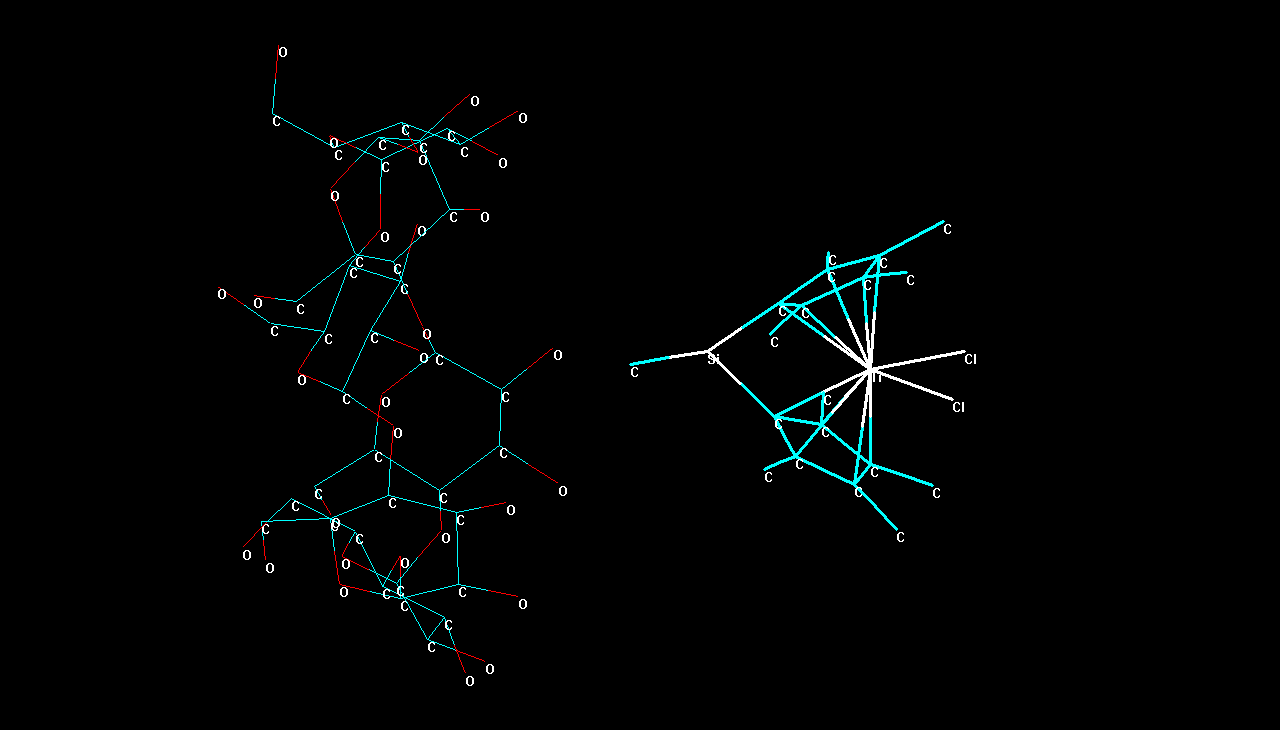


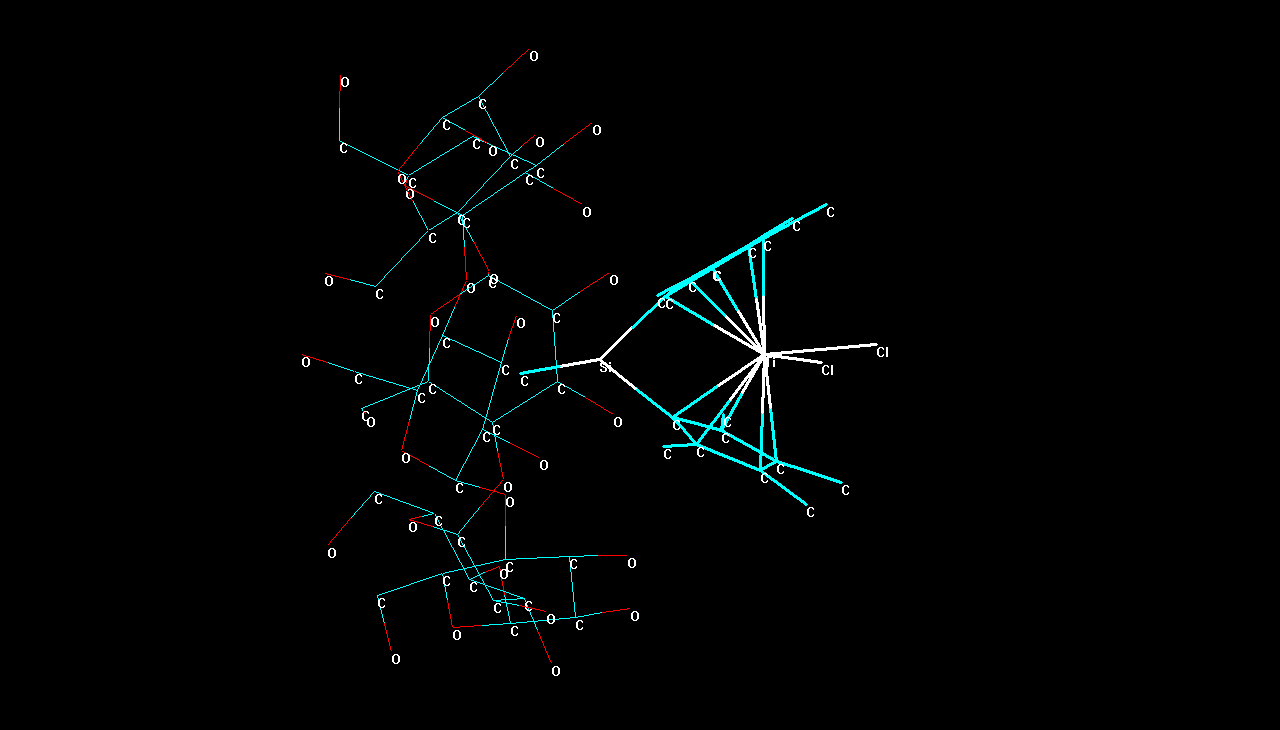


**Figure A3.4.** Starting position (up) and the minimum energy supramolecular system (down) for titanocene (code 02TC) / α-cyclodextrin complex (theoretically modeled by MM+ docking experiments)


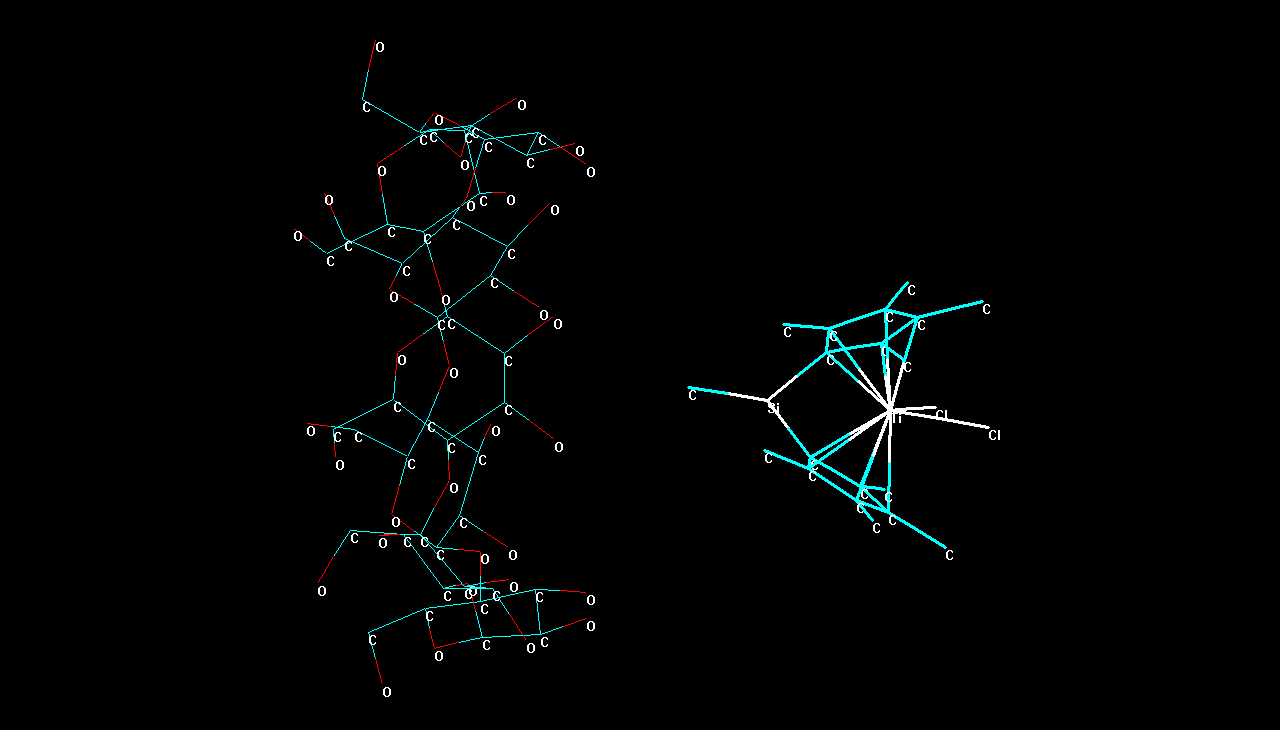


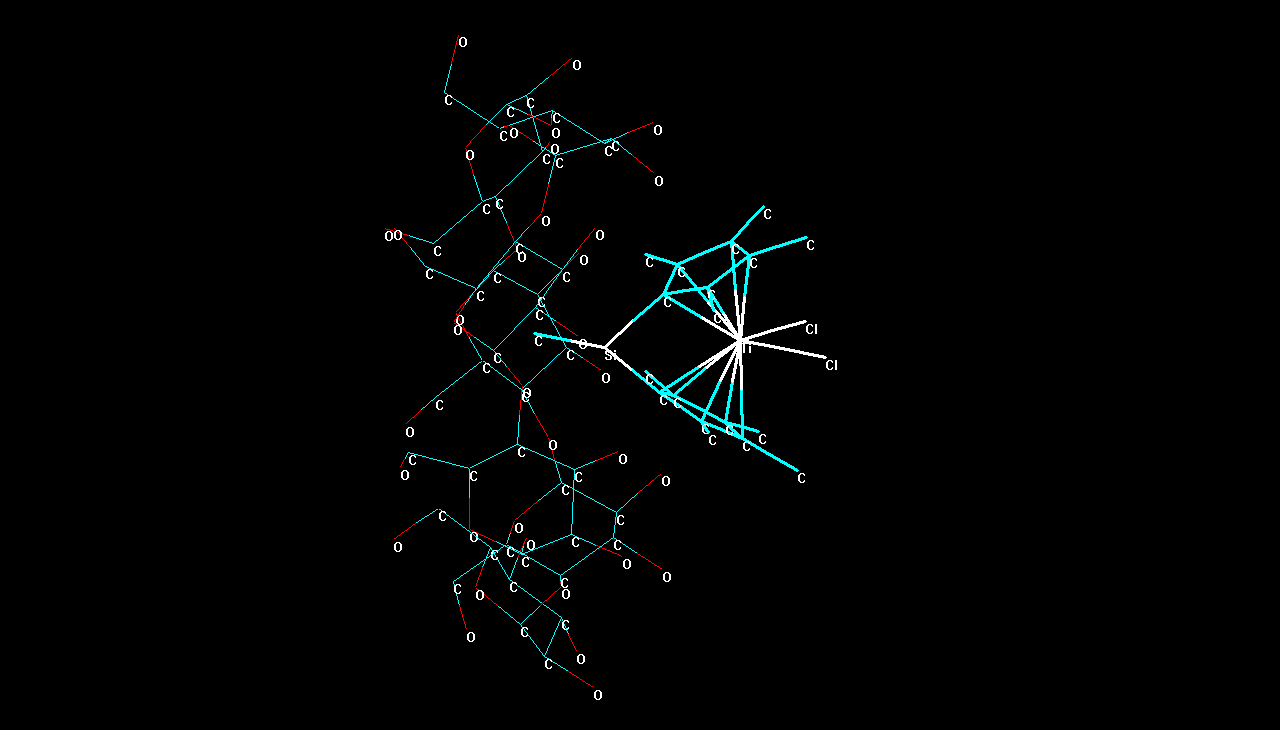


**Figure A3.5.** Starting position (up) and the minimum energy supramolecular system (down) for titanocene (code 02TC) / β-cyclodextrin complex (theoretically modeled by MM+ docking experiments)


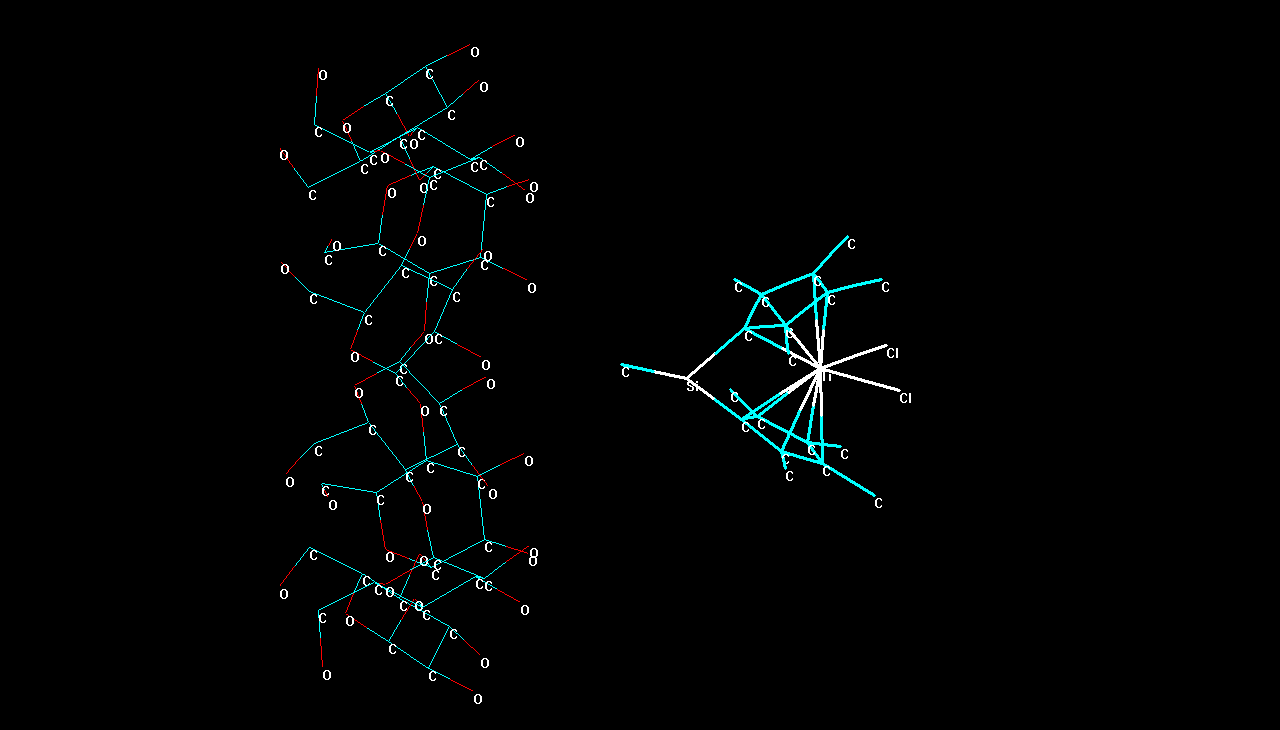


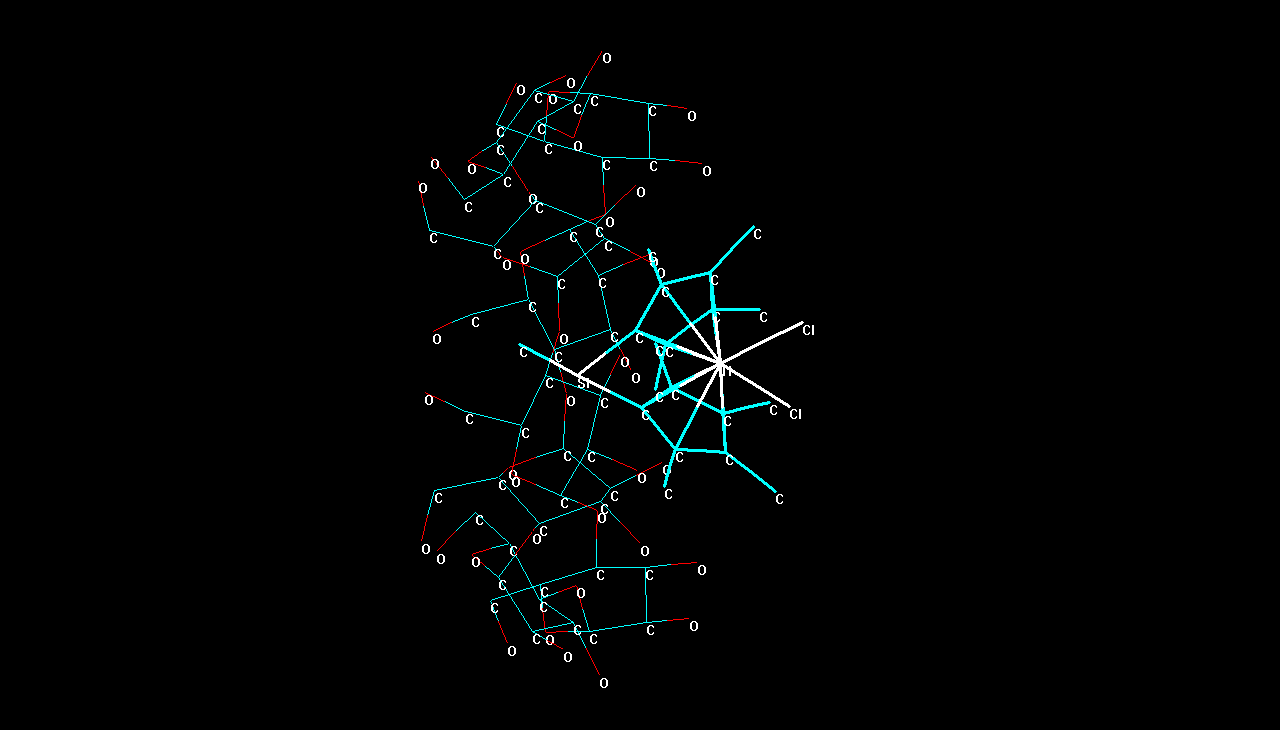


**Figure A3.6.** Starting position (up) and the minimum energy supramolecular system (down) for titanocene (code 02TC) / γ-cyclodextrin complex (theoretically modeled by MM+ docking experiments)


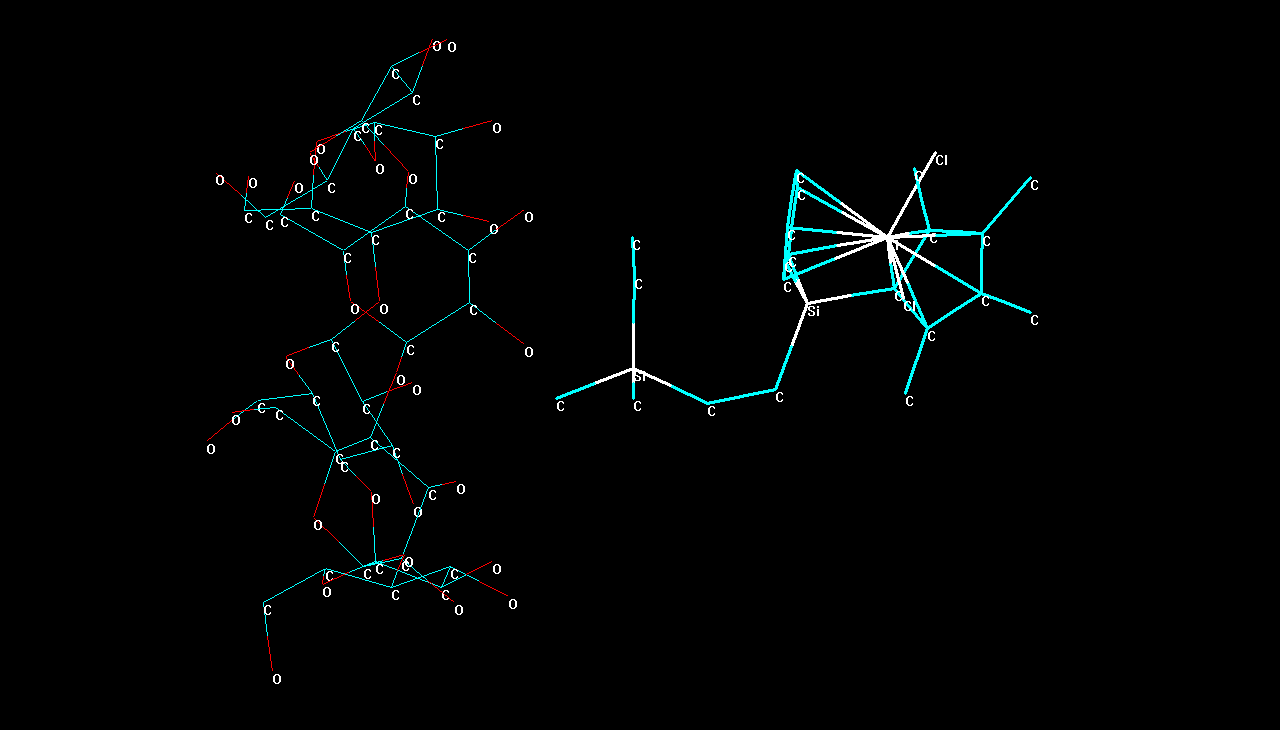


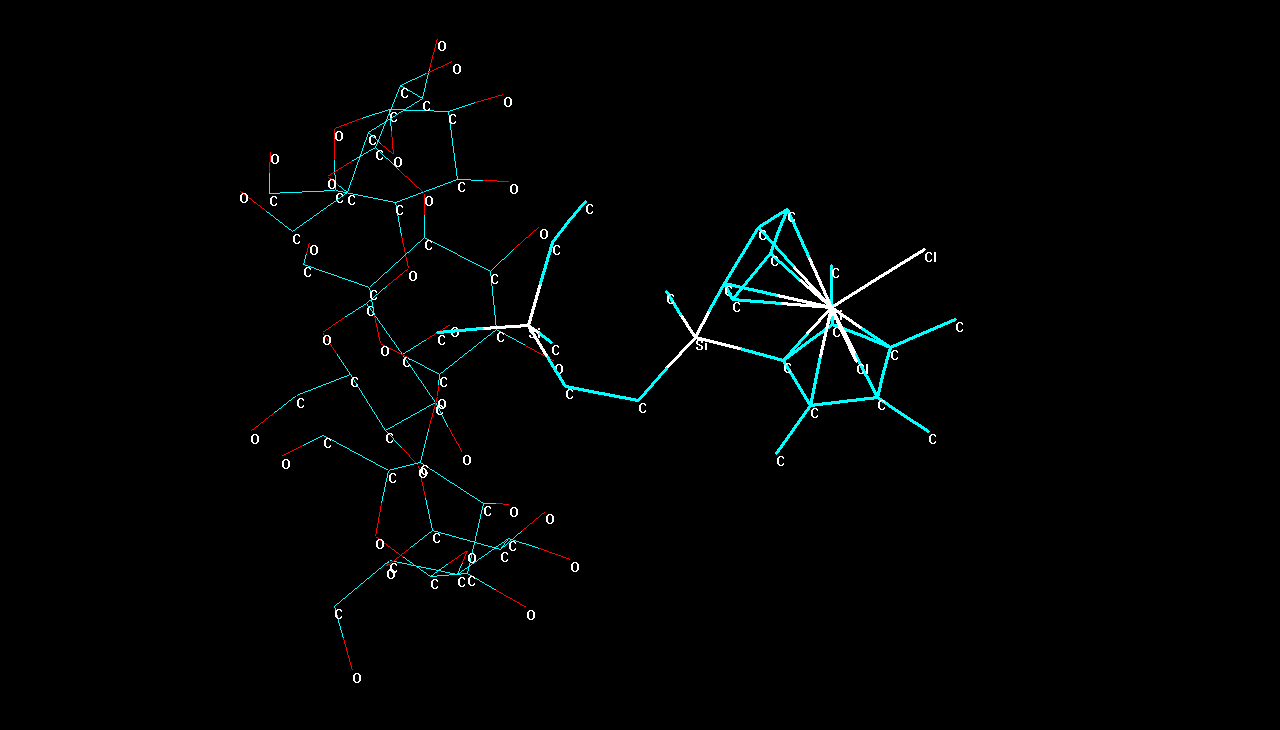


**Figure A3.7.** Starting position (up) and the minimum energy supramolecular system (down) for titanocene (code 03TC) / α-cyclodextrin complex (theoretically modeled by MM+ docking experiments)


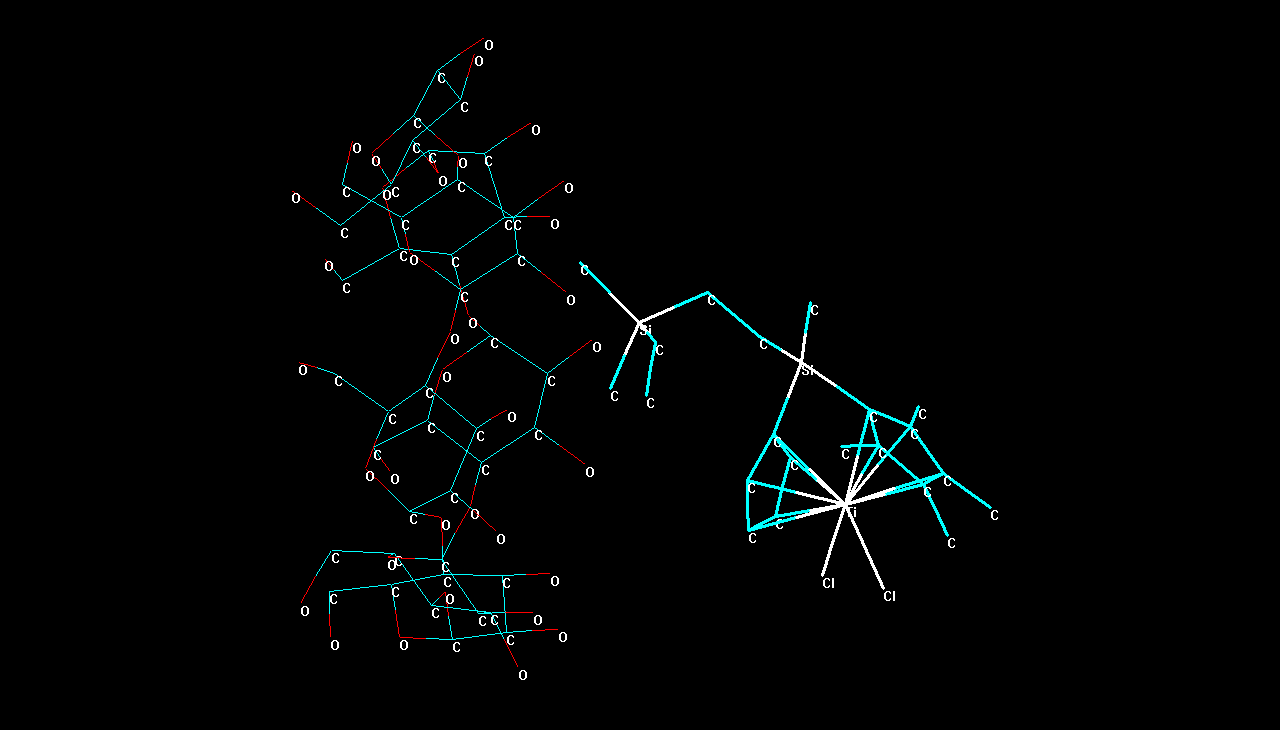


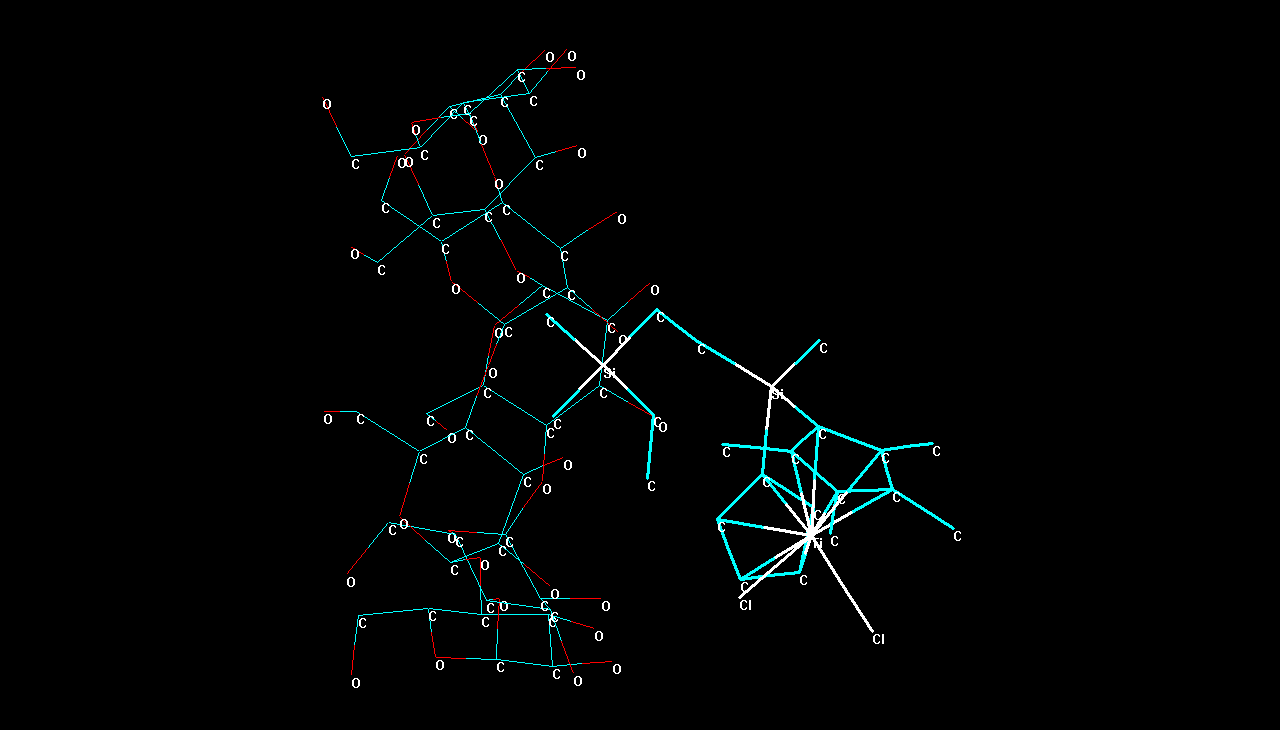


**Figure A3.8.** Starting position (up) and the minimum energy supramolecular system (down) for titanocene (code 03TC) / β-cyclodextrin complex (theoretically modeled by MM+ docking experiments)


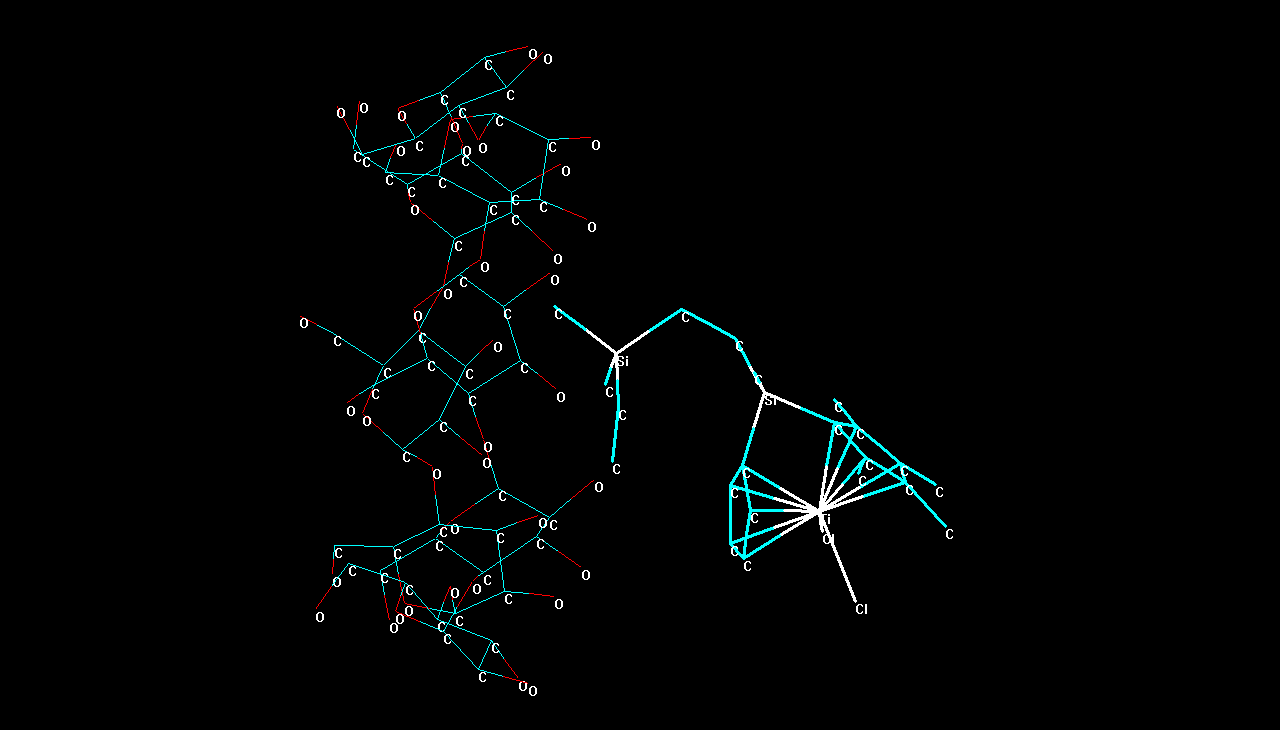


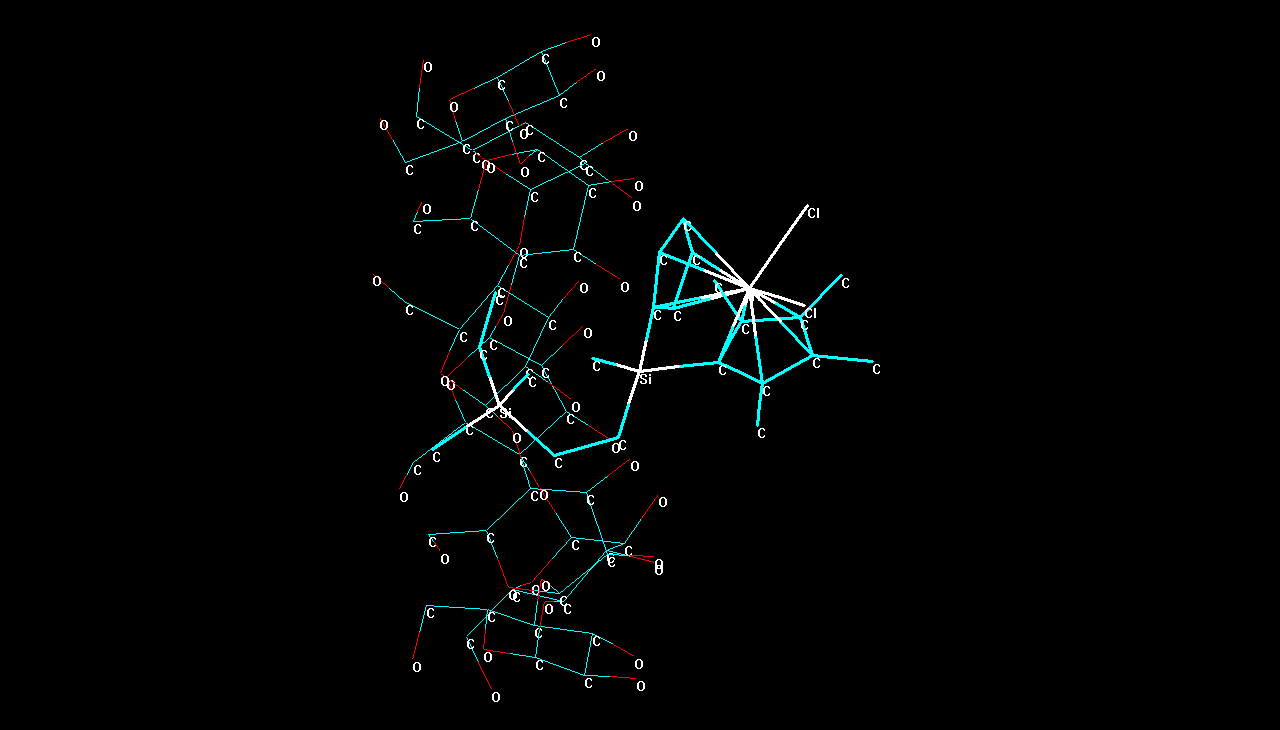


**Figure A3.9.** Starting position (up) and the minimum energy supramolecular system (down) for titanocene (code 03TC) / γ-cyclodextrin complex (theoretically modeled by MM+ docking experiments)


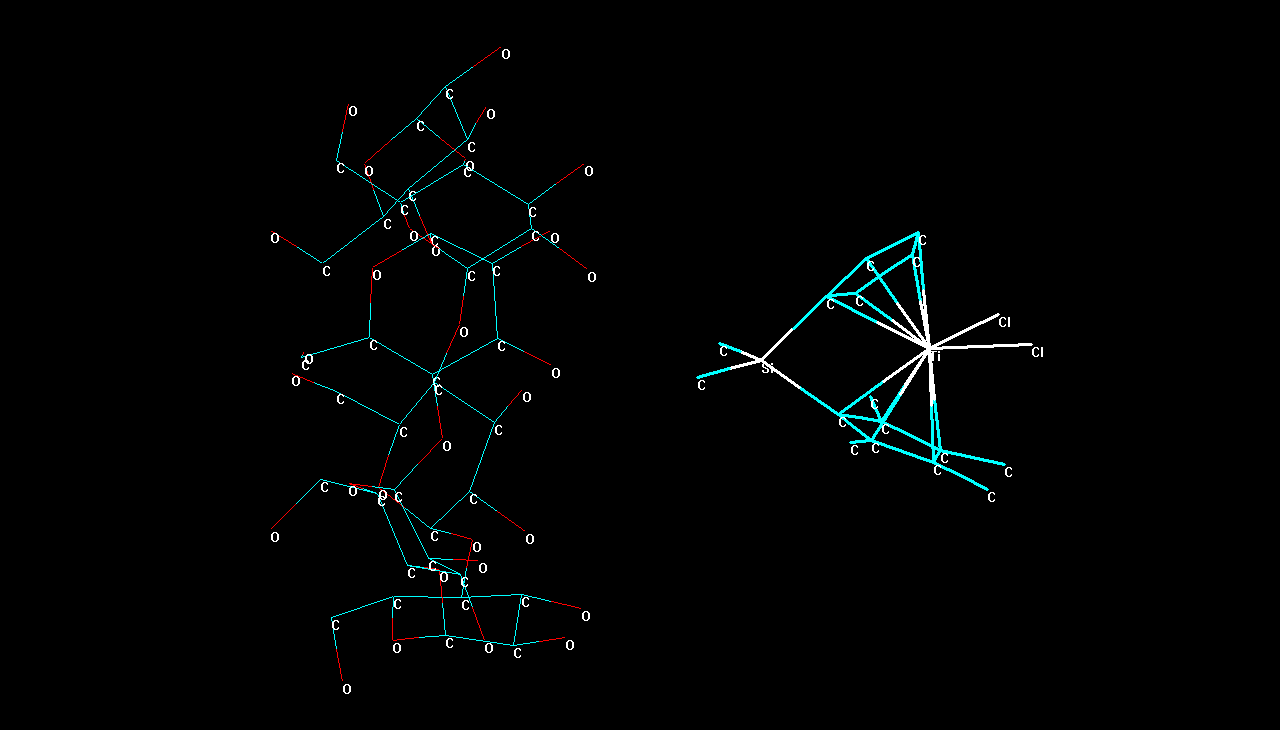


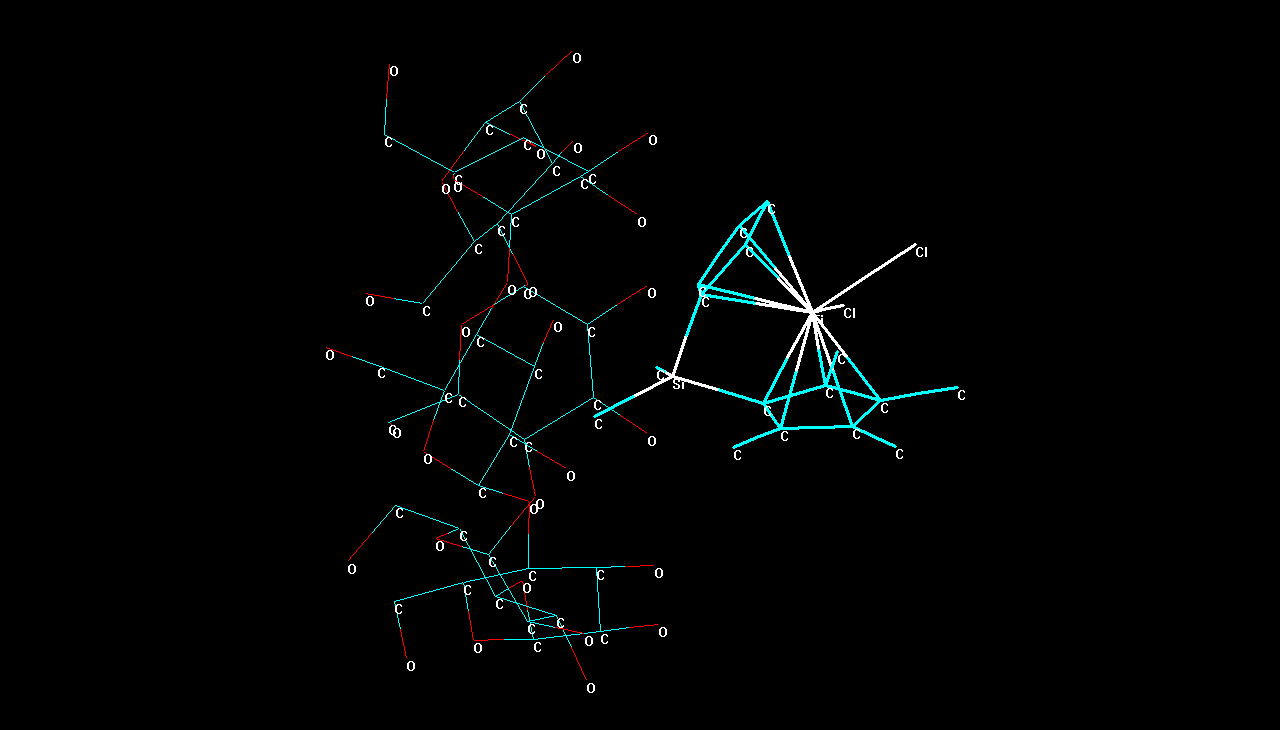


**Figure A3.10.** Starting position (up) and the minimum energy supramolecular system (down) for titanocene (code 08TC) / α-cyclodextrin complex (theoretically modeled by MM+ docking experiments)


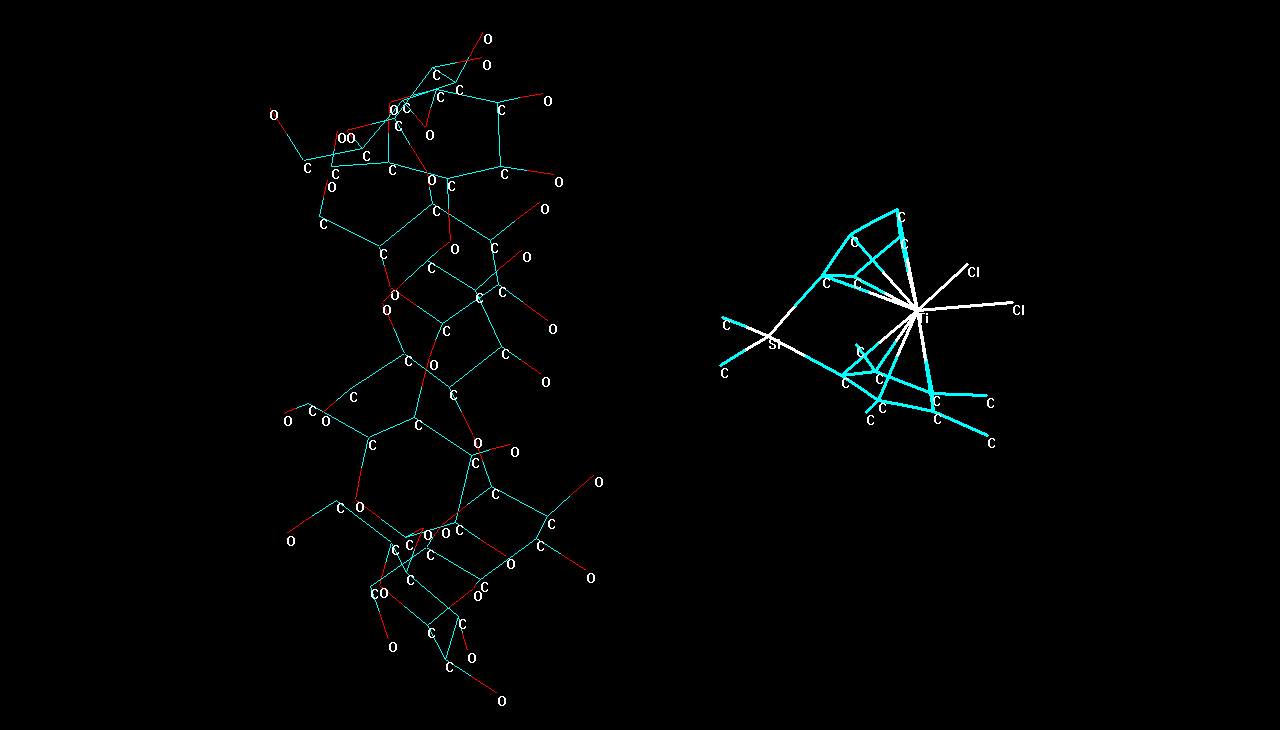


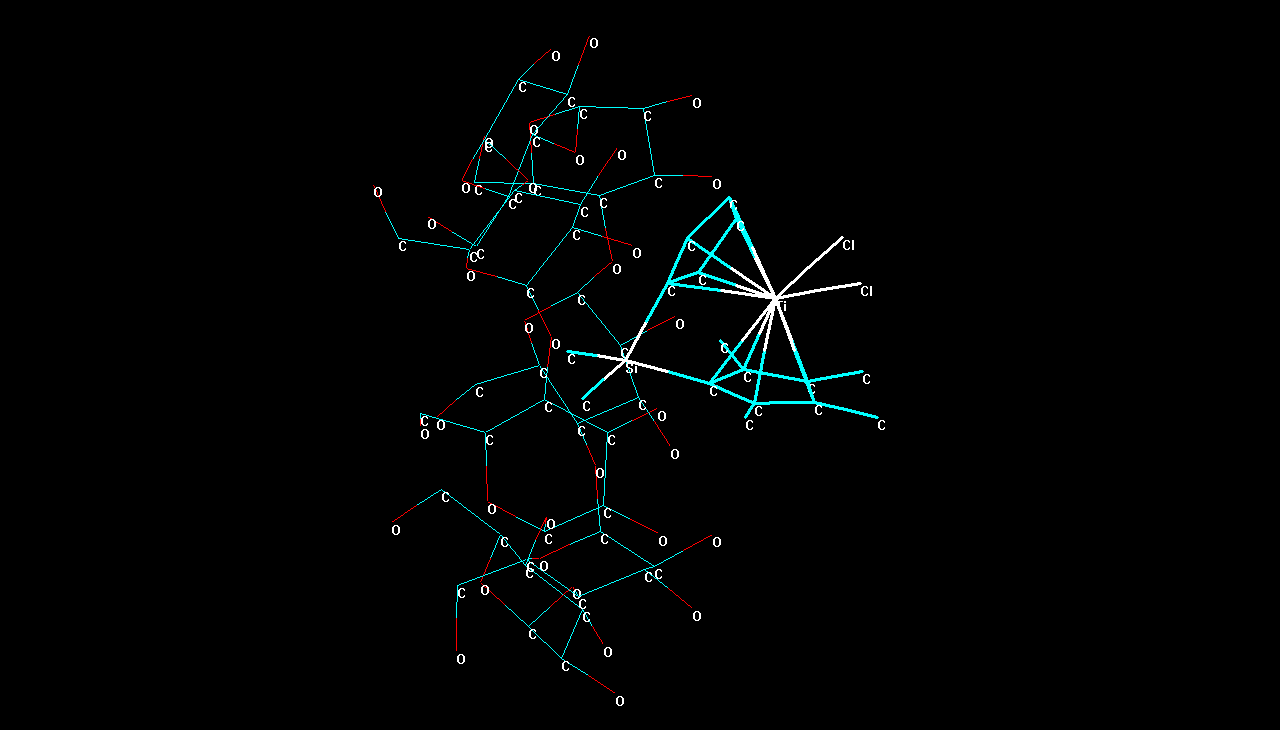


**Figure A3.11.** Starting position (up) and the minimum energy supramolecular system (down) for titanocene (code 08TC) / β-cyclodextrin complex (theoretically modeled by MM+ docking experiments)


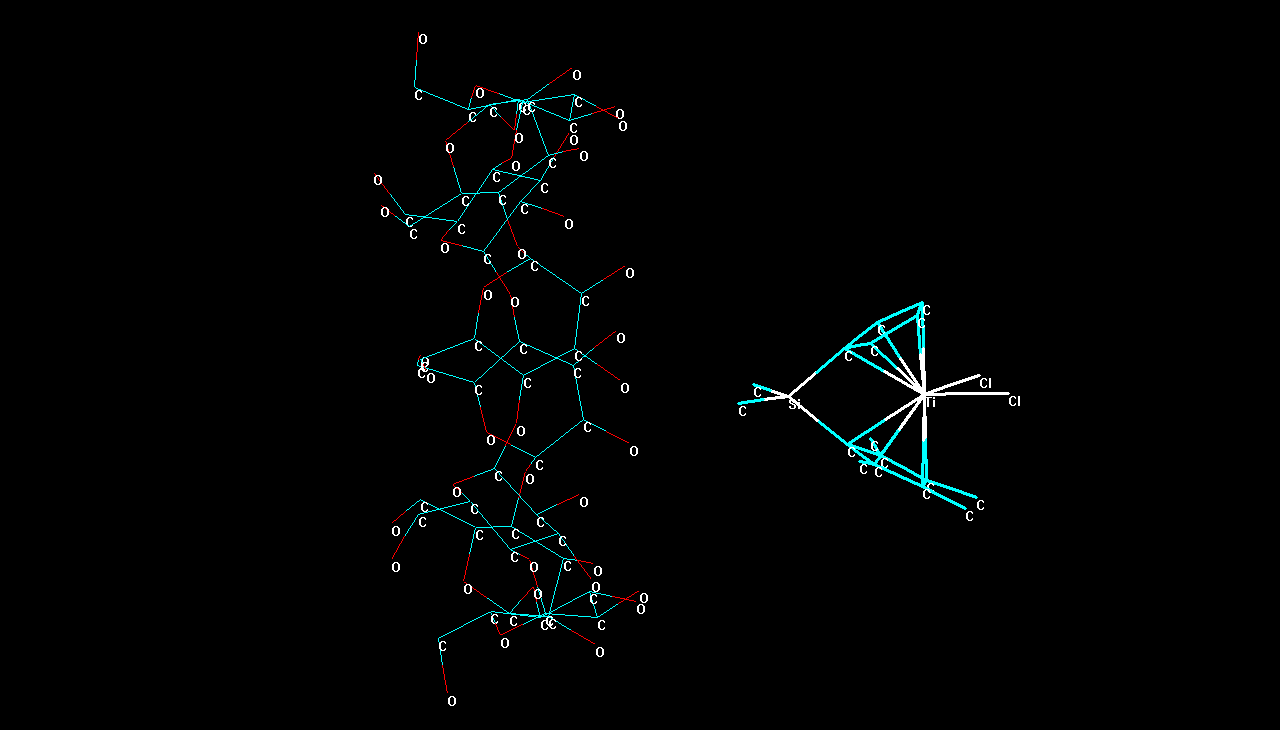


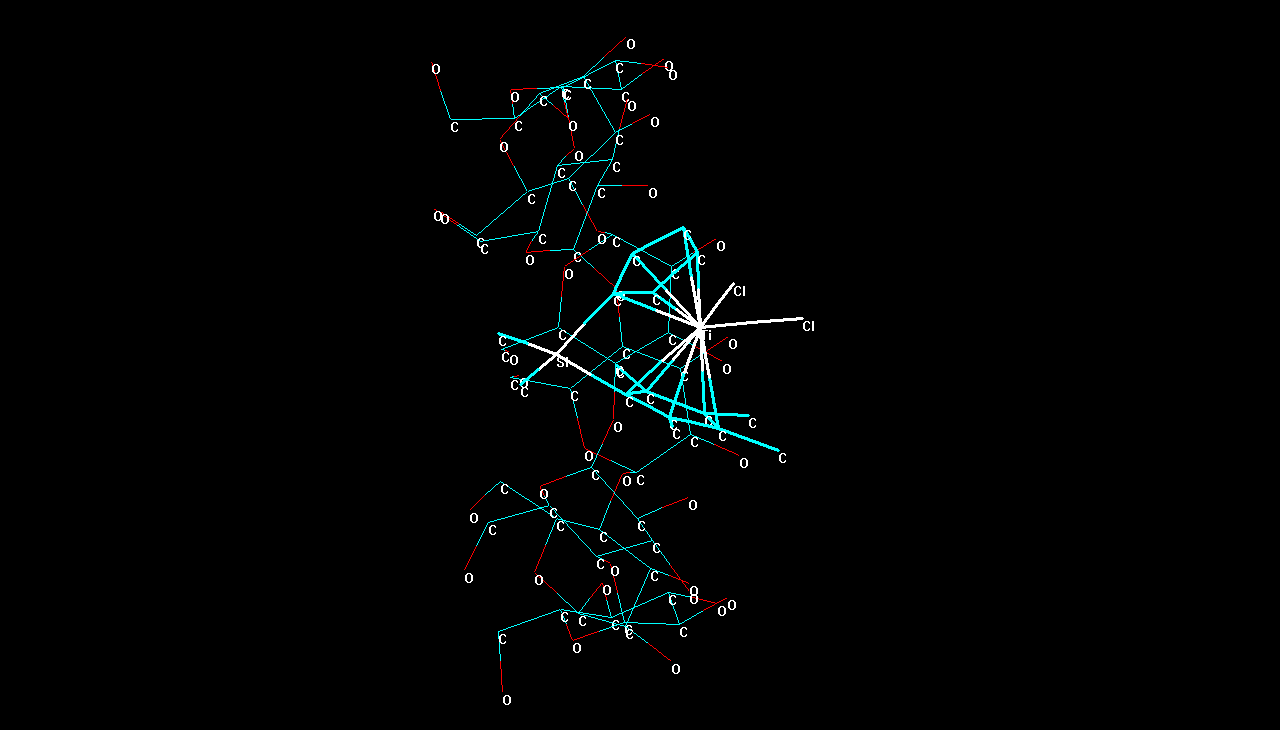


**Figure A3.12.** Starting position (up) and the minimum energy supramolecular system (down) for titanocene (code 08TC) / γ-cyclodextrin complex (theoretically modeled by MM+ docking experiments)


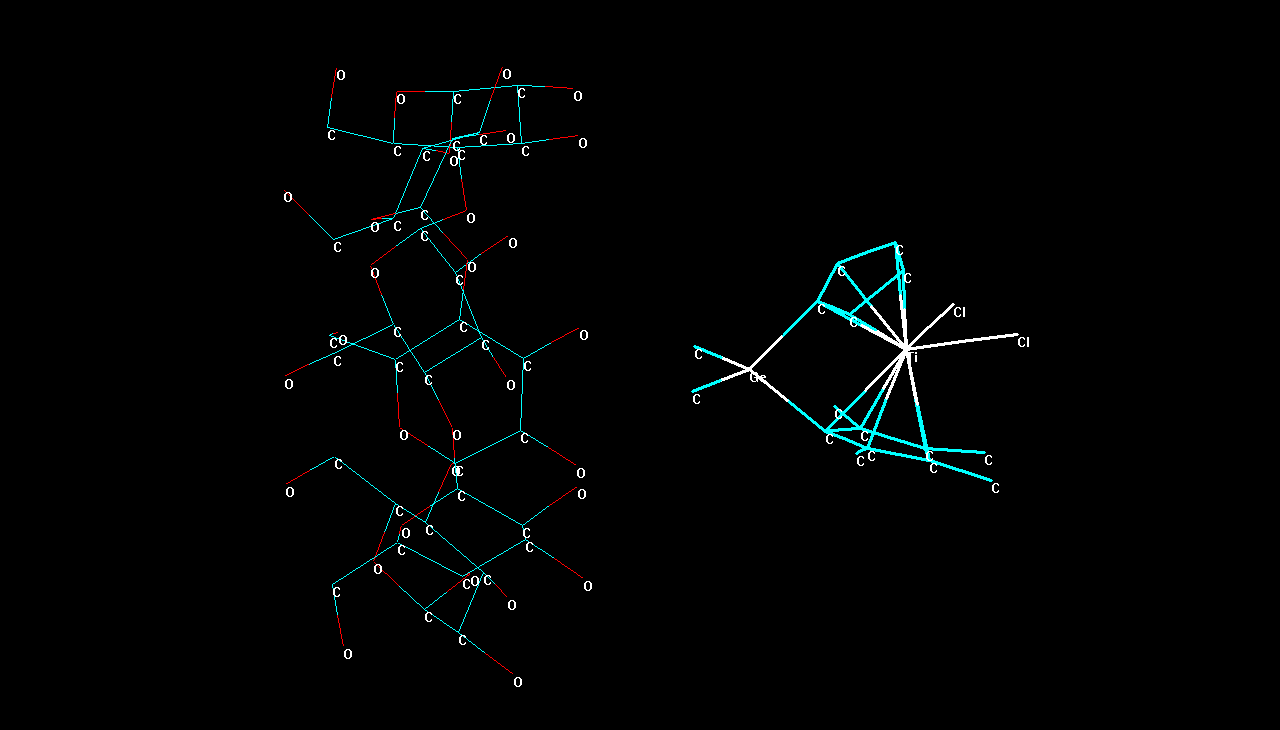


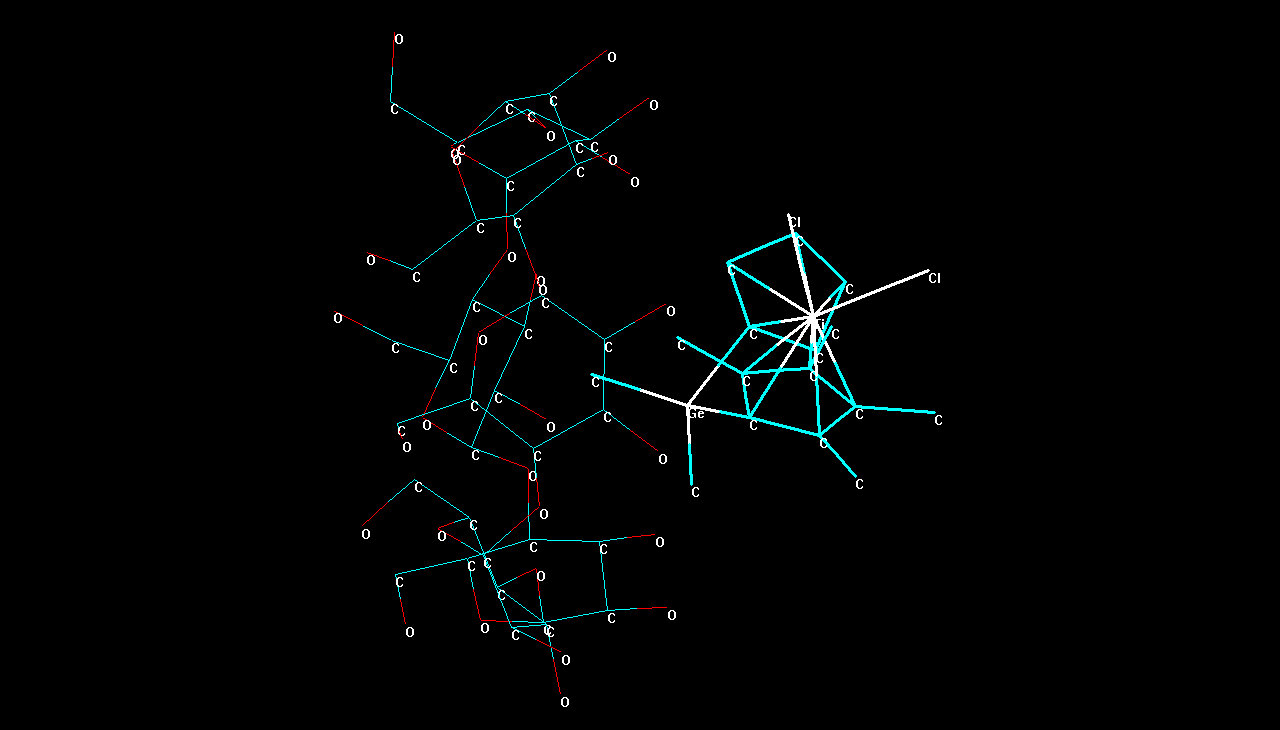


**Figure A3.13.** Starting position (up) and the minimum energy supramolecular system (down) for titanocene (code 09TC) / α-cyclodextrin complex (theoretically modeled by MM+ docking experiments)


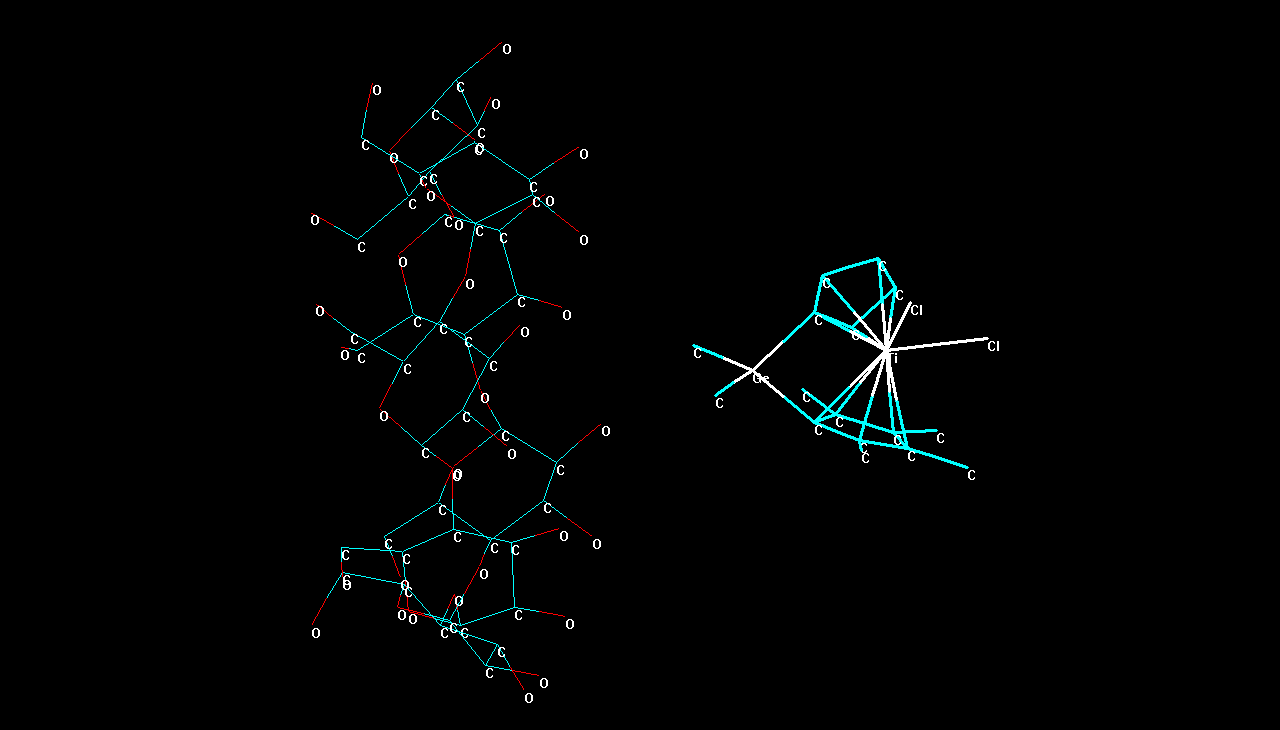


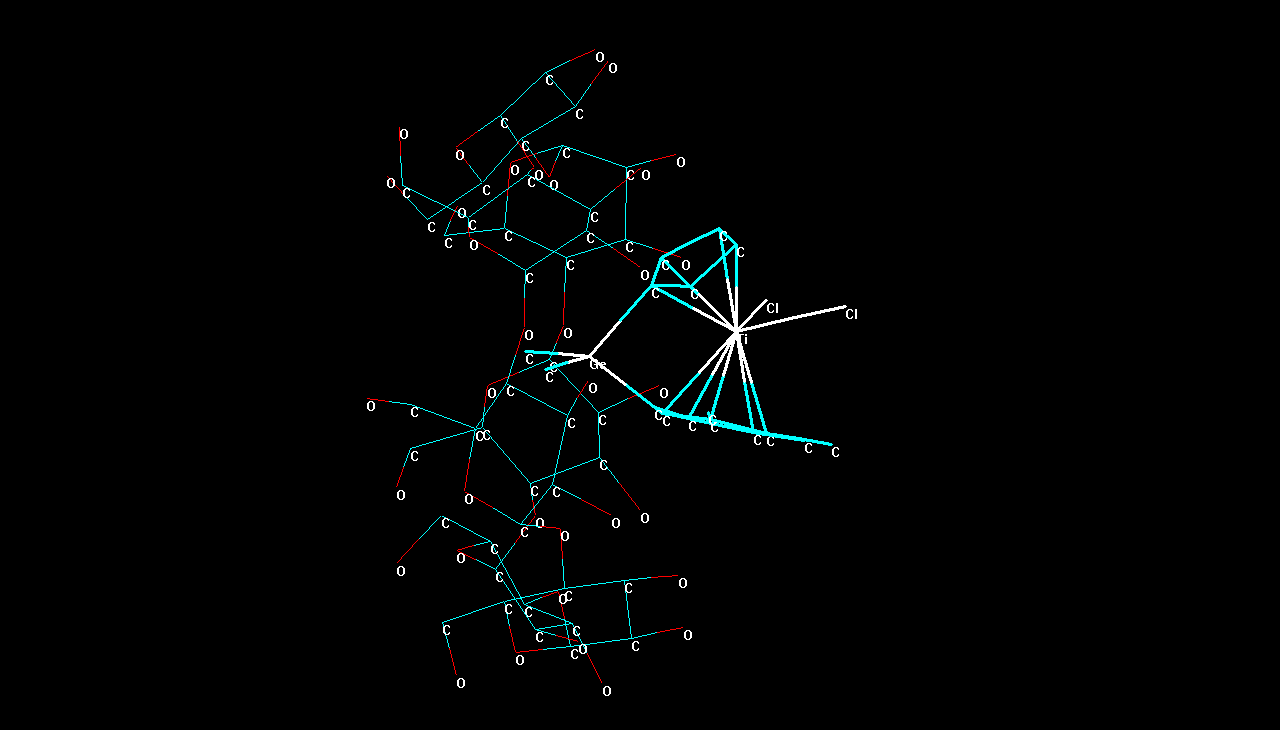


**Figure A3.14.** Starting position (up) and the minimum energy supramolecular system (down) for titanocene (code 09TC) / β-cyclodextrin complex (theoretically modeled by MM+ docking experiments)


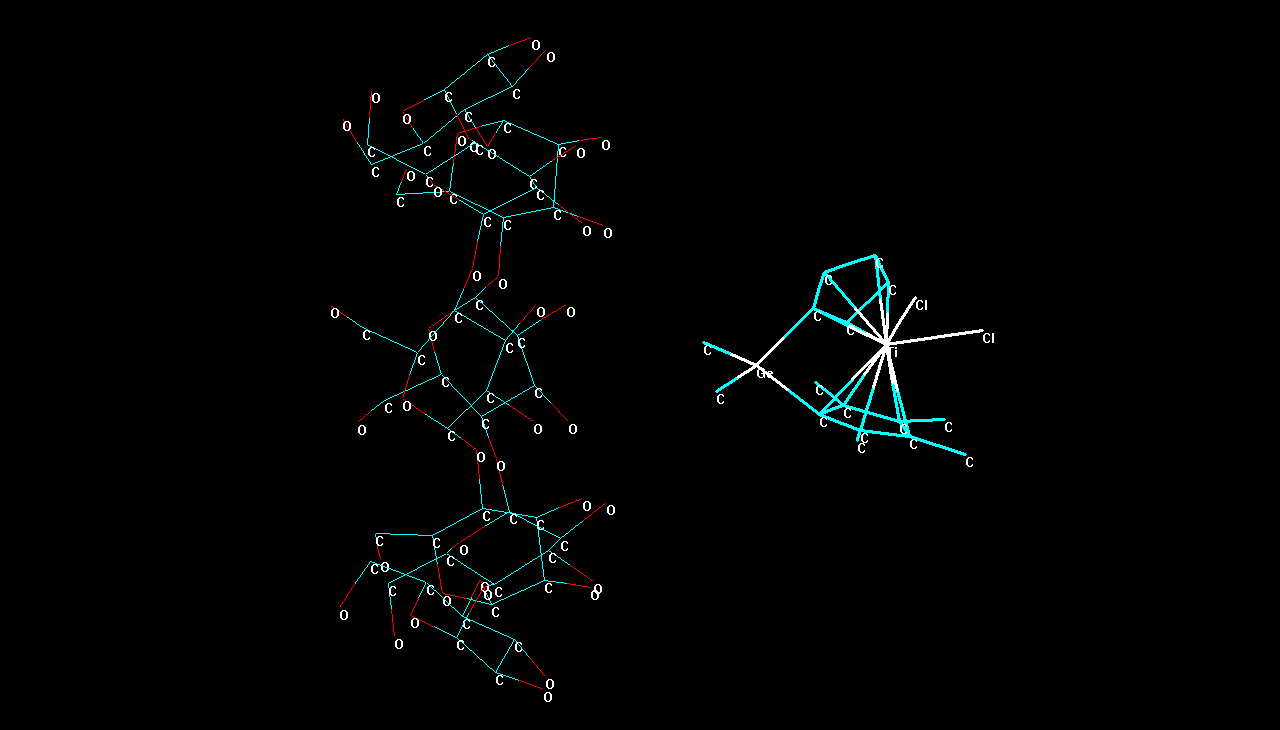


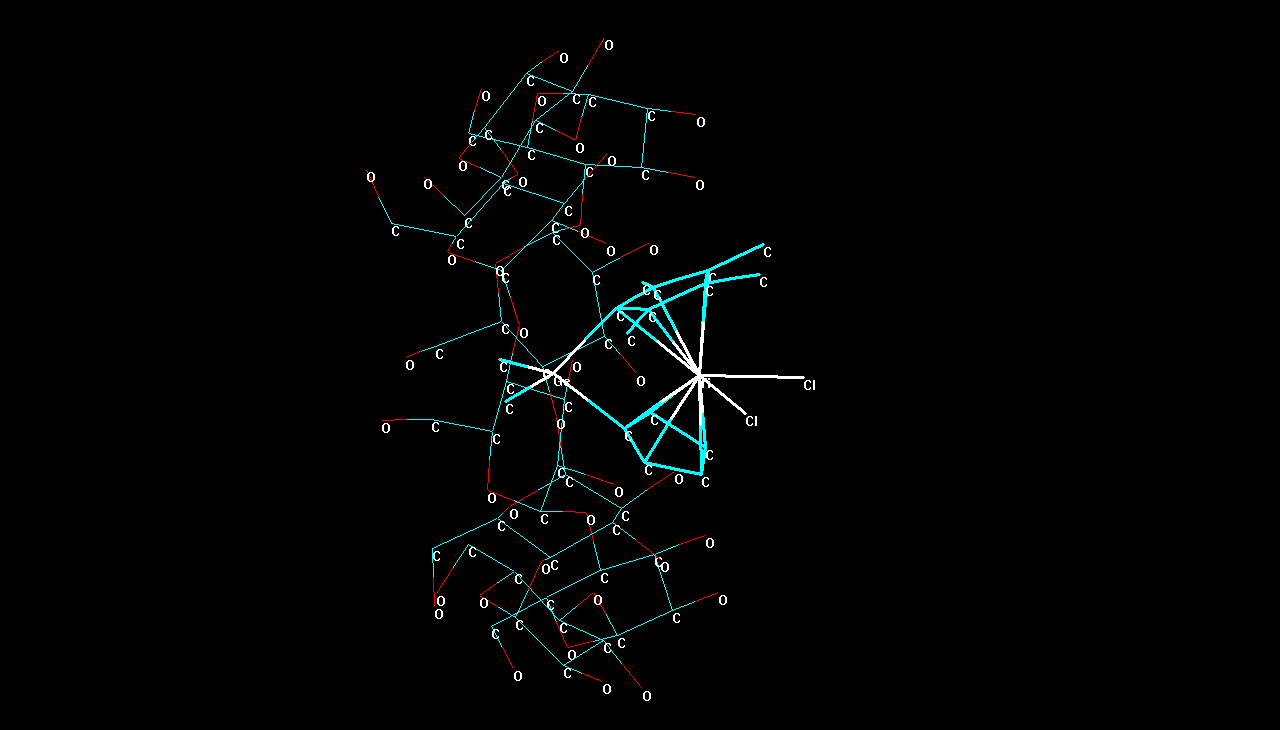


**Figure A3.15.** Starting position (up) and the minimum energy supramolecular system (down) for titanocene (code 09TC) / γ-cyclodextrin complex (theoretically modeled by MM+ docking experiments)


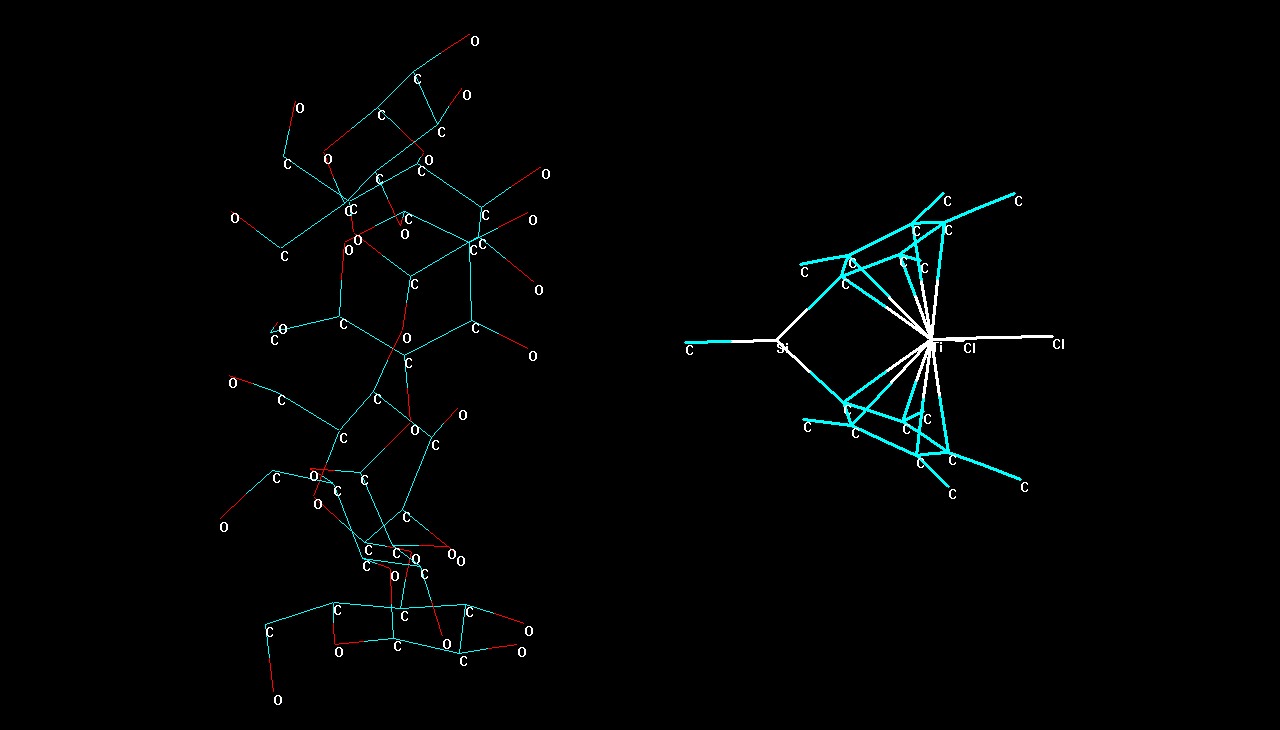


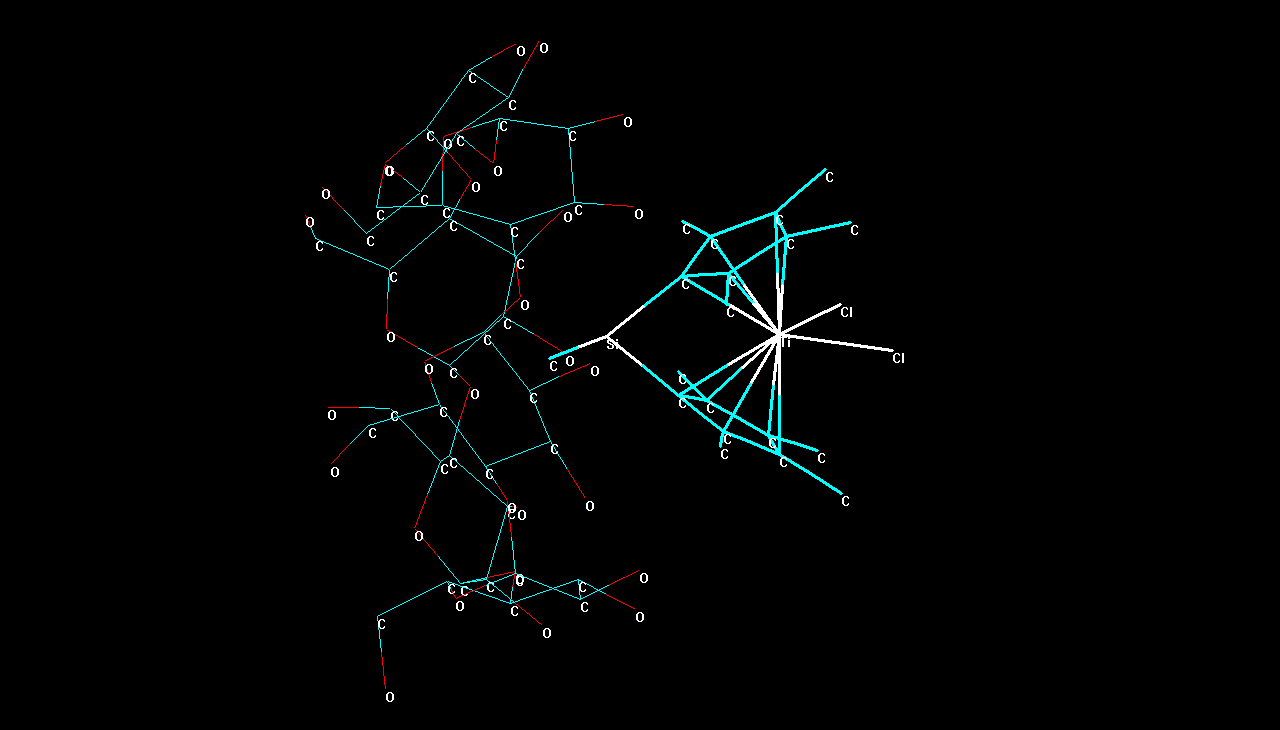


**Figure A3.16.** Starting position (up) and the minimum energy supramolecular system (down) for titanocene (code 10TC) / α-cyclodextrin complex (theoretically modeled by MM+ docking experiments)


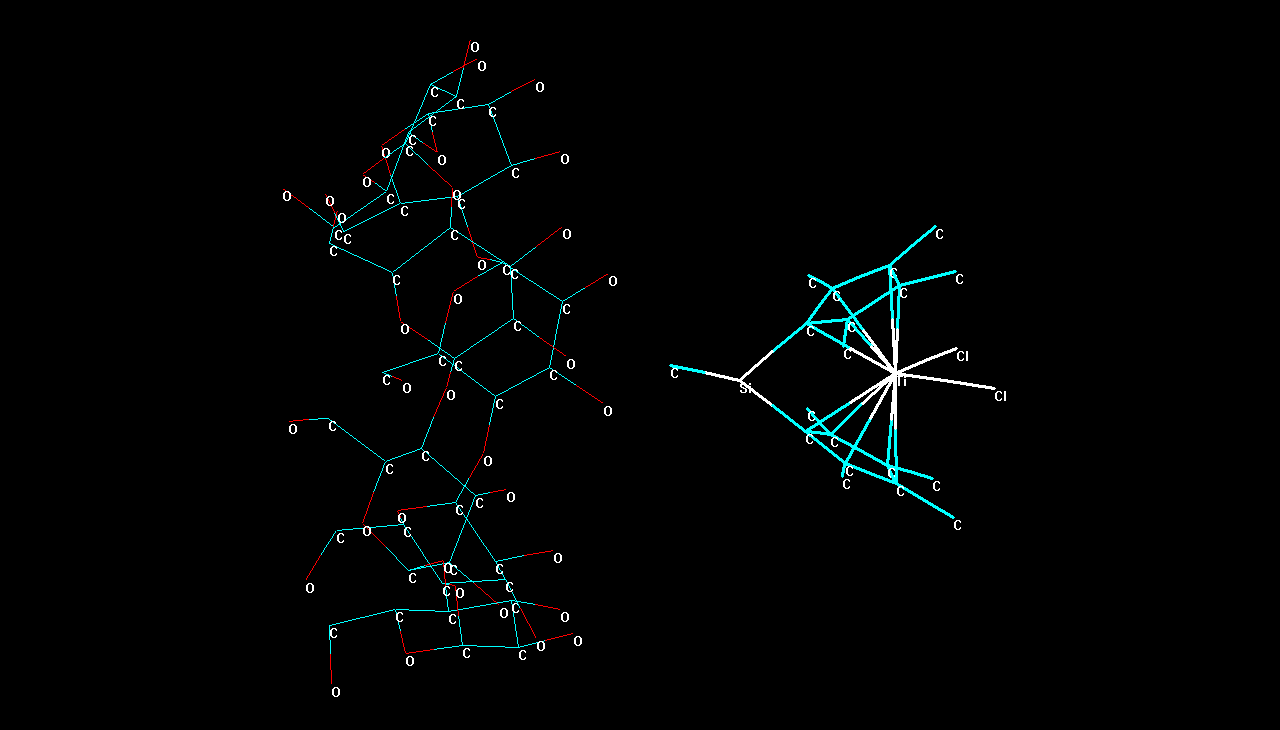


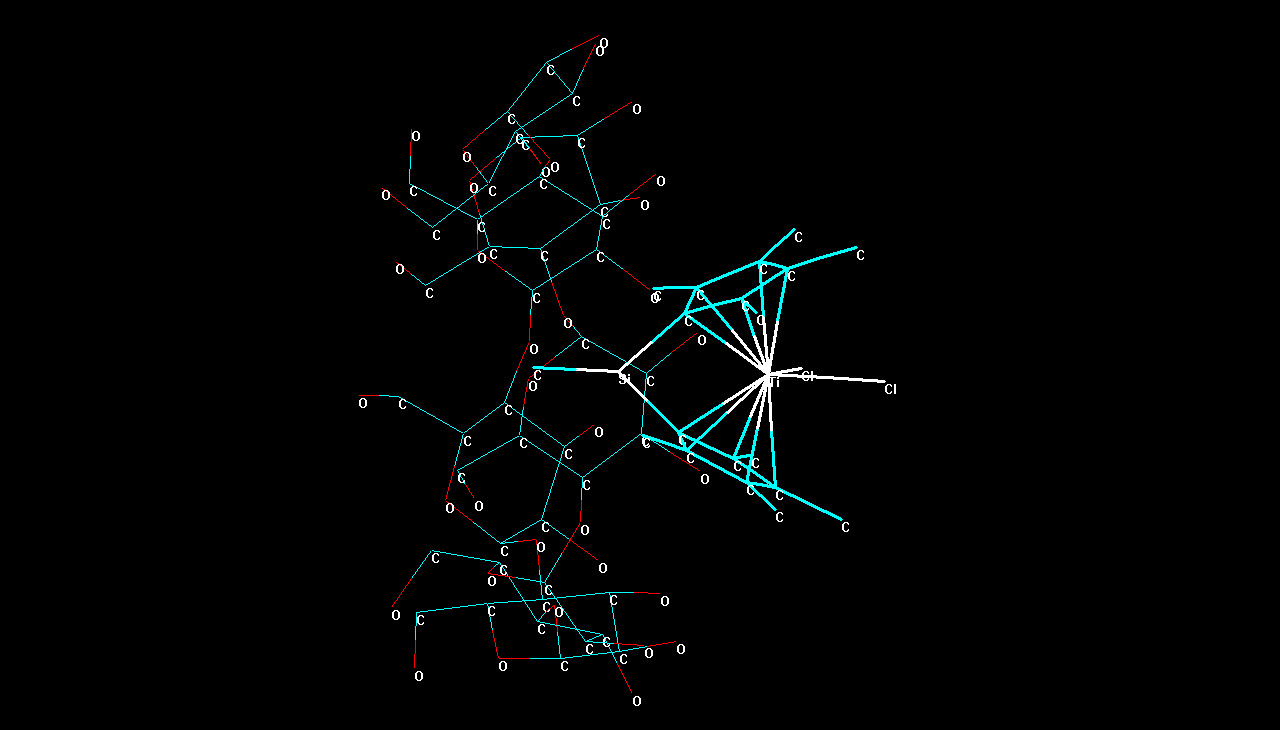


**Figure A3.17.** Starting position (up) and the minimum energy supramolecular system (down) for titanocene (code 10TC) / β-cyclodextrin complex (theoretically modeled by MM+ docking experiments)


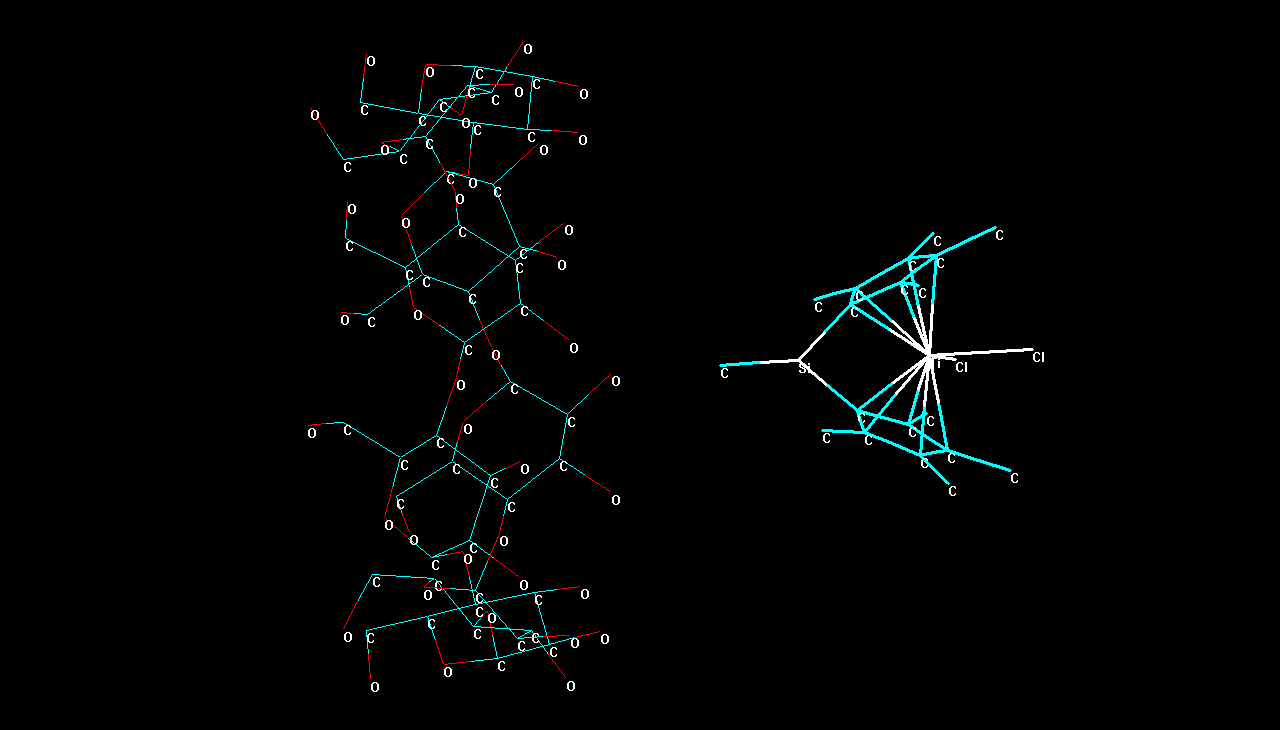


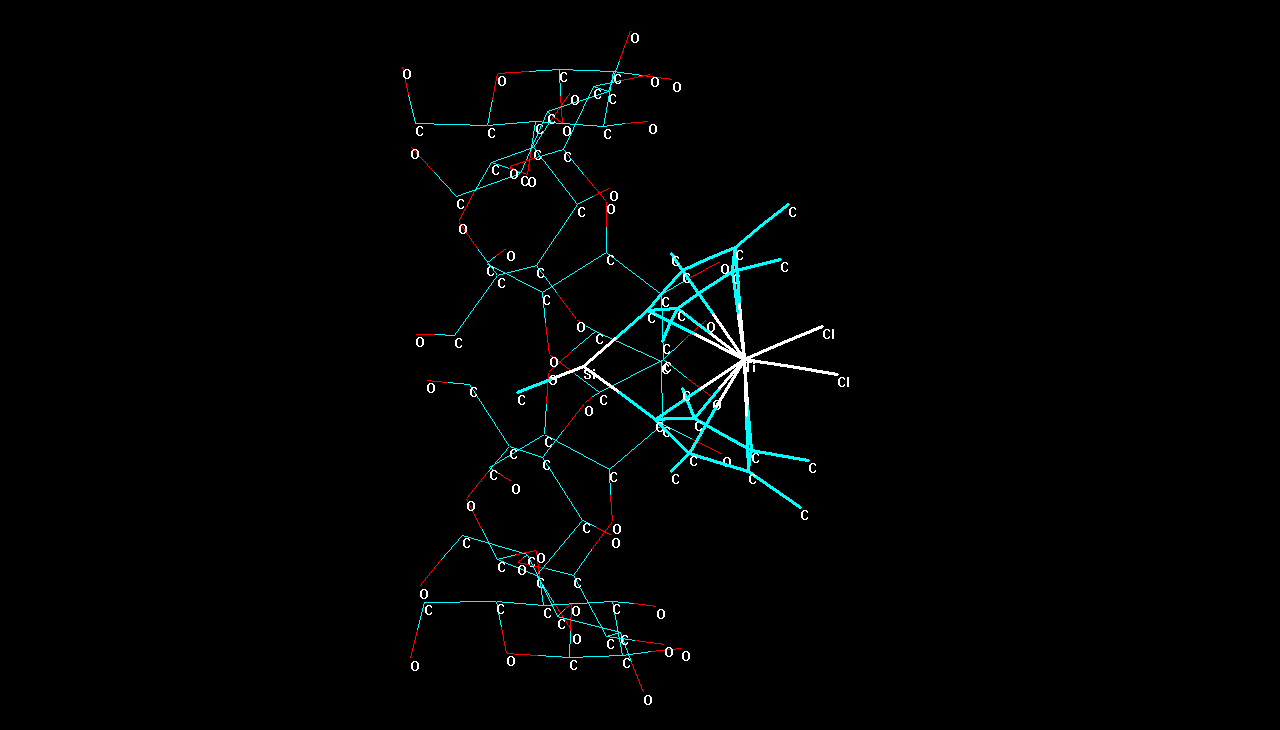


**Figure A3.18.** Starting position (up) and the minimum energy supramolecular system (down) for titanocene (code 10TC) / γ-cyclodextrin complex (theoretically modeled by MM+ docking experiments)


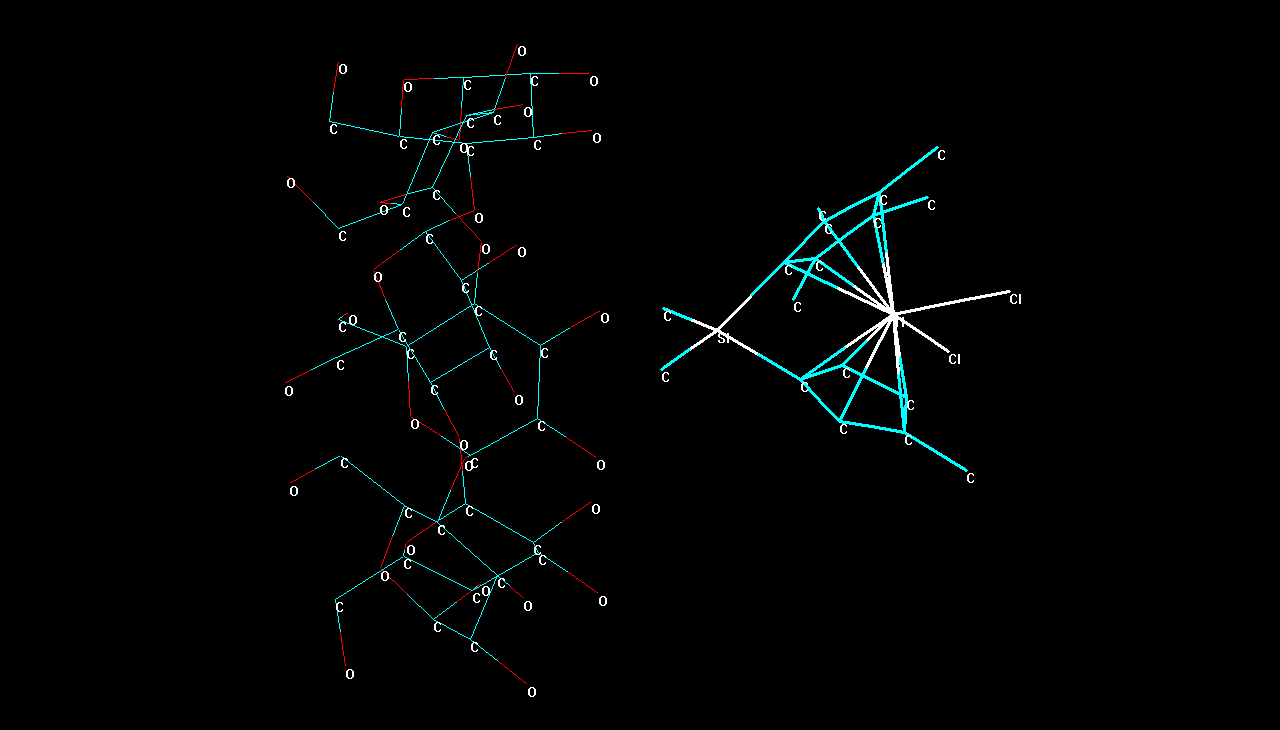


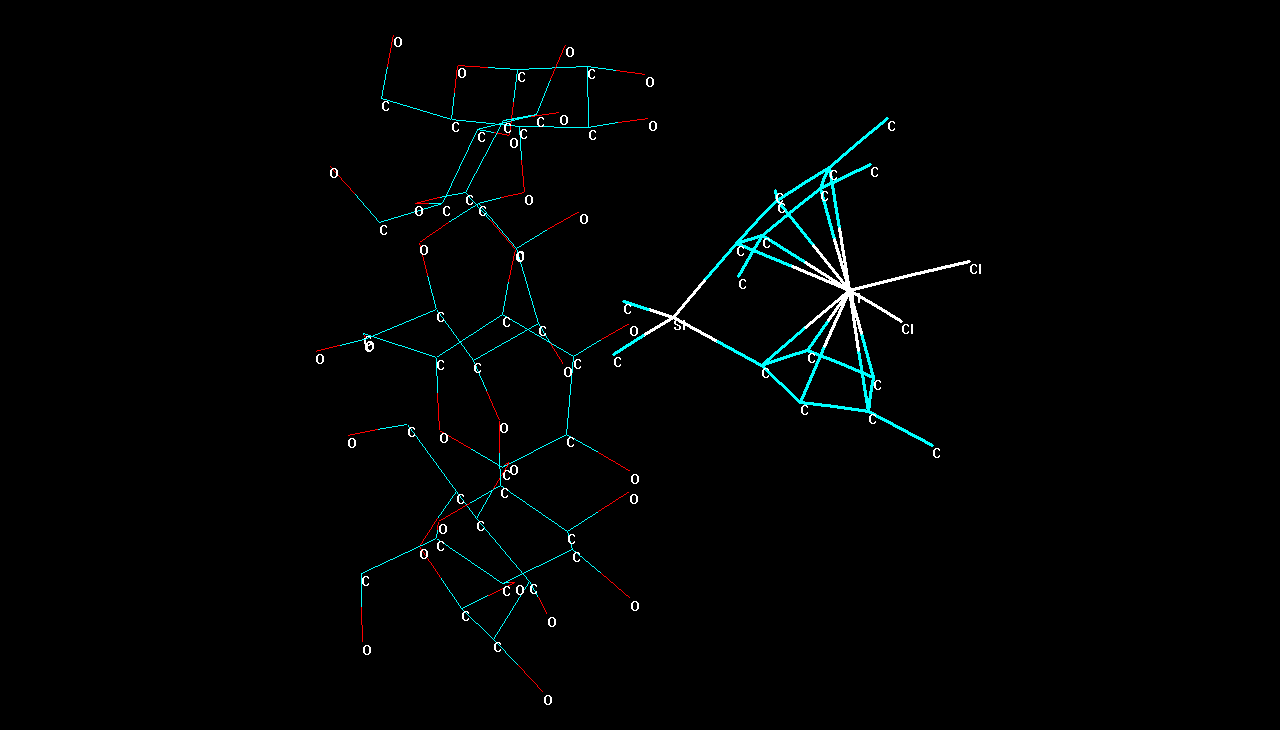


**Figure A3.19.** Starting position (up) and the minimum energy supramolecular system (down) for titanocene (code 11TC) / α-cyclodextrin complex (theoretically modeled by MM+ docking experiments)


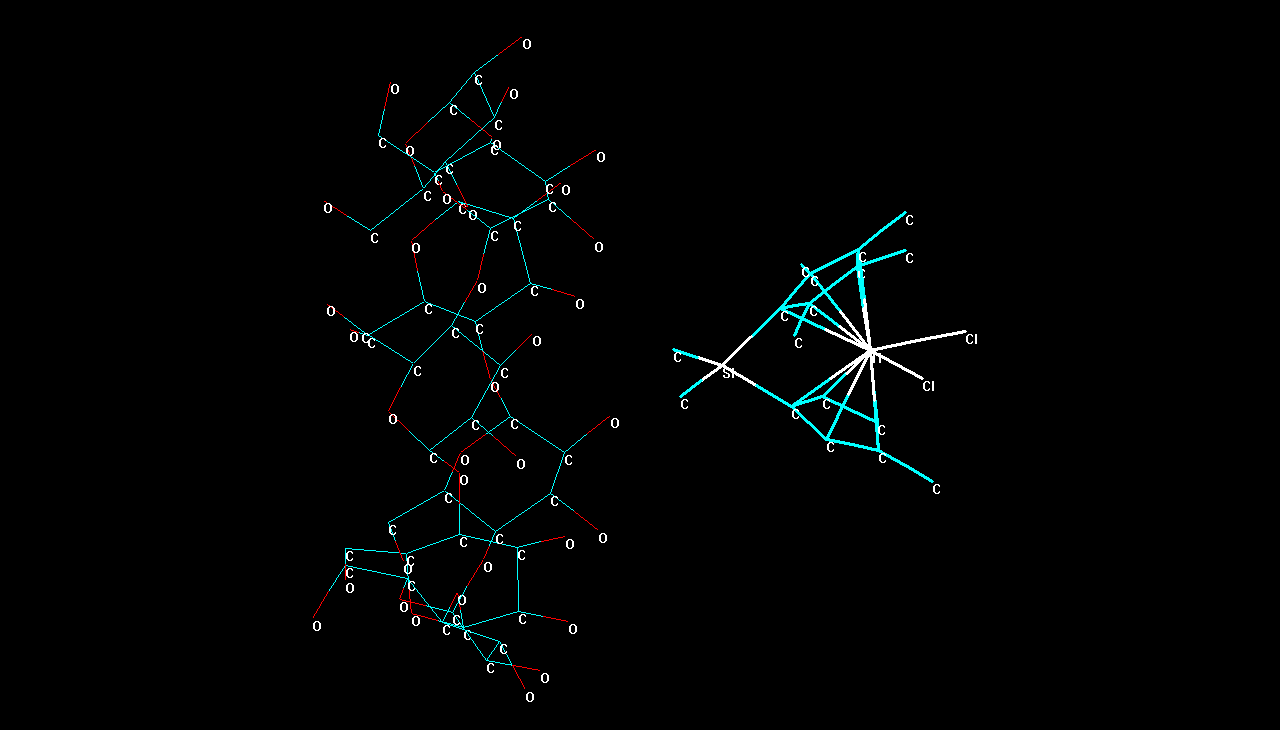


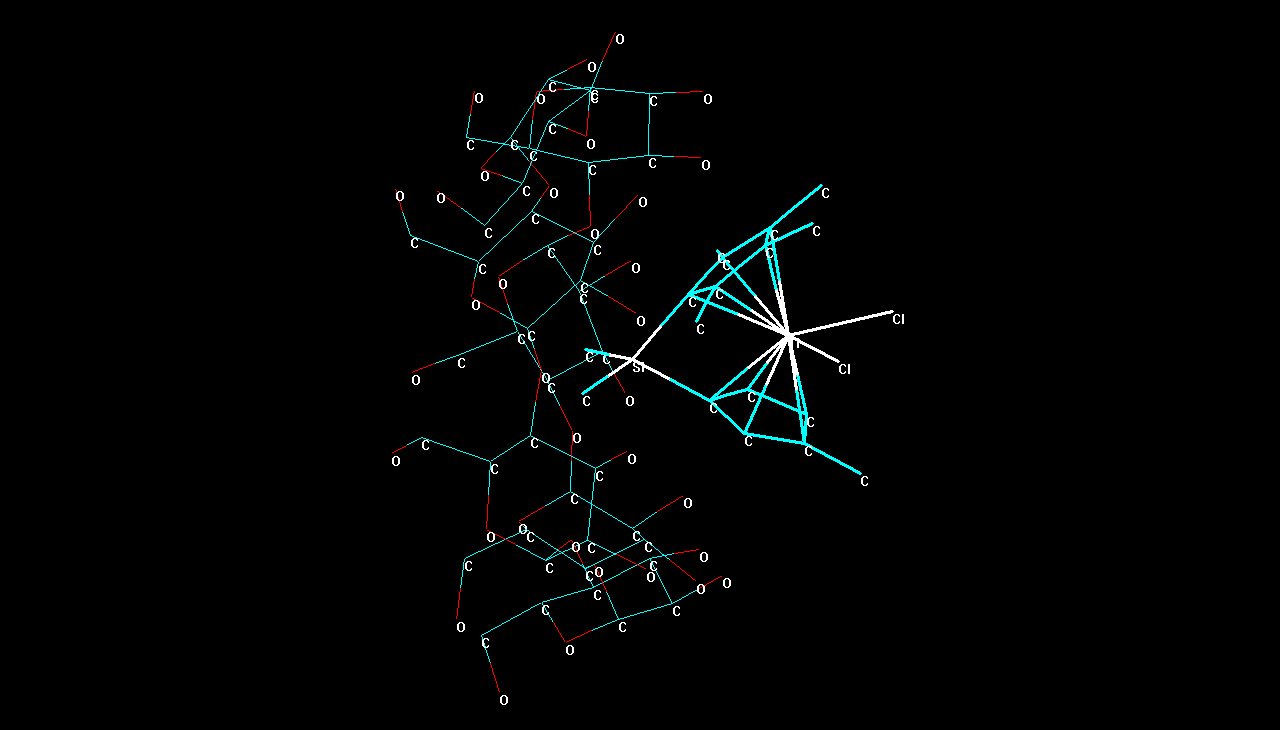


**Figure A3.20.** Starting position (up) and the minimum energy supramolecular system (down) for titanocene (code 11TC) / β-cyclodextrin complex (theoretically modeled by MM+ docking experiments)


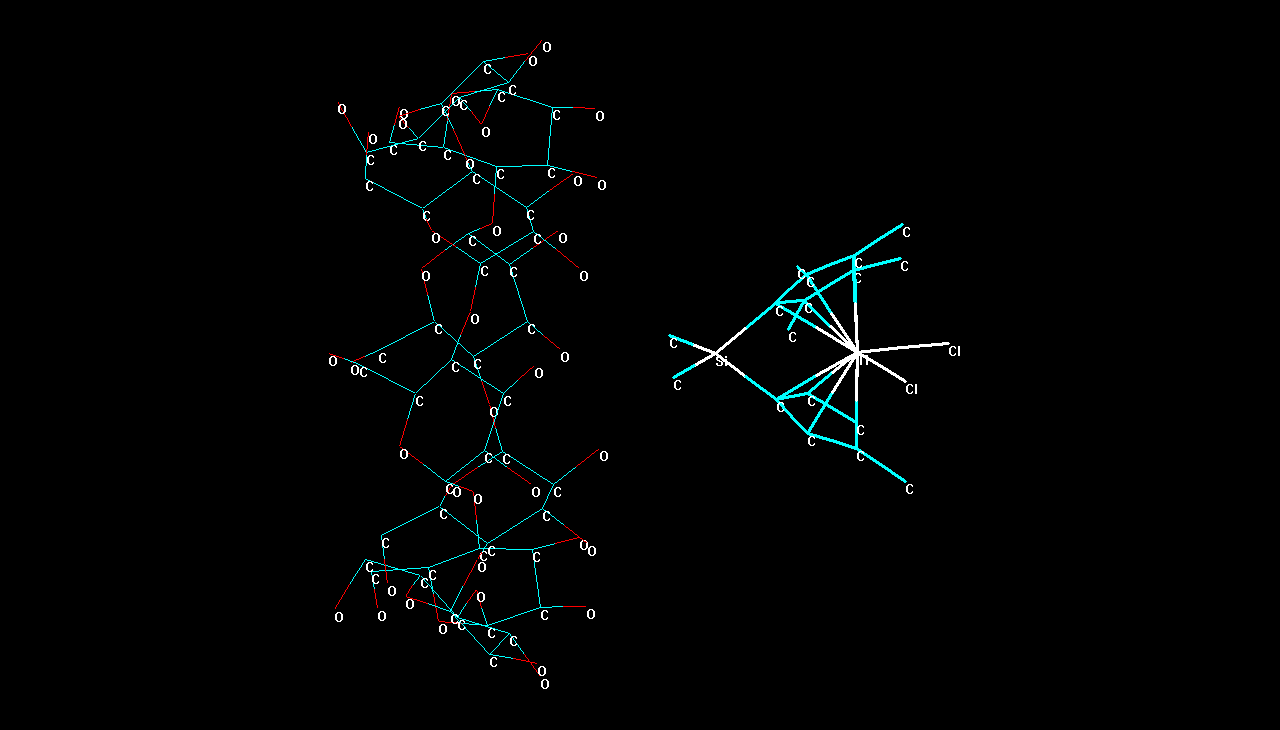


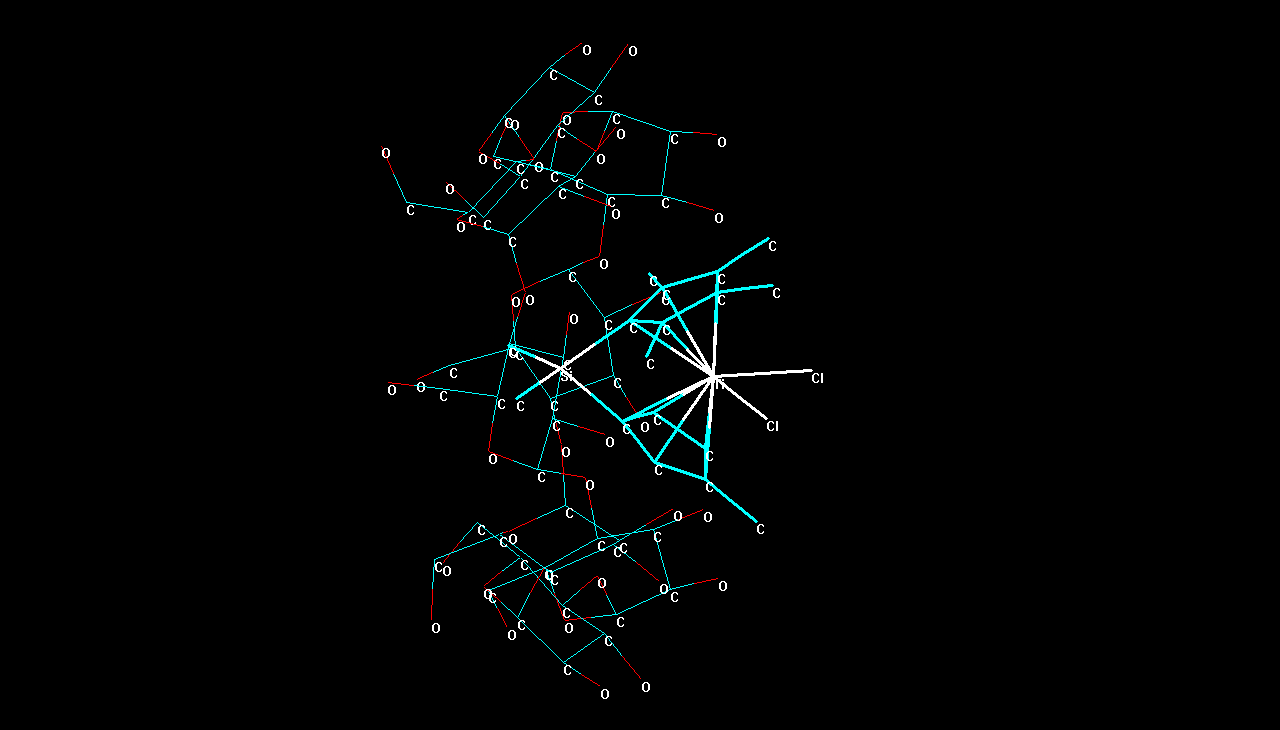


**Figure A3.21.** Starting position (up) and the minimum energy supramolecular system (down) for titanocene (code 11TC) / γ-cyclodextrin complex (theoretically modeled by MM+ docking experiments)


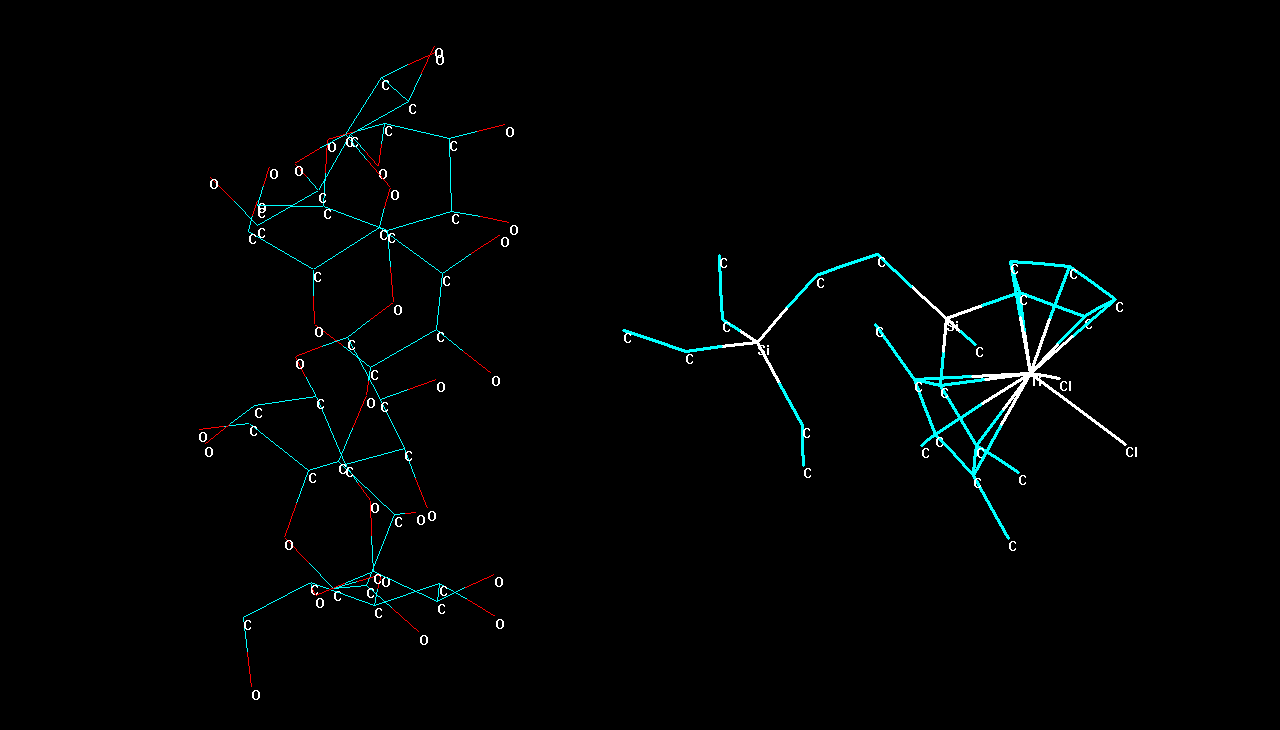


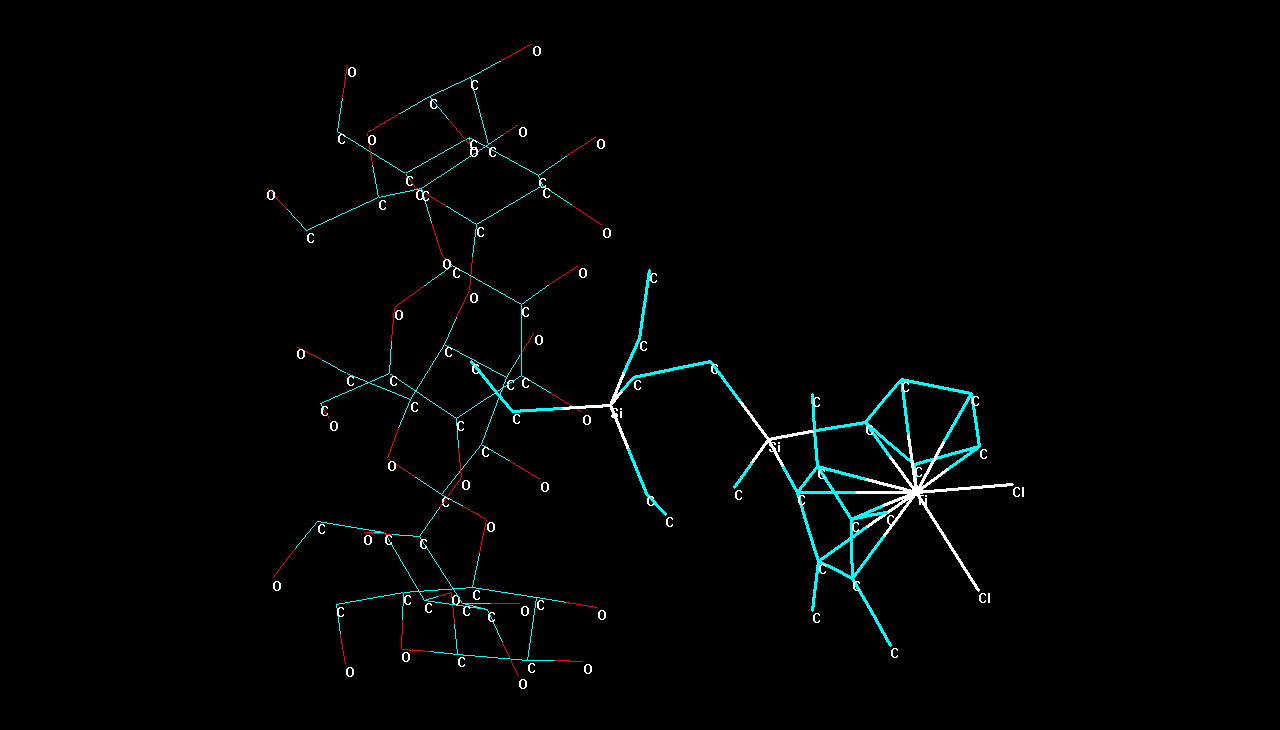


**Figure A3.22.** Starting position (up) and the minimum energy supramolecular system (down) for titanocene (code 18TC) / α-cyclodextrin complex (theoretically modeled by MM+ docking experiments)


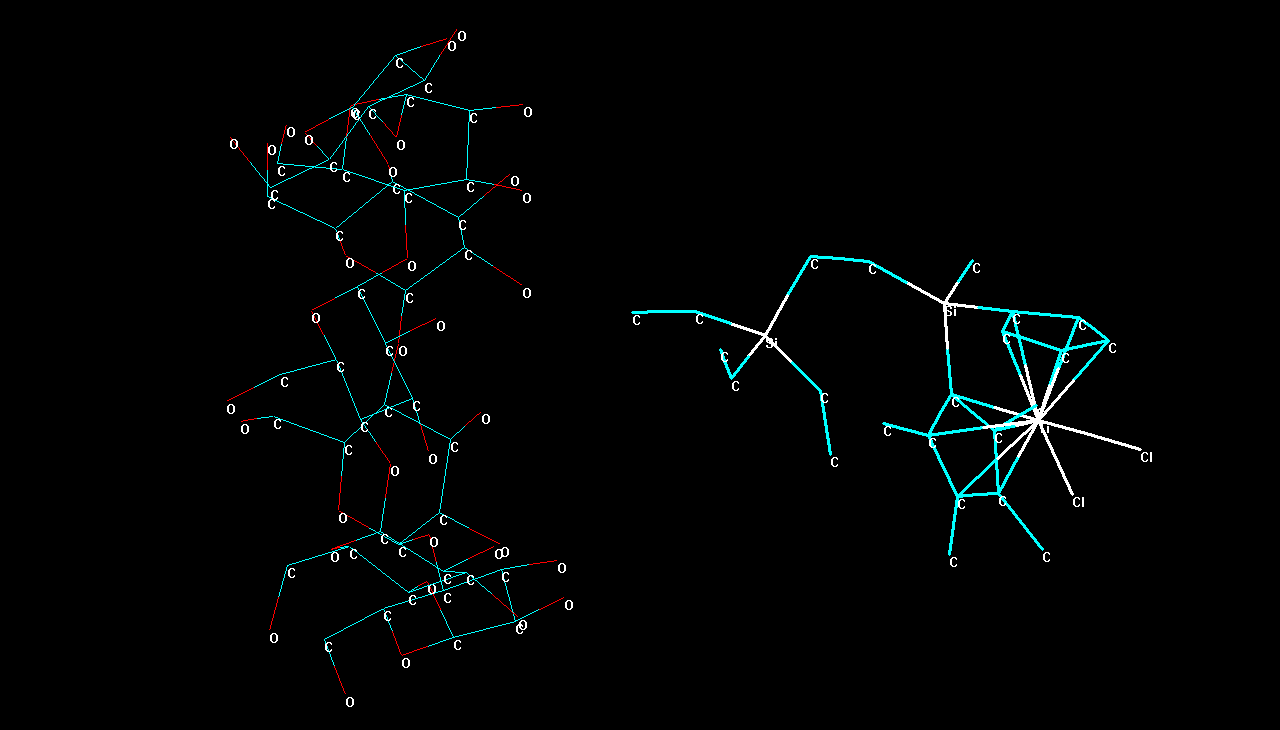


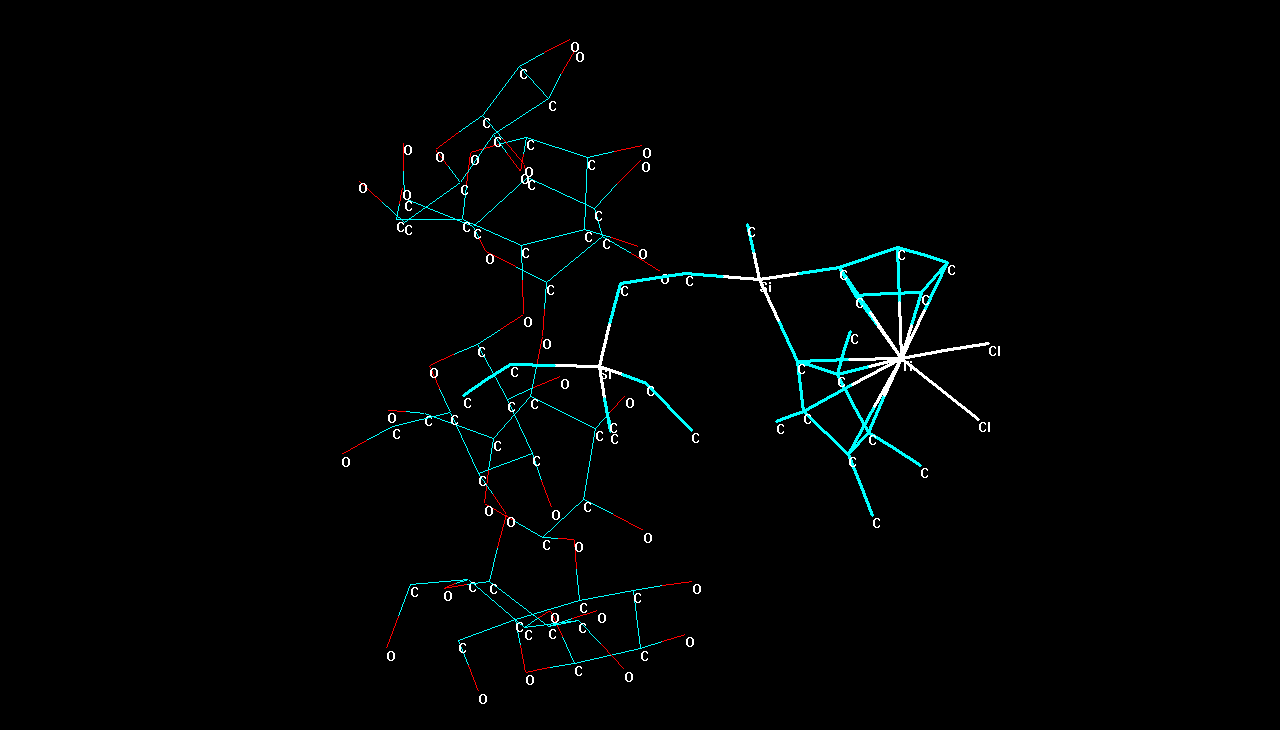


**Figure A3.23.** Starting position (up) and the minimum energy supramolecular system (down) for titanocene (code 18TC) / β-cyclodextrin complex (theoretically modeled by MM+ docking experiments)


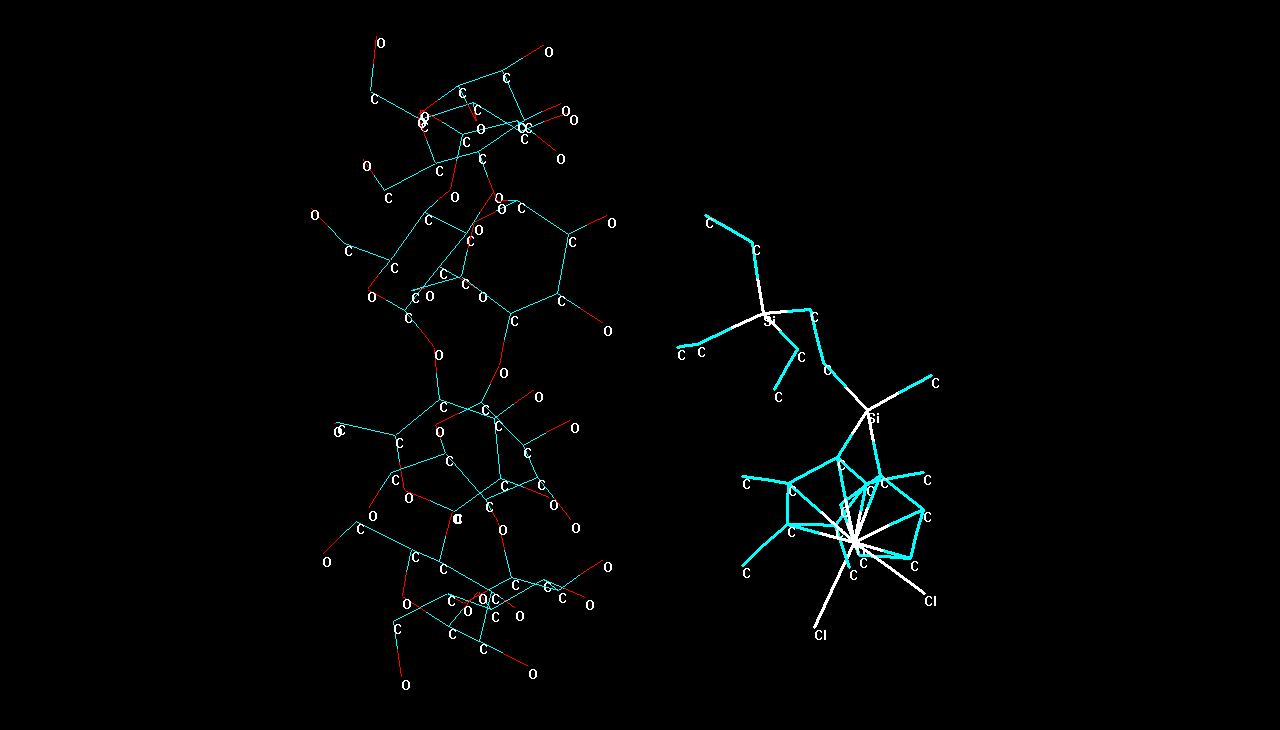


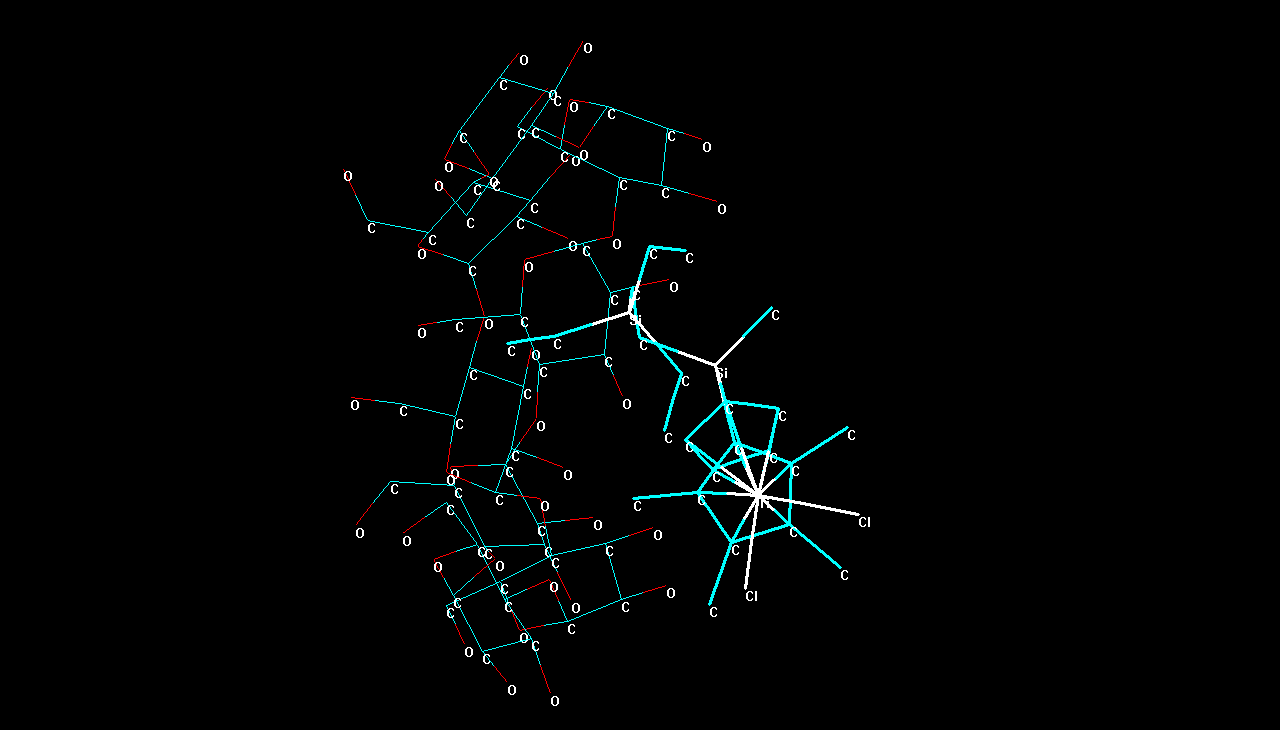


**Figure A3.24.** Starting position (up) and the minimum energy supramolecular system (down) for titanocene (code 18TC) / γ-cyclodextrin complex (theoretically modeled by MM+ docking experiments)


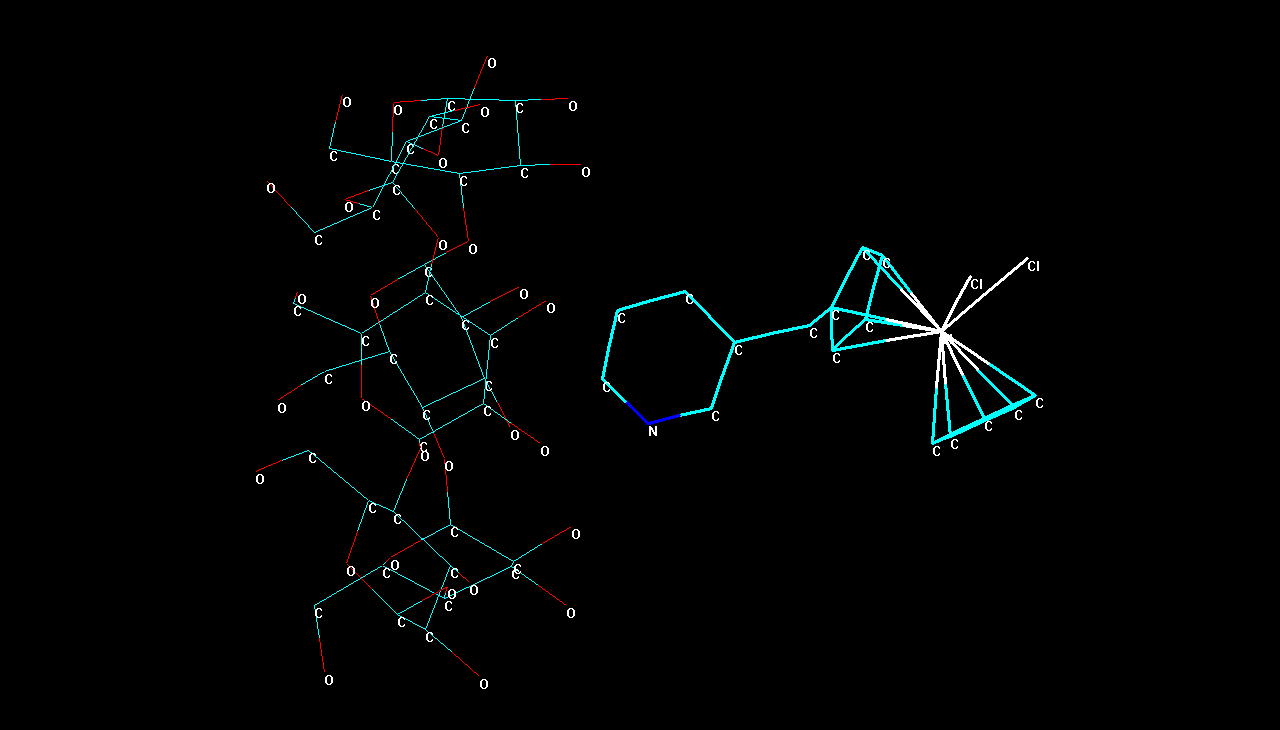


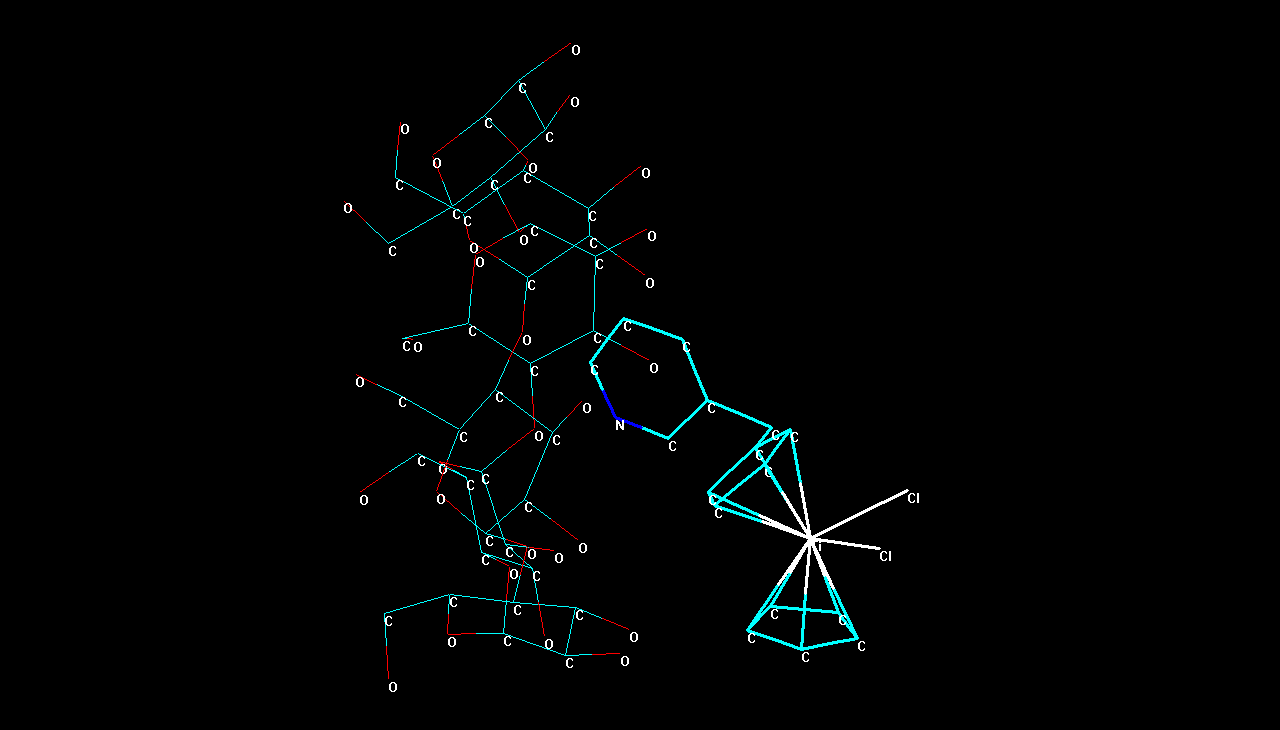


**Figure A3.25.** Starting position (up) and the minimum energy supramolecular system (down) for titanocene (code 23TC) / α-cyclodextrin complex (theoretically modeled by MM+ docking experiments)


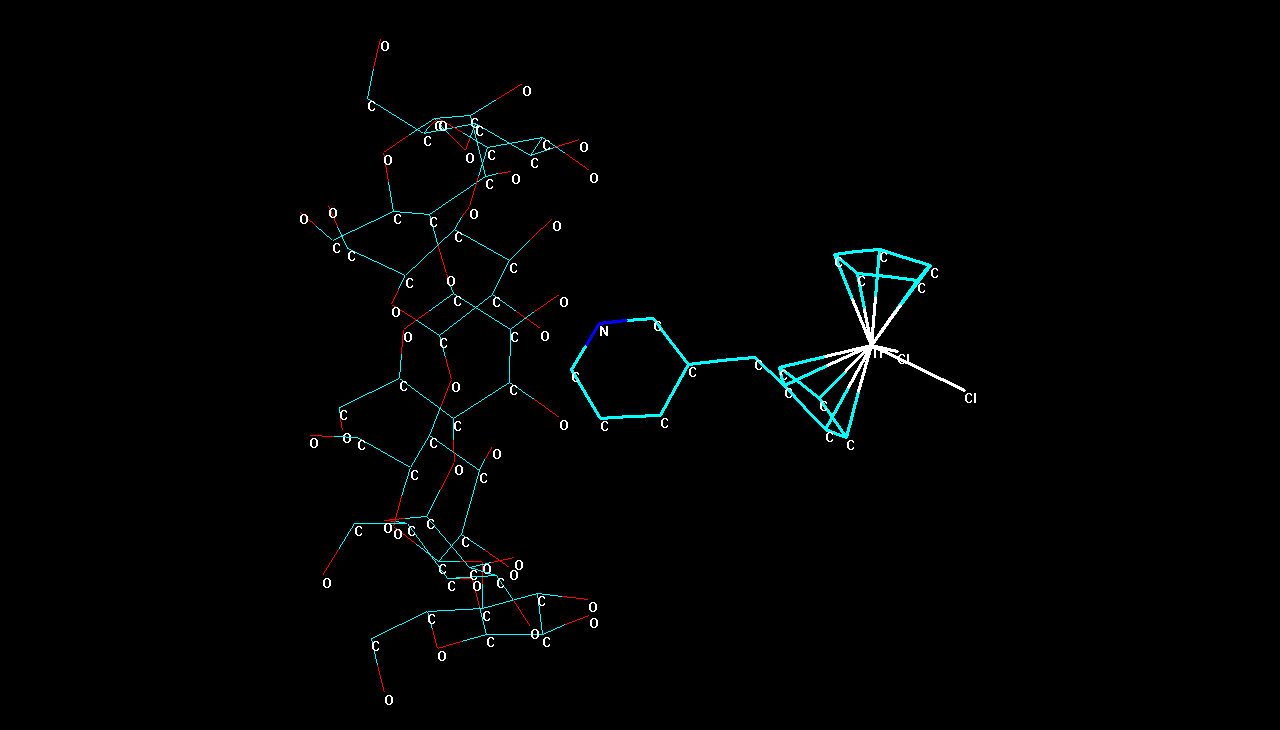


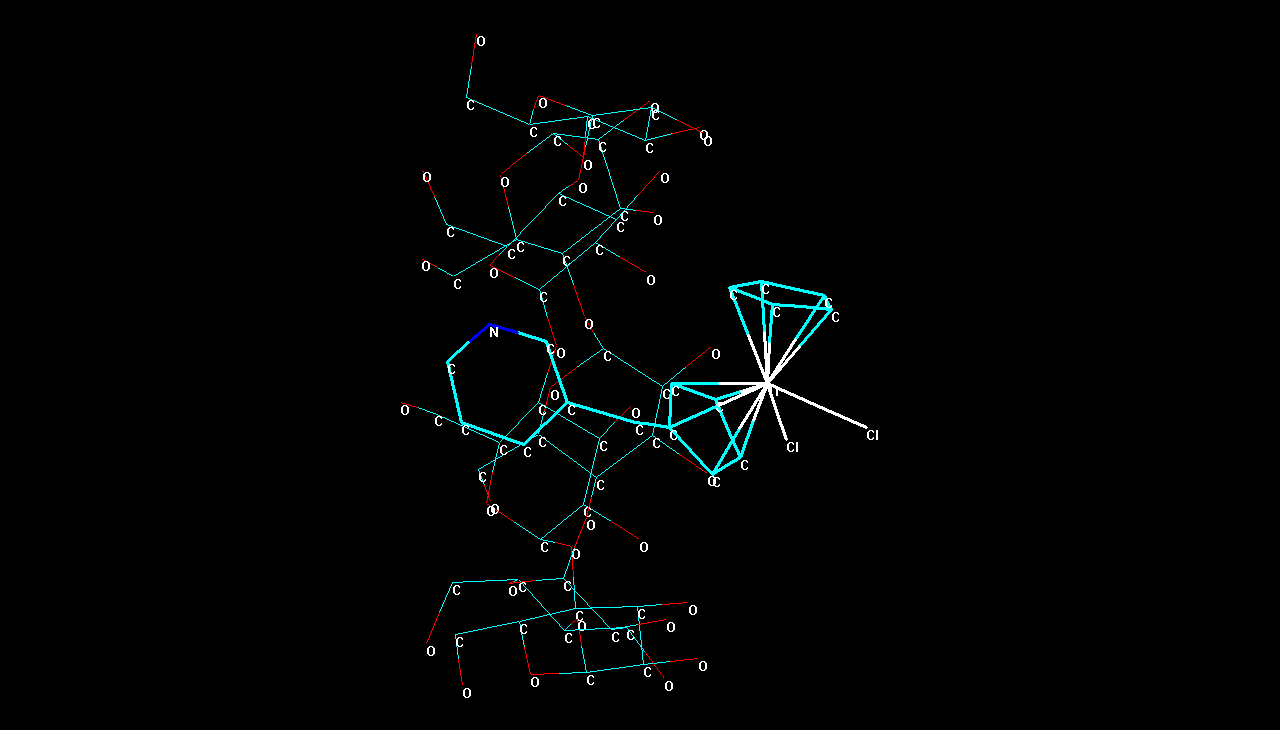


**Figure A3.26.** Starting position (up) and the minimum energy supramolecular system (down) for titanocene (code 23TC) / β-cyclodextrin complex (theoretically modeled by MM+ docking experiments)


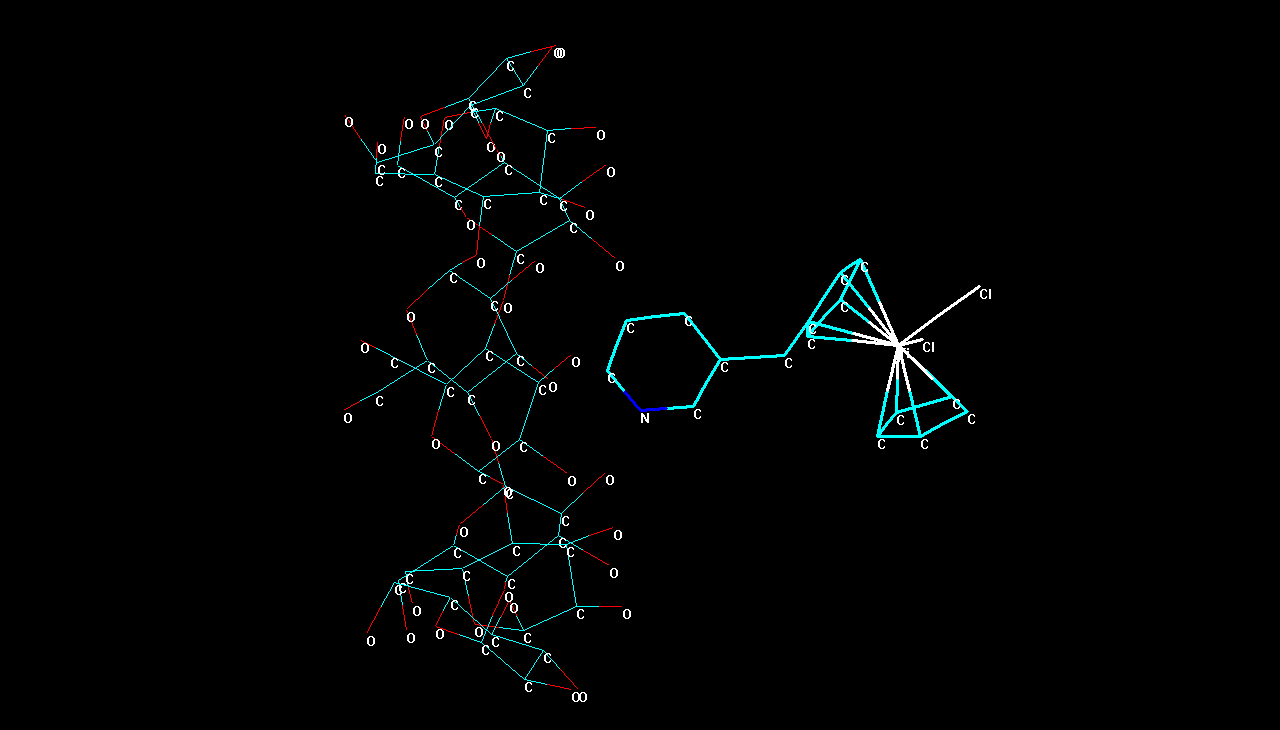


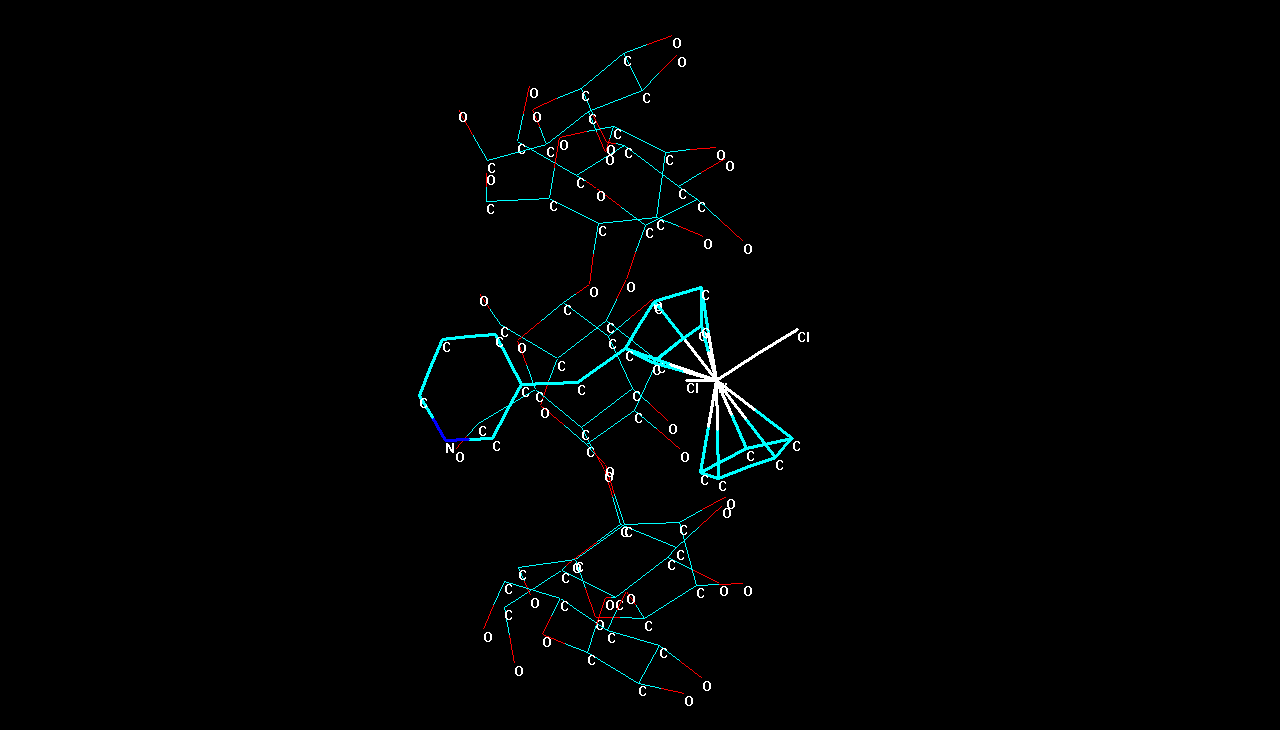


**Figure A3.27.** Starting position (up) and the minimum energy supramolecular system (down) for titanocene (code 23TC) / γ-cyclodextrin complex (theoretically modeled by MM+ docking experiments)


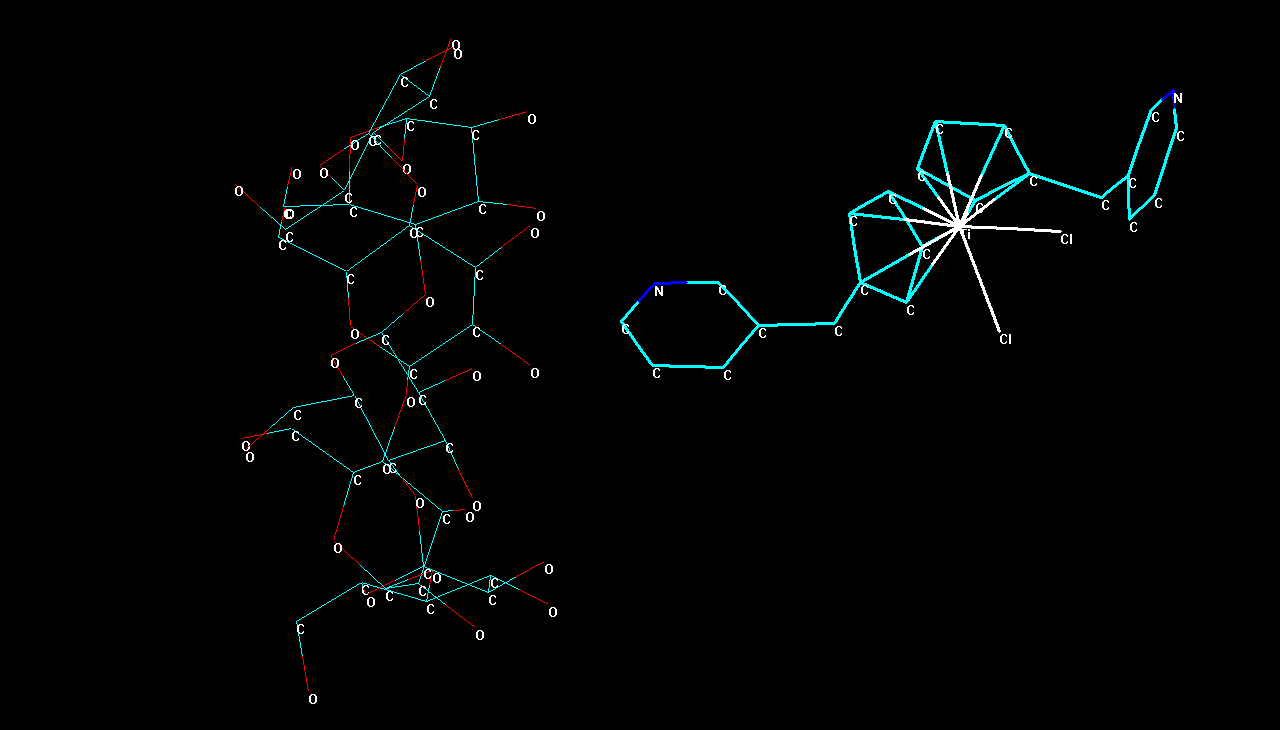


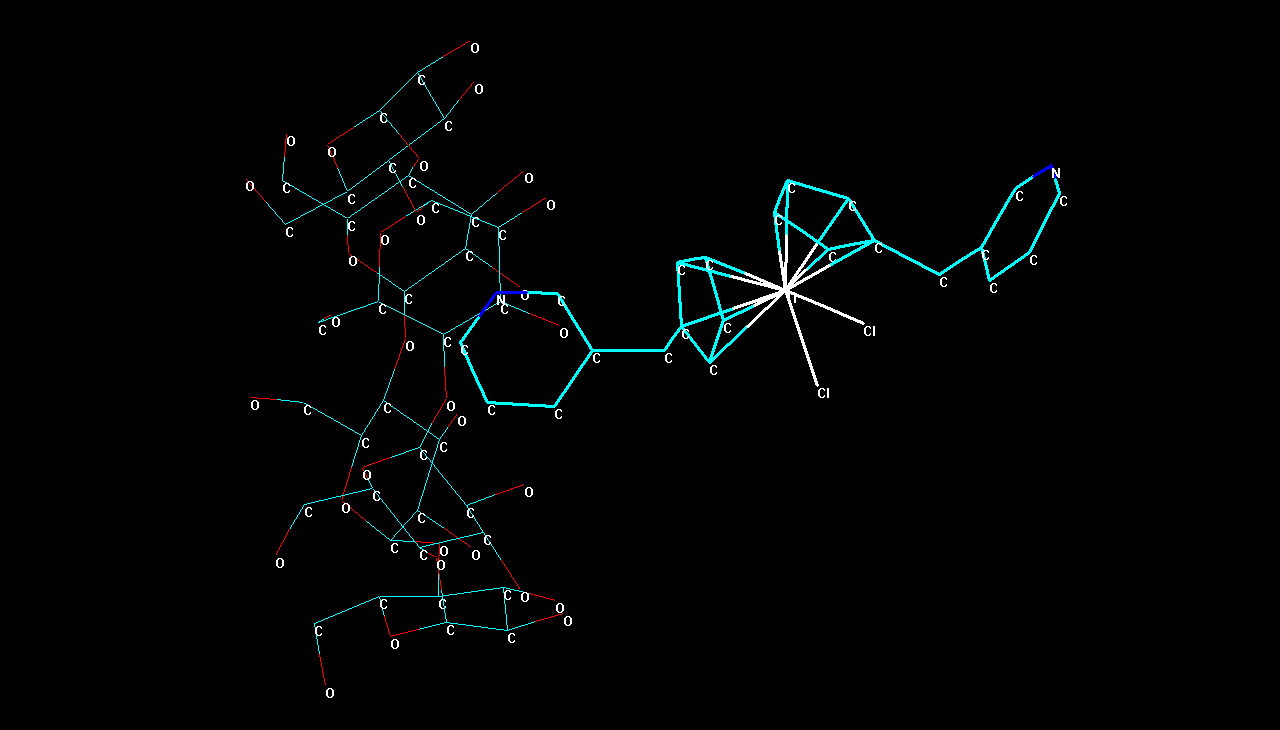


**Figure A3.28.** Starting position (up) and the minimum energy supramolecular system (down) for titanocene (code 24TC) / α-cyclodextrin complex (theoretically modeled by MM+ docking experiments)


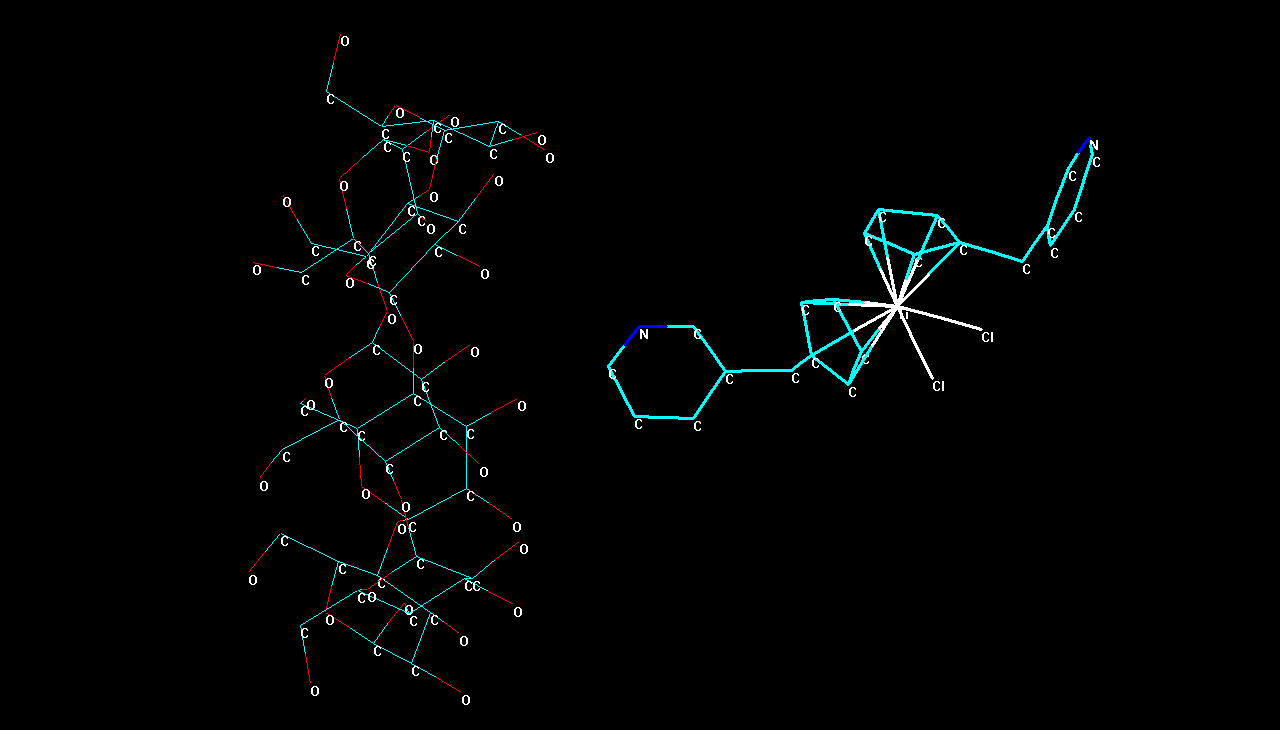


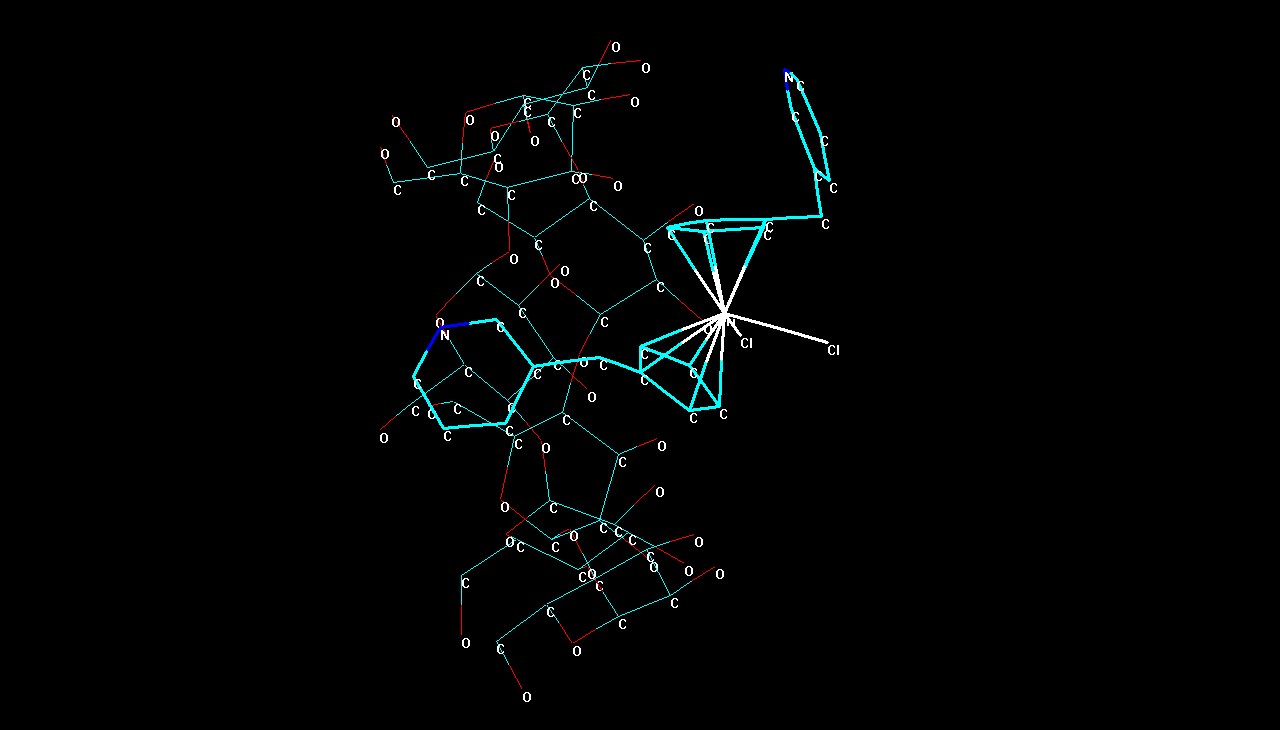


**Figure A3.29.** Starting position (up) and the minimum energy supramolecular system (down) for titanocene (code 24TC) / β-cyclodextrin complex (theoretically modeled by MM+ docking experiments)


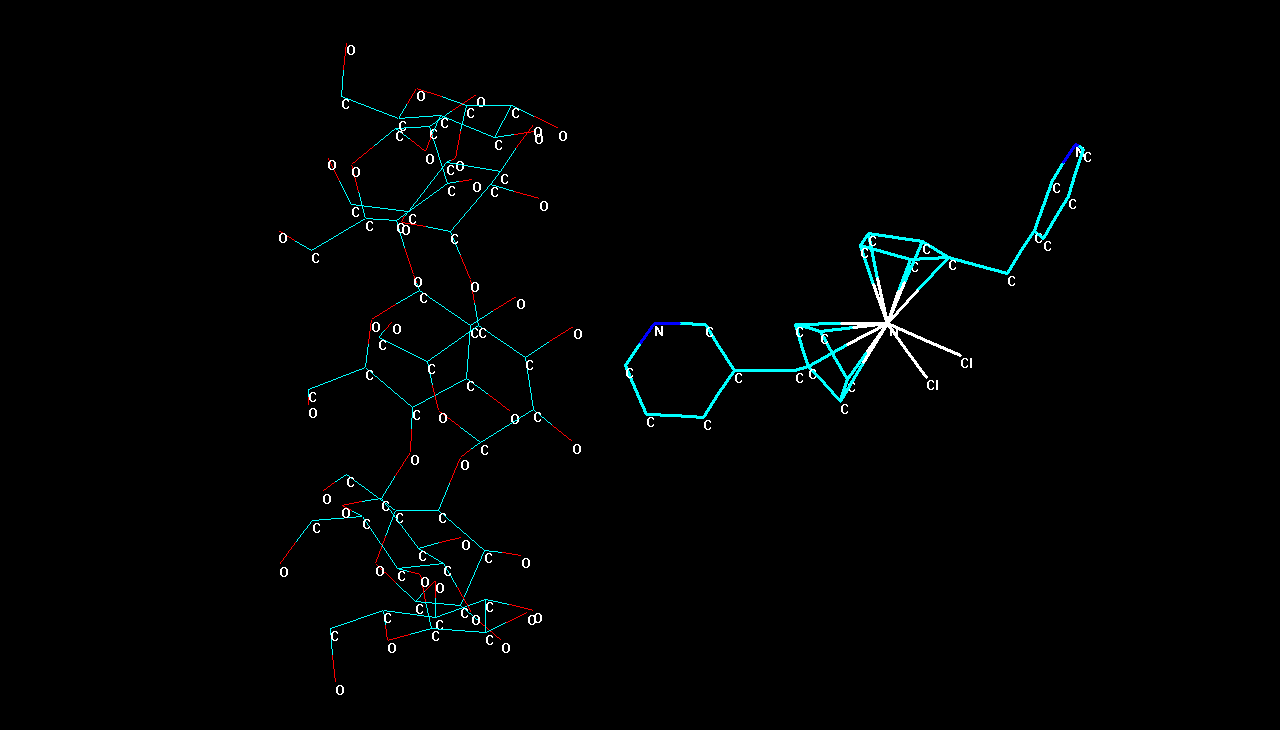


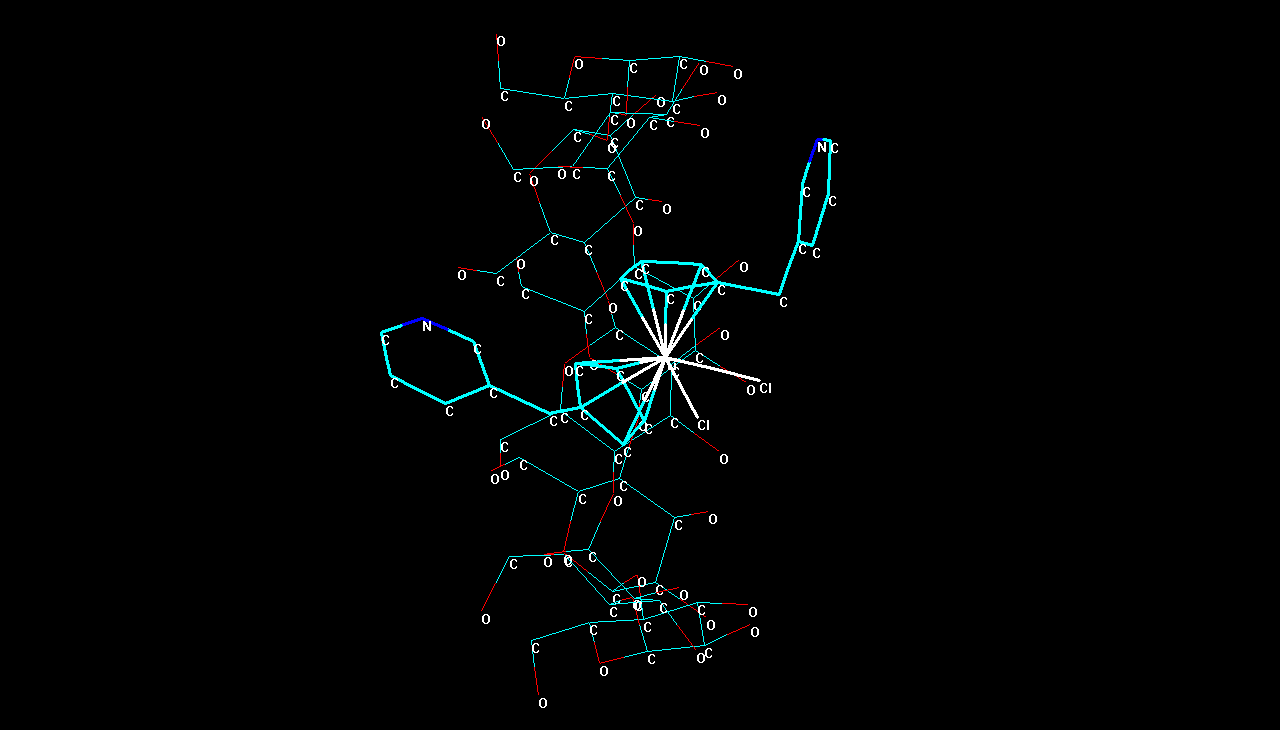


**Figure A3.30.** Starting position (up) and the minimum energy supramolecular system (down) for titanocene (code 24TC) / γ-cyclodextrin complex (theoretically modeled by MM+ docking experiments)


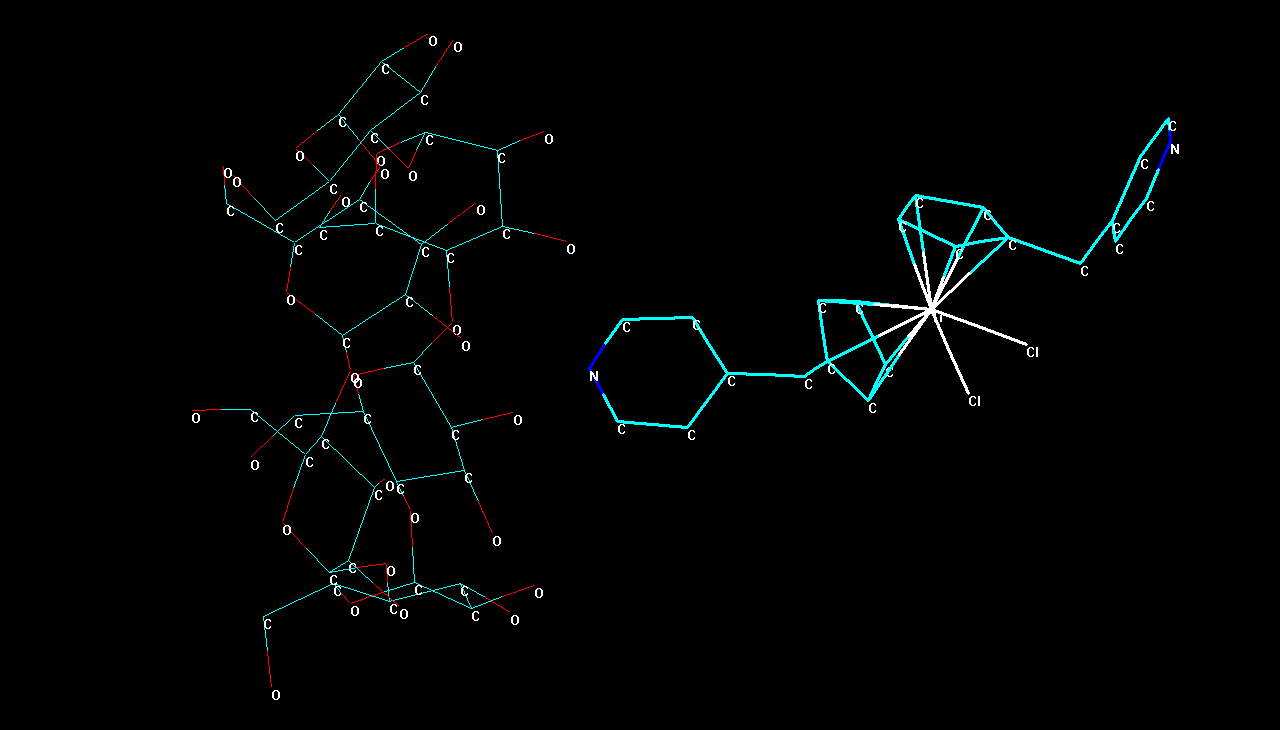


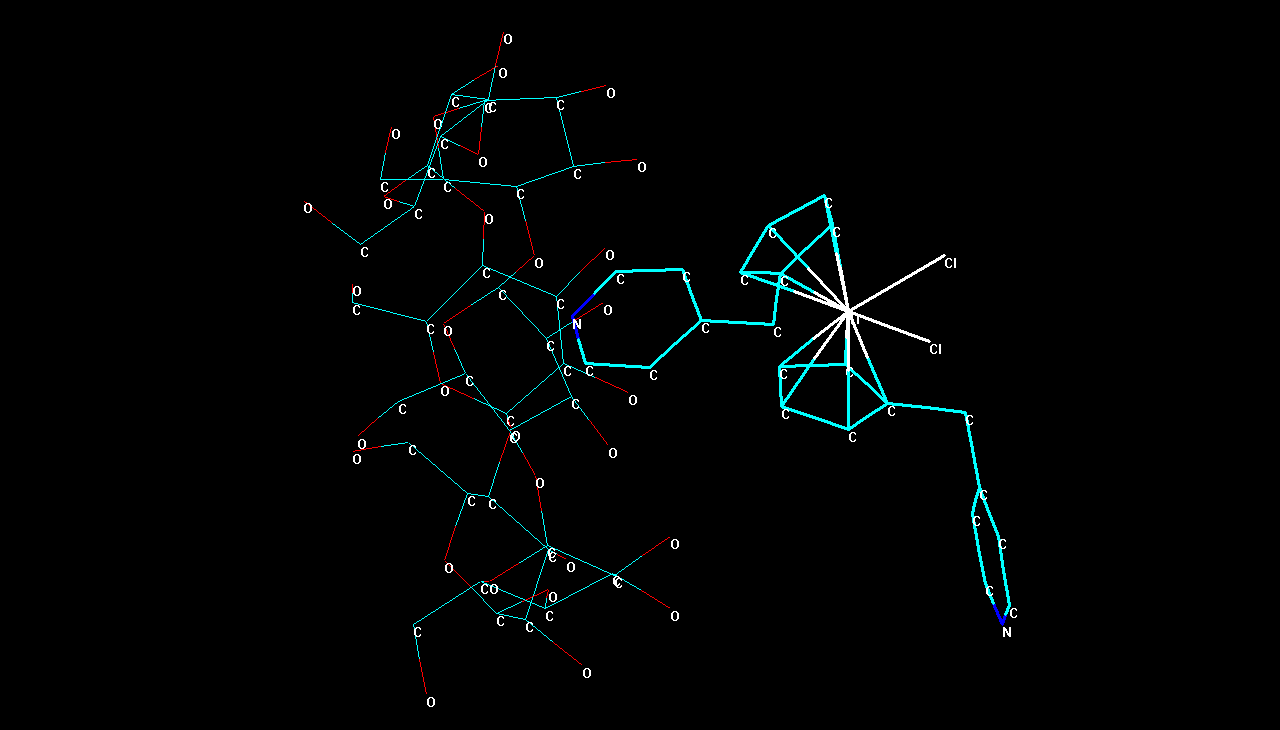


**Figure A3.31.** Starting position (up) and the minimum energy supramolecular system (down) for titanocene (code 26TC) / α-cyclodextrin complex (theoretically modeled by MM+ docking experiments)


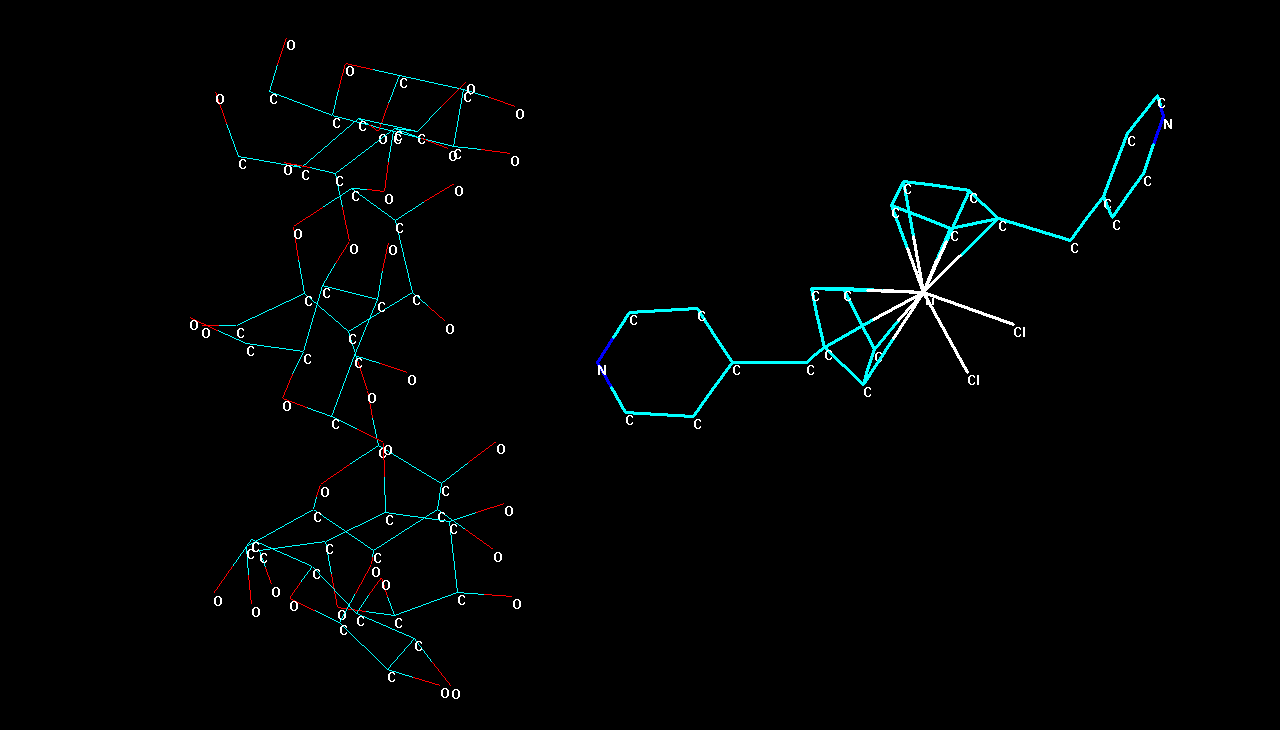


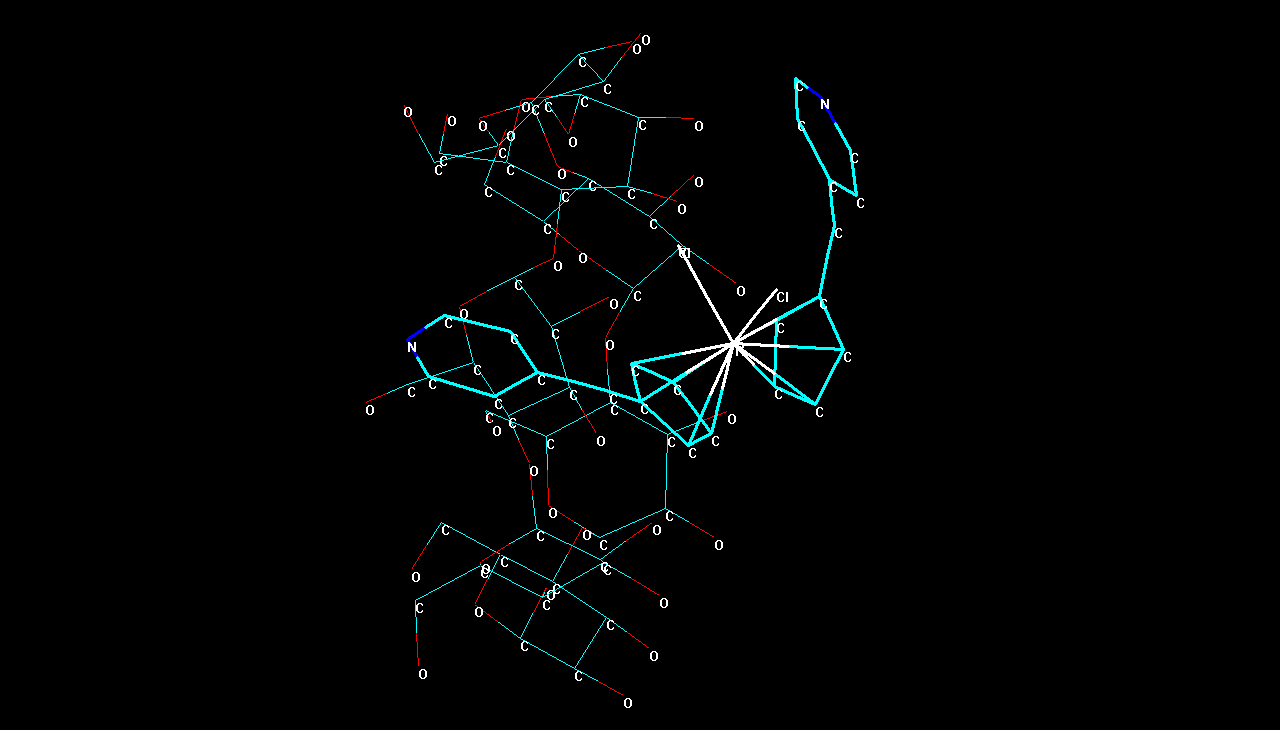


**Figure A3.32.** Starting position (up) and the minimum energy supramolecular system (down) for titanocene (code 26TC) / β-cyclodextrin complex (theoretically modeled by MM+ docking experiments)


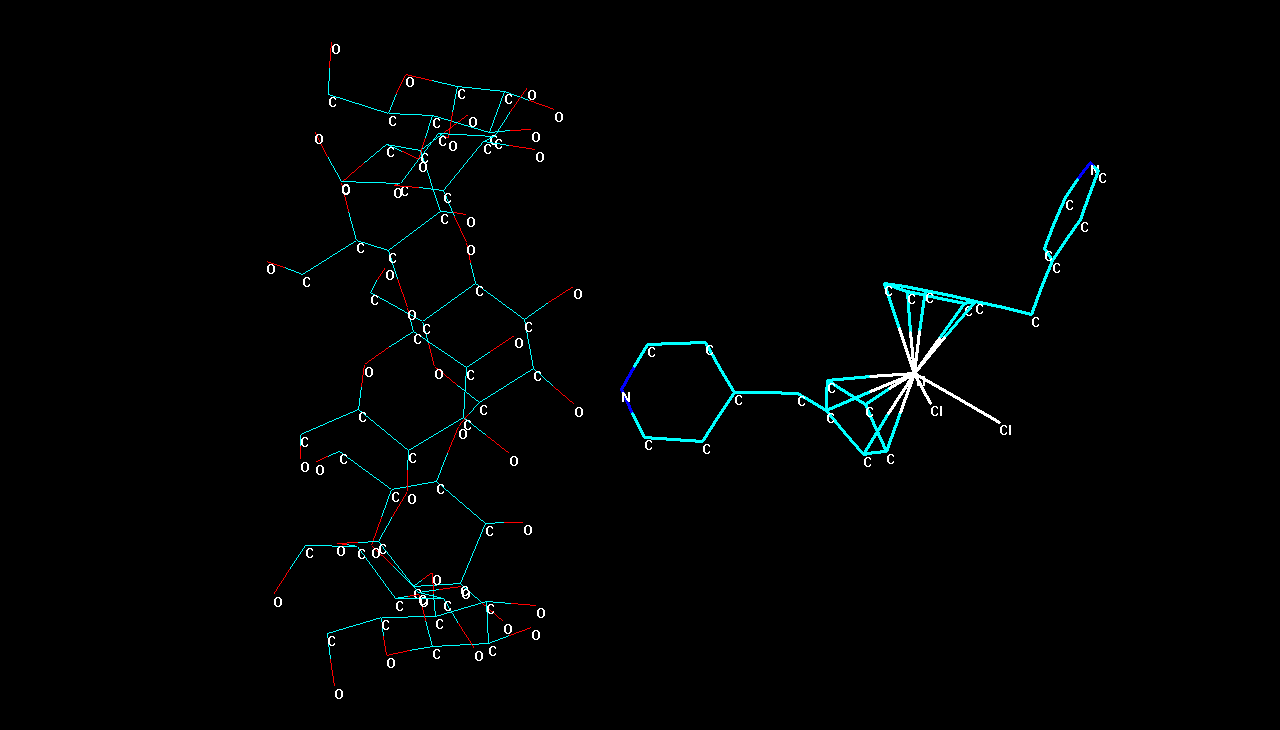


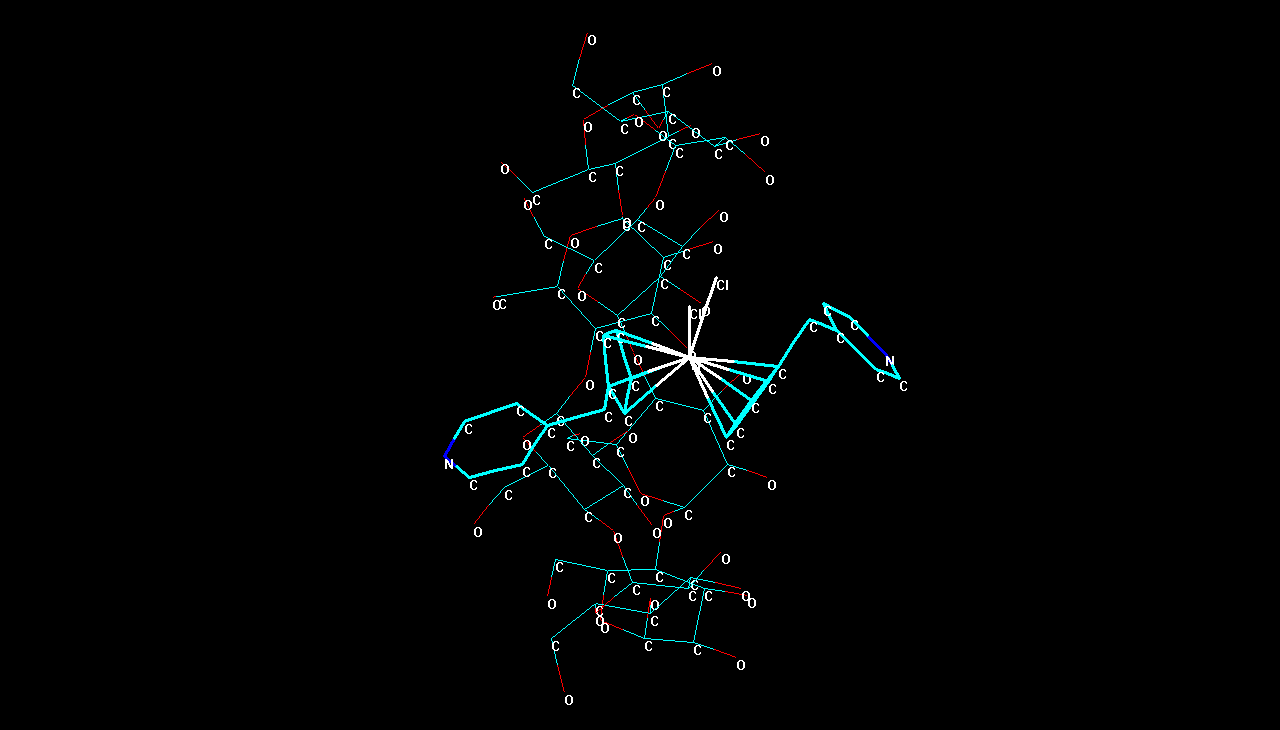


**Figure A3.33.** Starting position (up) and the minimum energy supramolecular system (down) for titanocene (code 26TC) / γ-cyclodextrin complex (theoretically modeled by MM+ docking experiments)

***A3.2. Variation of the titanocene / cyclodextrin interaction energy***

***(E_interaction_, kcal/mole) in the complexation process:***

**Figure A3.34.** Interactioen energy *vs.* Number of cycles from MM+ docking experiments for 01TC_CDs supramolecular systems

**Figure A3.35.** Interactioen energy *vs.* Number of cycles from MM+ docking experiments for 02TC_CDs supramolecular systems

**Figure A3.36.** Interactioen energy *vs.* Number of cycles from MM+ docking experiments for 03TC_CDs supramolecular systems

**Figure A3.37.** Interactioen energy *vs.* Number of cycles from MM+ docking experiments for 08TC_CDs supramolecular systems

**Figure A3.38.** Interactioen energy *vs.* Number of cycles from MM+ docking experiments for 09TC_CDs supramolecular systems

**Figure A3.39.** Interactioen energy *vs.* Number of cycles from MM+ docking experiments for 10TC_CDs supramolecular systems

**Figure A3.40.** Interactioen energy *vs.* Number of cycles from MM+ docking experiments for 11TC_CDs supramolecular systems

**Figure A3.41.** Interactioen energy *vs.* Number of cycles from MM+ docking experiments for 18TC_CDs supramolecular systems

**Figure A3.42.** Interactioen energy *vs.* Number of cycles from MM+ docking experiments for 23TC_CDs supramolecular systems

**Figure A3.43.** Interactioen energy *vs.* Number of cycles from MM+ docking experiments for 24TC_CDs supramolecular systems

**Figure A3.44.** Interactioen energy *vs.* Number of cycles from MM+ docking experiments for 26TC_CDs supramolecular systems

***A3.3. Titanocene / cyclodextrin interaction energies correlations:***

$E_{int.,gCD,i}=16.15\left(\pm2.80\right)+0.51\left(\pm0.14\right)\cdot E_{int.,bCD,i}$ [Eq. A3.1]

*n* = 9 (except 18TC and 23TC); *r* = 0.815; *F* = 13.8
